# Improved and ligand-free copper-catalyzed cyclization for an efficient synthesis of benzimidazoles from *o*-bromoarylamine and nitriles†

**DOI:** 10.1039/d4ra00245h

**Published:** 2024-02-26

**Authors:** Emmanuel Mintah Bonku, Hongjian Qin, Abdullajon Odilov, Safomuddin Abduahadi, Samuel Desta Guma, Feipu Yang, Fuqiang Zhu, Haji A. Aisa, Jingshan Shen

**Affiliations:** a State Key Laboratory of Drug Research, Shanghai Institute of Materia Medica, Chinese Academy of Sciences 555 Zuchongzhi Road Shanghai 201203 P. R. China shenjingshan@simm.ac.cn; b University of Chinese Academy of Sciences No. 19A Yuquan Road Beijing 100049 P. R. China; c State Key Laboratory Basis of Xinjiang Indigenous Medicinal Plants Resource Utilization, Xinjiang Technical Institute of Physics and Chemistry, Chinese Academy of Sciences Urumqi Xinjiang 830011 P. R. China haji@ms.xjb.ac.cn; d Topharman Shanghai Co., Ltd. No. 388 Jialilue Road, Zhangjiang Hitech Park Shanghai 201203 P.R. China

## Abstract

We present an improved copper-catalyzed cyclization for an efficient synthesis of benzimidazoles from *o*-bromoarylamine and nitriles, under mild and ligand-free conditions. The optimal conditions yielded exceptional products of up to 98%, demonstrating the broad applicability of this synthetic strategy in generating a wide range of valuable imidazole derivatives. This methodology enables the efficient synthesis of various substituted benzimidazole derivatives and offers an environmentally friendly alternative to conventional methods. By eliminating the use of harsh reagents and high temperatures associated with traditional synthesis approaches, this method proves to be more efficient and robust. Notably, we successfully applied this synthetic approach to the synthesis of bendazol and thiabendazole, yielding 82% and 78%, respectively, on a 100 gram scale.

## Abstract 2

A ligand-free copper-catalyzed cyclization method is shown for synthesizing benzimidazoles and optimized process of benzimidazoles from cheap *o*-bromoarylamine and nitriles.

## Graphical abstract

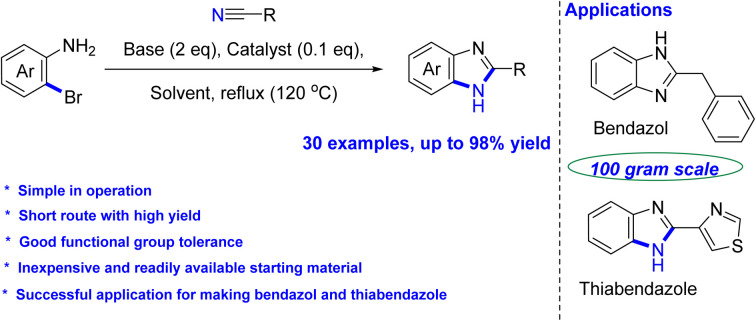

## Introduction

**Fig. 1:**
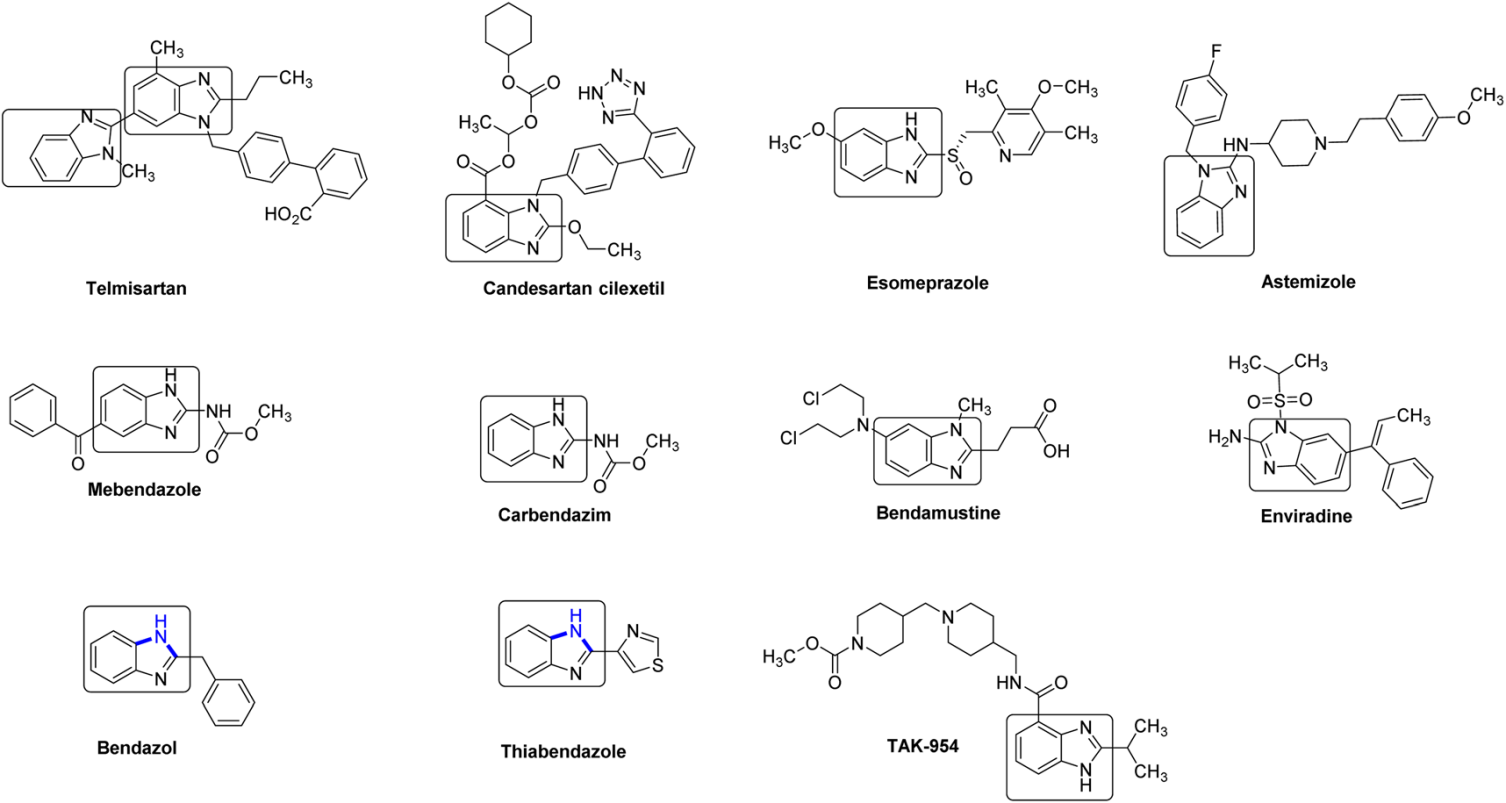
Selected examples of drugs containing benzimidazole ring unit.

Benzimidazole (2) and its derivatives are crucial nitrogen-containing heterocycles that serve as privileged scaffolds in the pharmaceutical industry.^1^ This is primarily due to their prominence in various bioactive compounds and pharmaceutical active ingredients (APIs). These include antihypertensive drugs like telmisartan, bendazol, and candesartan, as well as antihelminthic drugs such as albendazole, thiabendazole, and mebendazole. Additionally, benzimidazoles are utilized in the production of antiviral drugs like enviradine, proton pump inhibitors such as esomeprazole, lansoprazole, and pantoprazole, then as antihistamines like semizole (Fig. 1).^2^ Due to the significant biological activities, unique structural features, and wide-ranging industrial applications, the synthesis of benzimidazole-based drugs has garnered considerable attention from chemists, resulting in the development of various strategies.^2^ One of the key focal points has been the efficient construction of the essential benzimidazole skeleton.

**Scheme 1:**
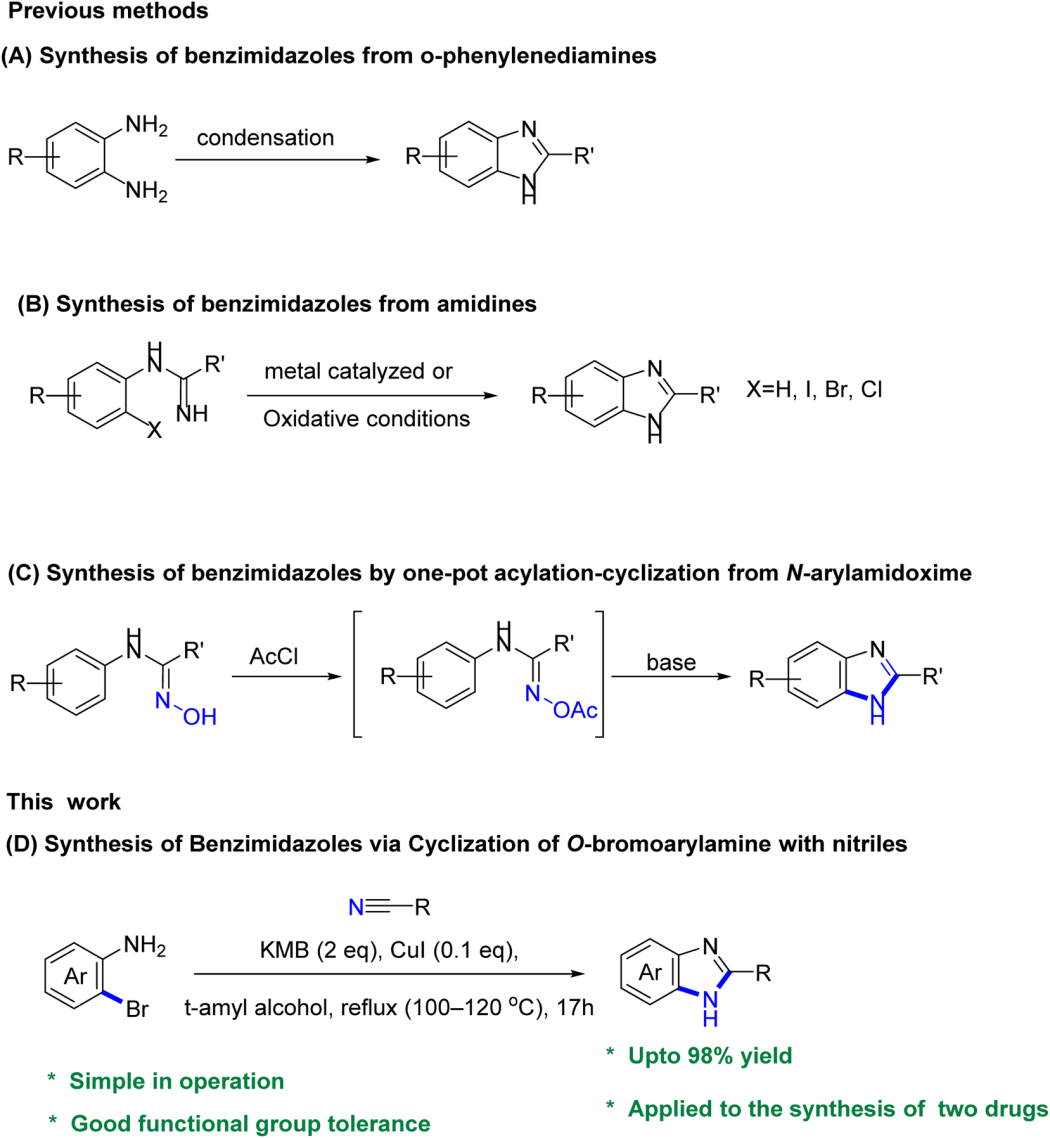
Previous methods (A–C) for producing benzimidazoles, and our method (D) for the synthesis of benzimidazoles *via* cyclization of *o*-bromoarylamine with nitriles.

The conventional method for synthesizing benzimidazoles involves the condensation and cyclization of substituted *o*-phenylenediamines with carboxylic acid derivatives and aldehydes under strong acidic conditions, high temperatures, or with equivalent amounts of oxidants. Additionally, the production of the required substituted *o*-phenylenediamines typically necessitates the incorporation of nitro groups through nitration with nitrosulfuric acid on the substituting aniline, followed by reduction (Scheme 1A). Several alternative approaches have been evaluated to improve the synthesis of benzimidazoles, including the use of aldehydes as substrates and iodine as a catalyst in the presence of hydrogen peroxide,^3^ employing metal-catalyzed or oxidative cyclization of amidines (Scheme 1B),^4^ and the use of an iridium photocatalyst to generate benzimidazoles from *N*-phenylamidoxime esters (Scheme 1C), and a recently reported approach involving Pd-catalyzed carbonylative cyclization.^5,6^ However, certain underlying issues remain unresolved or new drawbacks have emerged, such as the commercial unavailability of viable raw materials, laborious procedures, the need for homogeneous precious-metal catalysts, and high cost. Moreover, these approaches generate significant amounts of stoichiometric waste due to the presence of leaving groups or additives in the substrate. Furthermore, the original synthetic route for constructing benzimidazole intermediates, which involved nitration and polyphosphoric acid (PPA)-mediated cyclization, resulted in safety issues and the generation of acidic sewage that required treatment.^7^

In prior research, various synthesis methods for benzimidazoles have been reported (Scheme 1A–C), including the copper-catalyzed Ullman-type C–N coupling of *o*-haloanilides with primary amides in a one-pot reaction.^8^ It's worth noting that Xiang *et al.*^9^ and Yu *et al.*^10^ demonstrated the synthesis of benzimidazoles through intermolecular cyclization reactions of 2-iodoanilines with aryl nitriles. Although their approaches offers route to synthesize a range of amides and benzoxazole derivatives, its application in terms of catalytic synthesis of heterocycles is limited. More so, using nitriles as nitrogen nucleophiles, the direct reaction with arylamines through a catalytic cyclization system streamlines the synthesis of benzimidazoles compared to that with amides, and even with ligands.

In light of previous studies and existing knowledge, particularly Xiang *et al.*^9^ and Yu *et al.*,^10^ we were inspired to investigate the feasibility of employing nitriles as an alternative to amides for the synthesis of benzimidazoles, and expand the substrate scope through the cyclization of anilines with nitriles to generate the desired product (Scheme 1D), and further demonstrate the synthetic application of our approach in a number of benzimidazole-based drugs.

Herein, we present an improved approach for the synthesis of benzimidazoles by cyclization of *o*-bromoarylamine with nitriles. Remarkably, the reaction occurs under mild and ligand-free conditions, using a relatively low catalytic load. This methodology utilizes readily accessible starting materials, thus promoting a safe and efficient synthesis pathway. It is noteworthy that this method exhibits a high tolerance for functional groups on the substrate and enables the efficient construction of structurally diverse benzimidazoles compared to previous approaches. In addition, this method provided an improved approach for making bendazol and thiabenzadole, avoiding the undesired cyclization with polyphosphoric acid, high reaction temperature, and other drawbacks in the existing operations.

## Results and discussion

The cyclization reaction conditions for the formation of benzimidazole (2) were investigated in this study, with a focus on the reaction between anilines and *n*-butyronitrile (3) as our model starting materials. We specifically chose anilines due to their cleaner and more promising alternative to other aromatic amines. Previous reports corroborate that anilines are readily available, cost-effective, and easy to handle.^11^ Consequently, we anticipated that the cyclization process with anilines under mild conditions would offer significant advantages by reducing the reliance on harsh reagents and conditions typically associated with traditional methods.

In our investigation, we initially explored the synthesis of benzimidazoles using the cyclization reaction of 2-iodoaniline (1I) and arylnitrile, as previously reported by Xiang's^9^ and Yu's^10^ research group. Building upon this method, we aimed to directly cyclize *o*-bromoaniline (1Br) or *o*-chloroaniline (1Cl) with *n*-butyronitrile in the absence of copper catalyst and ligand. However, our experimental results indicated that the desired product was only obtained in limited quantities (Table 1, entries 1–3).

**Table 1:** Optimization of the cyclization reaction for benzimidazole using *o*-bromoaniline with *n*-butyronitrile^a^

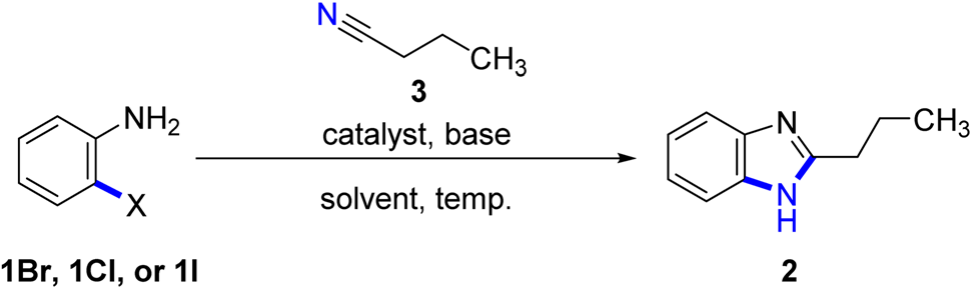							
Entry	Starting material	Catalyst	Base	Solvent	Temp. (°C)	Time (h)	Yield^b^ (%)
1	2-Iodoaniline	—	*t*-BuOK	*t*-BuOH	84	17	14
2	2-Chloroaniline	—	*t*-BuOK	*t*-BuOH	84	17	Trace
3	*o*-Bromoaniline	—	*t*-BuOK	*t*-BuOH	84	17	26
4	*o*-Bromoaniline	CuBr	*t*-BuOK	*t*-BuOH	84	17	80
5	*o*-Bromoaniline	CuBr	*t*-BuOK	*t*-BuOH	84	17	39^c^
6	*o*-Bromoaniline	CuCl	*t*-BuOK	*t*-BuOH	84	15	68
7	*o*-Bromoaniline	Cu_2_O	*t*-BuOK	*t*-BuOH	84	15	71
8	*o*-Bromoaniline	CuBr_2_	*t*-BuOK	*t*-BuOH	84	16	41
9	*o*-Bromoaniline	CuCl_2_	*t*-BuOK	*t*-BuOH	84	16	35
10	*o*-Bromoaniline	Cu(OAc)_2_	*t*-BuOK	*t*-BuOH	84	16	38
11	*o*-Bromoaniline	CuI	*t*-BuOK	*t*-BuOH	84	16	86
12	*o*-Bromoaniline	CuI	KOH	*t*-BuOH	84	16	Trace
13	*o*-Bromoaniline	CuI	K_2_CO_3_	*t*-BuOH	84	16	Nd^d^
14	*o*-Bromoaniline	CuI	K_3_PO_4_	*t*-BuOH	84	16	Nd^d^
15	*o*-Bromoaniline	CuI	*t*-BuONa	*t*-BuOH	84	16	64
16	*o*-Bromoaniline	CuI	(*t*-BuO)_2_Mg	*t*-BuOH	84	16	55
17	*o*-Bromoaniline	CuI	*t*-BuOLi	*t*-BuOH	84	16	74
18	*o*-Bromoaniline	CuI	KMB	*t*-BuOH	84	18	87
19	*o*-Bromoaniline	CuI	KMB	1,4-Dioxane	100	18	56
20	*o*-Bromoaniline	CuI	KMB	Toluene	110	16	45
21	*o*-Bromoaniline	CuI	KMB	DMF	140	17	50
22	*o*-Bromoaniline	CuI	KMB	NMP	140	18	55
23	*o*-Bromoaniline	CuI	KMB	*t*-Amyl alcohol	120	17	92
24	*o*-Bromoaniline	CuI	KMB	*t*-Amyl alcohol	120	17	93^e^
25	2-Chloroaniline	CuI	KMB	*t*-Amyl alcohol	120	17	20
26	2-Iodoaniline	CuI	KMB	*t*-Amyl alcohol	120	17	85
27	*o*-Bromoaniline	CuI	KMB	*t*-Amyl alcohol	120	17	86^f^
28	*o*-Bromoaniline	CuI	KMB	*t*-Amyl alcohol	120	17	85^f^
29	*o*-Bromoaniline	CuI	KMB	*t*-Amyl alcohol	120	17	Trace^g^
30	*o*-Bromoaniline	CuI	KMB	*t*-Amyl alcohol	120	17	43^c^

a Reactions were conducted by dissolving compound 1Br, 1Cl or 1I (1.0 mmol) in solvent (6 ml), then adding *n*-butyronitrile 3 (2 equiv.), catalysts (0.1 equiv.) and a base (2.0 equiv.), followed by stirring at 84–140 °C for 17 h.

b Isolated yield.

c With 1 equivalent of base.

d Not detected.

e Stirring for 36 h.

f Addition of ligand.

g With 0.5 equivalent of base.

Based on the aforementioned observations, we hypothesized that the desired product could be obtained through the cyclization reaction of *o*-bromoaniline (1Br) with nitriles under optimized conditions (Table 1, entry 3). Hence, our investigation focused on determining the optimal conditions for this cyclization reaction, starting with an evaluation of the base and catalyst's effect.

When the reaction was conducted in the absence of a base, the desired product was not obtained. Moreover, reducing the equivalent of the base resulted in a decreased yield compared to the other case (entry 5 *vs.* 4).

Again, our findings revealed that the inclusion of a copper catalyst was essential, as its absence led to a significant decrease in product yield and a notable unconsumed amount of starting material (Table 1, entries 1–3, *vs.* 4–11). We attempted the addition of different copper catalysts to react in *t*-BuOH as a solvent to coordinate with *o*-bromoaniline. We found that copper(i) bromide (CuBr), a univalent copper catalyst, could afford the desired product in 80% yield (Table 1, entry 4). We also examined the catalytic effects of cuprous chloride (CuCl) and cuprous oxide (Cu_2_O). However, it was observed that these catalysts led to a decreased yield compared to CuBr (Table 1, entries 6–7, *vs.* 4). In addition to the previously tested copper catalysts, we also investigated divalent copper catalysts such as copper bromide (CuBr_2_), copper chloride (CuCl_2_), and copper acetate (Cu(OAc)_2_). However, in comparison to the monovalent copper catalysts, these divalent copper catalysts showed lower catalytic efficiency (Table 1, entries 8–10 *vs.* 4, 6 and 7). After screening various copper catalysts, it was determined that copper iodide (CuI) exhibited the best catalytic effect (Table 1, entry 11). The yield of the cyclization product using copper iodide reached 86%.

Several bases were tested as alternatives to potassium *tert*-butoxide (*t*-BuOK) in our study, including potassium hydroxide (KOH), potassium carbonate (K_2_CO_3_), and potassium phosphate (K_3_PO_4_). However, the use of these bases (Table 1, entries 12–14) yielded only trace amounts or no detectable cyclization product. We also explored the use of other bases such as sodium *tert*-butoxide (*t*-BuONa), magnesium di-*tert*-butoxide ((*t*-BuO)_2_Mg), and lithium *tert*-butoxide (*t*-BuOLi), which were found to promote the reaction and generate the desired products. Nevertheless, their efficiency was comparatively lower than that of potassium *tert*-amylate (KMB) (Table 1, entries 15–17 *vs.* 18). We investigated the effect of different solvents on the cyclization reaction. Various solvents, including *tert*-amyl alcohol, ethylene glycol, dioxane, toluene, dimethylformamide (DMF), and *N*-methylpyrrolidone (NMP), were examined. Among these screened solvents, *tert*-amyl alcohol was found to be more effective than other solvents (Table 1, entry 23 *vs.* 19–22). Under the same reaction conditions, the yield did not increase significantly with the extension of reaction time (Table 1, entry 24). It was observed that, under same conditions, the reaction of 2-chloroaniline and 2-iodoaniline resulted in the desired product with yields of 20% and 85% respectively. Nonetheless, it is evident that 2-bromoaniline proves to be the optimal 2-haloaniline derivative for this reaction (Table 1, entry 23 *vs.* 25–26).

Interestingly, when attempting to add *N*,*N*-dimethylethylenediamine or proline as a ligand, the yield rather decreased (Table 1, entries 27–28). The results clearly indicate that maintaining a reaction time of 16–17 hours without the use of ligands is the optimal approach. Therefore, we selected copper iodide as the catalyst, potassium *tert*-amylate (KMB) as the base, and a refluxing (120 °C) cyclization reaction in *tert*-amyl alcohol as the optimal reaction conditions (Table 1, entry 23).

Additional control experiments were conducted under optimized conditions, specifically without a base, resulting in no detectable cyclization product. Using less than 2 equivalent of the base resulted in a trace amount (entry 29) and low yield of the desired product, indicating a lower catalytic efficiency as compared to other yield (entry 30 *vs.* 23). These results unequivocally demonstrate that copper-catalyzed cyclization reactions require a substantial amount of base, along with a catalytic amount of copper catalyst, to produce the desired benzimidazoles.^12^

Following the establishment of the optimal cyclization reaction conditions, we proceeded to explore the scope and versatility of aliphatic nitrile substrates and *o*-bromoaromatic amines (Table 2). Remarkably, our research revealed that when the benzene ring of the *o*-bromoaromatic amine substrate (1a–1c, 1f, 1k, 1o) was substituted with a methyl group or an ester, the reaction proceeded smoothly, leading to the formation of the desired products (2a–2c, 2f, 2k, 2o) with yields ranging between 86-95%. We further explored the influence of different substituents on the cyclization reaction by investigating the electronic effects of substituents on the benzene ring of *o*-bromoaromatic amine. Remarkably, the presence of a methoxy group in the phenyl substrates (1d, 1e) resulted in efficient cyclization reactions, yielding benzimidazoles (2d and 2e) with a high yield of 98%. Moreover, when the phenyl *o*-bromoaromatic amine substrates (1g–1h) contained chlorine or fluorine substituents, the cyclization products (2g and 2h) were obtained in even higher yields of 94% and 88%, respectively.

**Table 2:** Substrate scope of the cyclization reaction for benzimidazoles^a^

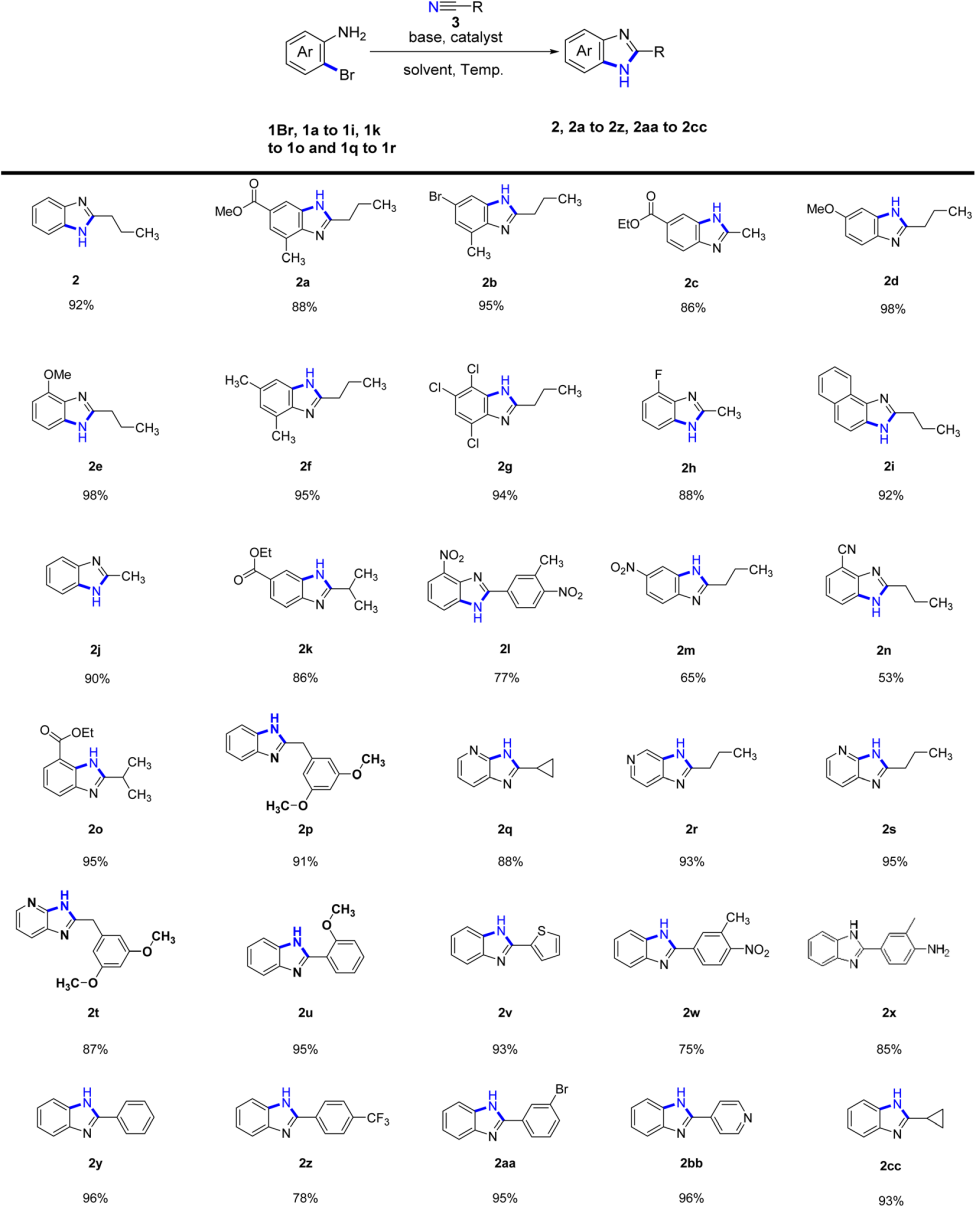

a The reactions were performed on the scale of 1.0 mmol of 1Br, 1a–1i, 1k–1o and 1q–1r under the conditions: 2.0 equiv. of nitriles, 2 equiv. of KMB, 0.1 equiv. of CuI catalyst, 6 ml *t*-amyl alcohol solvent, 120 °C for 17 h. The yields were given as isolated yields.

Furthermore, we successfully investigated the reactivity of the phenyl unsubstituted *o*-bromoaniline substrate (1Br) in cyclization reactions with various nitriles, resulting in higher yields of products (2, 2j, 2cc, 2p). However, when *o*-bromoaniline substrates with electron-withdrawing groups such as nitro (1m) and nitrile (1n) substituents were used, the yields of the corresponding products (2m, 2n) were lower, ranging from 53% to 65%. Interestingly, *o*-bromoaromatic amines bearing 1-naphthyl (1i) and pyridyl (1q, and 1r) substituents also exhibited reactivity in the intermolecular cyclization reactions, affording products 2i (92% yield), 2q (88% yield), 2r (93% yield), 2s (95% yield) and 2t (87% yield).

Again, an investigation was conducted on the scope of aromatic nitrile substrates and *o*-bromoaromatic amines. The results showed that most aromatic nitrile substrates and *o*-bromoarylamine cyclization yielded satisfactory results. However, it was observed that the presence of charge-drawing functional groups, such as 3-methyl-4-nitrobenzonitrile and *p*-trifluoromethylbenzonitrile, led to lower cyclization yields (2l, 2w, and 2z at 75–78%). Other aromatic nitrile substrates, such as benzonitrile, 2-methoxybenzonitrile, 2-nitrilethiophene, and 3-methyl-4-aminobenzonitrile can all undergo cyclization with *o*-bromoaniline (1Br) to afford the desired products with higher yields. Specifically, the desired products 2y, 2u, 2v, and 2x were obtained with yields of 96%, 95%, 93%, and 85%, respectively. Remarkably, even when 3-bromobenzonitrile was cyclized with *o*-bromoaniline (1Br), the reaction exhibited good functional group compatibility as the bromine atom did not participate in the reaction, leading to the formation of the desired product 2aa with a yield of 95%. Similarly, the reaction of aromatic nitriles with heterocyclic rings, such as 4-nitrilepyridine and *o*-bromoaniline (1Br) successfully gave the desired product 2bb with a yield of 96%.

### Post-application for the synthesis of thiabendazole, and bendazol

Subsequently, the applicability of our cyclization approach was demonstrated in a concise synthesis of antihypertensive drug, bendazol, and anthelminthic drug, thiabenzadole.^2^ Gram-scale cyclization of 2-bromoaniline with thiazole-4-carbonitrile and potassium-2-methylbutan-2-olate in *tert*-amyl alcohol in the presence of CuI at 120 °C afforded thiabendazole with an impressive isolated yield of 78%, surpassing the reported yield of 64% achieved using a catalyzed-polyphosphoric acid cyclization method.^13^ Likewise, this succinct approach was utilized to synthesize the antihypertensive drug, bendazol, yielding a light yellow solid of bendazol in a 82% isolated yield through DCM/hexane re-crystallization, showcasing outstanding functional group compatibility for the cyclization of the benzonitrile substrate as compared to previous report.^14^

**Fig. 2:**
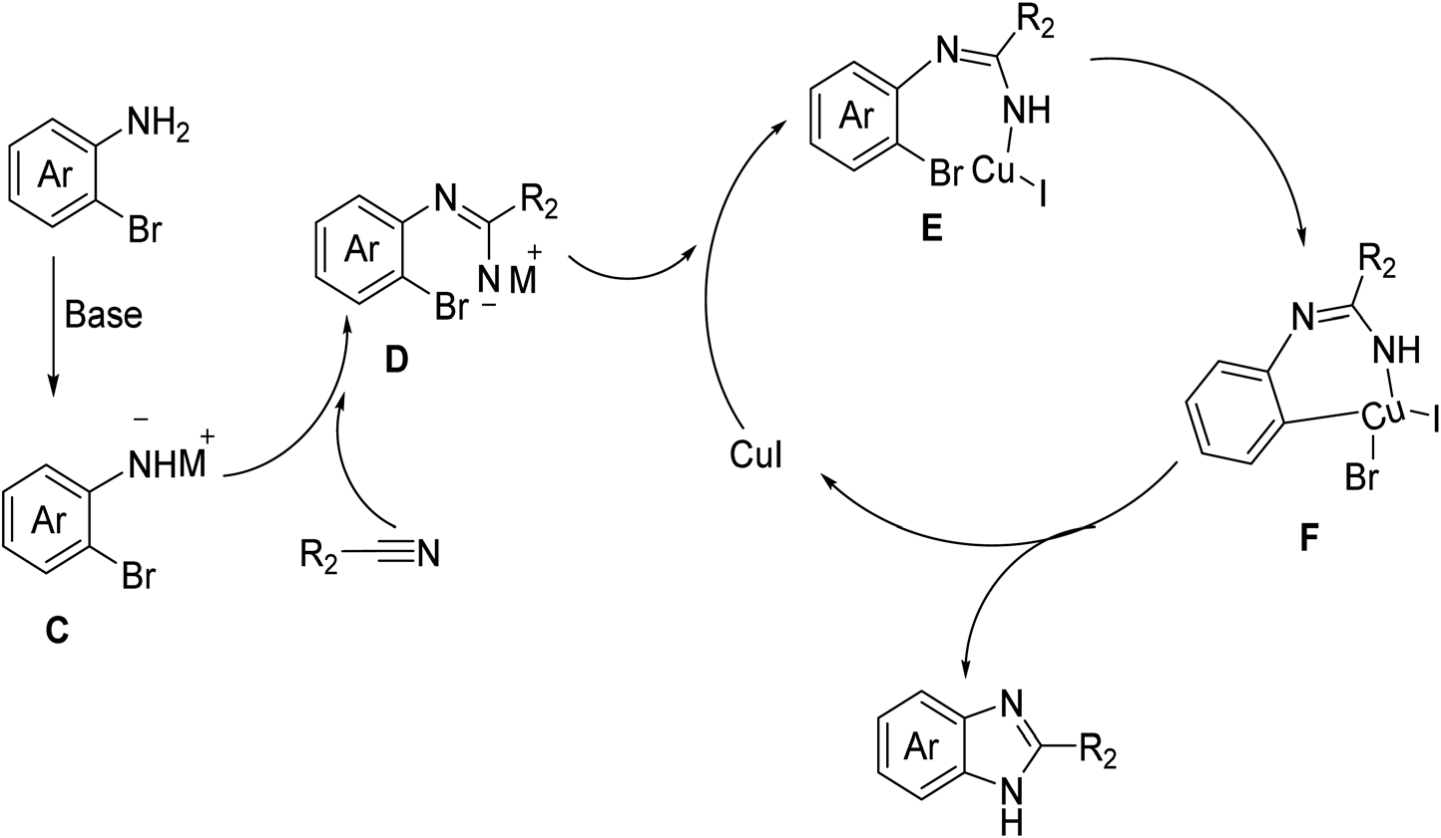
Proposed mechanism of the cyclization reaction between *o*-bromoaromatic amines and nitrile substrates.

Based on the experimental results and relevant literature,^11,13^ we propose a reaction mechanism pathway for the synthesis of benzimidazole compounds *via* the cyclization of *o*-bromoaromatic amines and nitrile substrates, as depicted in Fig. 2. Initially, under basic conditions, the amino group of the *o*-bromoaromatic amine undergoes deprotonation, resulting in the formation of intermediate C with a negatively charged nitrogen atom. Subsequently, it reacts with the cyano group of the nitrile substrate to produce intermediate amidine D. Introducing a copper iodide catalyst leads to the formation of a metal intermediate E. Subsequently, intramolecular bromine transmetalation occurs, resulting in the formation of intermediate F. Ultimately, through reduction and elimination reactions, the final product, benzimidazole compound, is generated, and the catalyst is released, thereby completing the catalytic cycle. These results demonstrate the efficiency of this synthetic pathway for the synthesis of nitrile-based compounds. The findings further demonstrate the broad applicability of the present synthetic strategy in producing valuable imidazole derivatives.

## Conclusions

In this study, we have successfully developed an improved and ligand-free copper-catalyzed cyclization method for the synthesis of benzimidazoles. By utilizing the cyclization of *o*-bromoarylamine with nitriles, we have demonstrated remarkable yields of benzimidazoles, with optimization leading to 30 examples with yields of up to 98%. Our approach builds upon the previous work of Xiang *et al.*^9^ and Yu *et al.*,^10^ offering significant advantages in terms of simplicity, compatibility with various functional groups, substrate scope, and overall efficiency compared to their methods and other existing methodologies. Additionally, we have validated the applicability of our method in the synthesis of two benzimidazole-based drugs, namely bendazol and thiabenzadole, yielding 82% and 78% respectively on a 100 gram scale. Notably, our method eliminates the need for ligands, nitric acid, sulfuric acid, and polyphosphoric acid, emphasizing its versatility and environmentally friendly nature. An ongoing research at our laboratory explores additional applications of this approach.

## Experimental section

### General information

^1^H, ^19^F and ^13^C NMR spectra were recorded on a Brucker 500 Hz or 600 Hz instrument. Chemical shifts for ^1^H NMR are reported in parts per million (ppm), and multiplicities are denoted as follows: s, singlet; d, doublet; t, triplet; q, quartet; m, multiplet; br, broad. Chemical shifts for ^13^C NMR are reported as the chemical shift. Electrospray ionization (ESI) mass spectra were measured using a Thermo Fisher FINNIGAN LTQ instrument. All high-resolution mass spectroscopy (HRMS) results were obtained on an Agilent 1290-6545 UHPLC-QTOF LC/MS spectrometer. TLC inspections were performed on silica gel plates (GF-254). All commercially available chemicals and solvents were used without further purification unless otherwise noted.

### General procedure

*O*-Bromoarylamine (1.0 mmol, 1.0 eq.), potassium-2-methylbutan-2-olate (2.0 mmol, 2 eq.), CuI (0.1 mmol, 0.1 eq.), nitriles 3 (2 mmol, 2 eq.), and *tert*-amyl alcohol (6.0 ml) were charged to a Schleck tube that had been purged with nitrogen. After addition, the mixture was then warmed to 110–120 °C and held for 17 h. The reaction was considered complete when the conversion of *o*-bromoarylamine was ≥99%. The reaction mixture was concentrated in vacuum at 40–45 °C to remove most of the solvents to give the residue. The residue was purified by silica-gel column chromatography to give benzimidazoles.

#### Benzimidazoles 2

Following the general procedure, 2 was obtained from 1 (172 mg, 1.0 mmol). The residue was purified by silica-gel column chromatography (DCM/MeOH = 100/1 to 30/1) to give 2 (147 mg, 92%) as a light yellow solid; m.p. 155–157 °C; ^1^H NMR (500 MHz, CDCl_3_) *δ* 7.92 (brs, 1H), 7.56 (dd, *J* = 6.0, 3.2 Hz, 2H), 7.35–7.16 (m, 2H), 2.93 (t, *J* = 7.6 Hz, 2H), 1.95–1.86 (m, 2H), 1.01 (t, *J* = 7.4 Hz, 3H); ^13^C NMR (125 MHz, CDCl_3_) *δ* 155.6, 138.5, 122.1, 114.6, 31.2, 21.8, 13.8. HRMS (ESI) *m*/*z* [M + H]^+^ calcd for C_10_H_13_N_2_ 161.1073, found 161.1071.

#### Benzimidazoles 2a

Following the general procedure, 2a was obtained from 1a (244 mg, 1.0 mmol). The crude product was purified by silica-gel column chromatography (DCM/MeOH = 100/1 to 20/1) to afford the title compound 2a (204 mg, 88%) as a white solid; m.p. 143–145 °C; ^1^H NMR (400 MHz, CDCl_3_) *δ* 8.87 (brs, 1H), 8.11 (s, 1H), 7.77 (s, 1H), 3.91 (s, 3H), 2.93 (t, *J* = 7.6 Hz, 2H), 2.57 (s, 3H), 1.94–1.83 (m, 2H), 0.96 (t, *J* = 7.4 Hz, 3H); ^13^C NMR (100 MHz, CDCl_3_) *δ* 168.1, 157.3, 141.8, 137.5, 124.6, 124.2, 124.1, 114.3, 52.1, 31.3, 21.8, 17.1, 13.8. HRMS (ESI): *m*/*z* [M + H]^+^ calcd for C_13_H_17_N_2_O_2_ 233.1285, found: 233.1281.

#### Benzimidazoles 2b

Following the general procedure compound 2b was obtained from 1b (264 mg, 1.0 mmol). The crude product was purified by silica-gel column chromatography (DCM/MeOH = 100/1 to 20/1) to afford the title compound 2b (240 mg, 95%) as a white solid; m.p. 125–128 °C; Two sets of ^1^HNMR data representing two isomers (10 : 9) were observed as indicative of the presence of tautomerism; ^1^H NMR (400 MHz, DMSO-*d*_6_, major isomer) *δ* 12.30 (s, 1H), 7.51 (s, 1H), 7.08 (s, 1H), 2.77 (t, *J* = 7.1 Hz, 2H), 2.45 (s, 3H), 1.78 (dt, *J* = 14.3, 7.2 Hz, 2H), 0.94 (t, *J* = 7.4 Hz, 3H); ^13^C{^1^H} NMR (100 MHz, DMSO-*d*_6_, major isomer) *δ* 156.78, 144.90, 130.26, 124.78, 118.41, 113.43, 111.35, 30.90, 21.44, 17.09, 14.16; ^1^HNMR (400 MHz, DMSO-*d*_6_, minor isomer) *δ* 12.24 (s, 1H), 7.40 (s, 1H), 7.08 (s, 1H), 2.77 (t, *J* = 7.1 Hz, 2H), 2.48 (s, 3H), 1.78 (dt, *J* = 14.3, 7.2 Hz, 2H), 0.94 (t, *J* = 7.4 Hz, 3H); ^13^C NMR (101 MHz, DMSO-*d*_6_, minor isomer) *δ* 156.8, 155.6, 144.9, 142.3, 135.4, 133.6, 130.3, 124.9, 124.2, 123.2, 118.4, 113.8, 113.4, 111.3, 30.1, 30.9, 21.4, 17.1, 16.7, 14.1. HRMS (ESI): *m*/*z* [M + H]^+^ calcd for C_11_H_14_BrN_2_ 253.0335, found: 253.0332.

#### Benzimidazoles 2c

Following the general procedure compound 2c was obtained from 1c (244 mg, 1.0 mmol). The crude product was purified by silica-gel column chromatography (DCM/MeOH = 100/1 to 20/1) to afford the title compound 2c (175 mg, 86%) as a white solid; m.p. 172–174 °C; ^1^H NMR (400 MHz, CDCl_3_) *δ* 8.30 (s, 1H), 7.98 (dd, *J* = 8.5, 1.4 Hz, 1H), 7.58 (d, *J* = 8.5 Hz, 1H), 4.42 (q, *J* = 7.1 Hz, 2H), 2.70 (s, 3H), 1.43 (t, *J* = 7.1 Hz, 3H); ^13^C NMR (100 MHz, CDCl_3_) *δ* 167.4, 153.7, 142.4, 138.1, 124.6, 123.9, 114.2, 60.1, 15.1, 14.3. HRMS (ESI): *m*/*z* [M + H]^+^ calcd for C_11_H_13_N_2_O_2_ 205.0972, found: 205.0969.

#### Benzimidazoles 2d

Following the general procedure compound 2d was obtained from 1d (202 mg, 1.0 mmol). The crude product was purified by silica-gel column chromatography (DCM/MeOH = 100/1 to 20/1) to afford the title compound 2d (186 mg, 98%) as a white solid; m.p. 82–84 °C; ^1^H NMR (400 MHz, CDCl_3_) *δ* 7.36 (d, *J* = 8.7 Hz, 1H), 6.97 (d, *J* = 1.3 Hz, 1H), 6.83–6.74 (m, 1H), 3.73 (s, 3H), 2.82 (t, *J* = 7.5 Hz, 2H), 1.86–1.72 (m, 2H), 0.90 (t, *J* = 7.3 Hz, 3H); ^13^C NMR (100 MHz, CDCl_3_) *δ* 156.4, 154.5, 137.9, 132.4, 115.1, 111.8, 97.5, 55.8, 30.9, 21.6, 13.7. HRMS (ESI): *m*/*z* [M + H]^+^ calcd for C_11_H_15_N_2_O 191.1179, found: 191.1177.

#### Benzimidazoles 2e

Following the general procedure compound 2e was obtained from 1e (202 mg, 1.0 mmol). The crude product was purified by silica-gel column chromatography (DCM/MeOH = 100/1 to 20/1) to afford the title compound 2e (186 mg, 98%) as a white solid; m.p. 130–131 °C; ^1^H NMR (400 MHz, CDCl_3_) *δ* 7.22–7.07 (m, 2H), 6.67 (dd, *J* = 7.5, 1.2 Hz, 1H), 3.94 (s, 3H), 2.90 (t, *J* = 7.4 Hz, 2H), 2.03–1.76 (m, 2H), 0.99 (t, *J* = 7.4 Hz, 3H); ^13^C NMR (100 MHz, CDCl_3_) *δ* 154.0, 148.3, 139.7, 128.6, 122.6, 107.4, 102.6, 55.5, 31.1, 21.7, 13.8. HRMS (ESI): *m*/*z* [M + H]^+^ calcd for C_11_H_15_N_2_O 191.1179, found: 191.1176.

#### Benzimidazoles 2f

Following the general procedure compound 2f was obtained from 1f (200 mg, 1.0 mmol). The crude product was purified by silica-gel column chromatography (DCM/MeOH = 100/1 to 20/1) to afford the title compound 2f (178 mg, 95%) as a white solid; m.p. 170–171 °C; ^1^H NMR (400 MHz, CD_3_OD) *δ* 7.34 (s, 1H), 7.20 (s, 1H), 3.16–3.12 (m, 2H), 2.59 (s, 3H), 2.49 (s, 3H), 2.07–1.86 (m, 2H), 1.07 (t, *J* = 7.4 Hz, 3H); ^13^C NMR (100 MHz, CD_3_OD) *δ* 153.3, 136.7, 131.0, 128.6, 127.9, 123.6, 110.1, 27.8, 20.6, 20.1, 15.1, 12.3. HRMS (ESI): *m*/*z* [M + H]^+^ calcd for C_12_H_17_N_2_ 189.1386, found: 189.1383.

#### Benzimidazoles 2g

Following the general procedure compound 2g was obtained from 1g (275 mg, 1.0 mmol). The crude product was purified by silica-gel column chromatography (DCM/MeOH = 100/1 to 20/1) to afford the title compound 2g (247 mg, 94%) as a foamy solid. ^1^H NMR (400 MHz, DMSO-*d*_6_) *δ* 13.12 (s, 1H), 7.52 (s, 1H), 2.84 (t, *J* = 7.5 Hz, 2H), 1.87–1.75 (m, 2H), 0.95 (t, *J* = 7.4 Hz, 3H);

^13^C NMR (100 MHz, CD_3_OD) *δ* 159.6, 132.6, 131.2, 130.7, 128.1, 119.3, 117.7, 29.65, 22.6, 13.9. HRMS (ESI): *m*/*z* [M + H]^+^ calcd for C_10_H_10_Cl_3_N_2_ 262.9904, found: 262.9898.

#### Benzimidazoles 2h

Following the general procedure compound 2h was obtained from 1h (190 mg, 1.0 mmol). The crude product was purified by silica-gel column chromatography (DCM/MeOH = 100/1 to 20/1) to afford the title compound 2h (132 mg, 88%) as a white solid m.p. 194–196 °C; two sets of ^1^HNMR data representing two isomers (3 : 1) were observed as indicative of the presence of tautomerism; ^1^H NMR (400 MHz, DMSO-*d*_6_, major isomer) *δ* 12.47 (s, 1H), 7.24 (d, *J* = 8.0 Hz, 1H), 7.13–7.03 (m, 1H), 6.99–6.84 (m, 1H), 2.49 (s, 3H). ^1^HNMR (400 MHz, DMSO-*d*_6_, minor isomer) *δ* 12.71 (s, 1H), 7.34 (d, *J* = 7.6 Hz, 1H), 7.13–7.03 (m, 1H), 7.01–6.89 (m, 1H), 2.49 (s, 3H); ^13^C NMR (101 MHz, DMSO) *δ* 154.2, 152.3, 151.7, 138.1, 138.0, 132.0, 122.3, 122.2, 107.5, 106.80, 106.6, 15.0; ^19^F NMR (377 MHz, DMSO) *δ* −135.23. HRMS (ESI): *m*/*z* [M + H]^+^ calcd for C_8_H_8_FN_2_ 151.0666, found: 151.0662.

#### Benzimidazoles 2i

Following the general procedure compound 2i was obtained from 1i (222 mg, 1.0 mmol). The crude product was purified by silica-gel column chromatography (DCM/MeOH = 100/1 to 20/1) to afford the title compound 2i (193 mg, 92%) as a white solid; m.p. 90–93 °C; ^1^H NMR (400 MHz, CD_3_OD) *δ* 8.36–8.28 (m, 1H), 8.09–8.01 (m, 1H), 7.97–7.89 (m, 1H), 7.78–7.67 (m, 2H), 7.67–7.60 (m, 1H), 3.22 (t, *J* = 7.7 Hz, 2H), 2.08–1.95 (m, 2H), 1.11 (t, *J* = 7.3 Hz, 3H); ^13^C NMR (100 MHz, CD_3_OD) *δ* 153.1, 132.6, 132.6, 130.3, 129.3, 129.2, 128.6, 127.9, 122.0, 121.9, 113.1, 29.2, 22.1, 13.8. HRMS (ESI): *m*/*z* [M + H]^+^ calcd for C_14_H_15_N_2_ 211.1230, found: 211.1226.

#### Benzimidazoles 2j

Following the general procedure compound 2j was obtained from 1j (172 mg, 1.0 mmol). The crude product was purified by silica-gel column chromatography (DCM/MeOH = 100/1 to 20/1) to afford the title compound 2j (119 mg, 90%) as a light yellow solid; ^1^H NMR (400 MHz, DMSO-*d*_6_) *δ* 12.15 (s, 1H), 7.43 (d, *J* = 3.2 Hz, 2H), 7.09 (dd, *J* = 5.9, 3.1 Hz, 2H), 2.56–2.21 (m, 3H); ^13^C NMR (101 MHz, DMSO) *δ* 151.6, 121.4, 40.6, 40.4, 15.1. HRMS (ESI): *m*/*z* [M + H]^+^ calcd for C_8_H_9_N_2_ 133.0760.

#### Benzimidazoles 2k

Following the general procedure compound 2k was obtained from 1k (244 mg, 1.0 mmol). The crude product was purified by silica-gel column chromatography (DCM/MeOH = 100/1 to 20/1) to afford the title compound 2k (200 mg, 86%) as a foamy solid; ^1^H NMR (400 MHz, CDCl_3_) *δ* 8.41 (d, *J* = 0.7 Hz, 1H), 8.26 (dd, *J* = 8.6, 1.4 Hz, 1H), 7.86 (d, *J* = 8.6 Hz, 1H), 4.46 (q, *J* = 7.1 Hz, 2H), 3.59 (dt, *J* = 14.0, 7.0 Hz, 1H), 1.60 (d, *J* = 7.0 Hz, 6H), 1.45 (t, *J* = 7.1 Hz, 3H); ^13^C{^1^H} NMR (100 MHz, CD_3_OD) *δ* 166.8, 162.5, 135.2, 132.1, 129.9, 128.2, 116.5, 114.8, 62.8, 29.1, 20.3, 14.6. HRMS (ESI): *m*/*z* [M + H]^+^ calcd for C_13_H_17_N_2_O_2_ 233.1285, found: 233.1282.

#### Benzimidazoles 2l

Following the general procedure compound 2l was obtained from 1l (217 mg, 1.0 mmol). The crude product was purified by silica-gel column chromatography (DCM/MeOH = 100/1 to 20/1) to afford the title compound 2l (229 mg, 77%) as a light yellow solid; m.p. 154–156 °C; ^1^H NMR (400 MHz, ^1^H NMR (400 MHz, DMSO-*d*_6_)) *δ* 9.76–9.38 (s, 1H), 8.03–7.94 (m, 2H), 7.71–7.67 (m, 1H), 7.53–7.51 (s, 1H), 7.31–7.24 (m, 1H), 7.03–6.97 (dd, *J* = 8.1, 1.0 Hz, 1H), 2.24–1.93 (s, 3H); ^13^C{^1^H} NMR (100 MHz, DMSO-*d*_6_) *δ* 167.9, 152.8, 150.4, 142.1, 135., 134.5, 134.4, 133.4, 133.4, 128.1, 127.1, 125.8, 125.2, 125.1, 19.7. HRMS (ESI): *m*/*z* [M + H]^+^ calcd for C_14_H_14_N_4_O_4_ 299.0775, found: 299.0772.

#### Benzimidazoles 2m

Following the general procedure compound 2m was obtained from 1m (217 mg, 1.0 mmol). The crude product was purified by silica-gel column chromatography (DCM/MeOH = 100/1 to 20/1) to afford the title compound 2m (133 mg, 65%) as a light yellow solid; m.p. 160–162 °C; ^1^H NMR (400 MHz, CD_3_OD) *δ* 8.66 (d, *J* = 1.9 Hz, 1H), 8.46 (dd, *J* = 9.0, 2.1 Hz, 1H), 7.97 (d, *J* = 9.0 Hz, 1H), 3.28–3.20 (m, 2H), 2.07–1.95 (m, 2H), 1.12 (t, *J* = 7.4 Hz, 3H); ^13^C{^1^H} NMR (100 MHz, CD_3_OD) *δ* 158.8, 145.8, 134.8, 130.7, 121.1, 114.3, 110.1, 28.3, 20.1, 12.3. HRMS (ESI): *m*/*z* [M + H]^+^ calcd for C_10_H_12_N_3_O_2_ 206.0924, found: 206.0921.

#### Benzimidazoles 2n

Following the general procedure compound 2n was obtained from 1n (197 mg, 1.0 mmol). The crude product was purified by silica-gel column chromatography (DCM/MeOH = 100/1 to 20/1) to afford the title compound 2n (98 mg, 53%) as a light yellow solid; m.p. 168–169 °C. ^1^H NMR (400 MHz, CD_3_OD) *δ* 8.12 (d, *J* = 8.3 Hz, 1H), 8.02 (d, *J* = 7.7 Hz, 1H), 7.75 (t, *J* = 8.0 Hz, 1H), 3.24 (t, *J* = 7.7 Hz, 2H), 2.12–1.93 (m, 2H), 1.13 (t, *J* = 7.4 Hz, 3H); ^13^C{^1^H} NMR (100 MHz, CD_3_OD) *δ* 157.0, 131.8, 131.6, 130.5, 126.1, 118.7, 113.9, 97.9, 28.1, 20.4, 12.4. HRMS (ESI): *m*/*z* [M + H]^+^ calcd for C_11_H_12_N_3_ 186.1026, found: 186.1024.

#### Benzimidazoles 2o

Following the general procedure compound 2o was obtained from 1o (244 mg, 1.0 mmol). The crude product was purified by silica-gel column chromatography (DCM/MeOH = 100/1 to 50/1) to afford compound 2o (220 mg, 95%) as a foamy solid; For compound 2o^1^H NMR (400 MHz, CD_3_OD) *δ* 8.22 (d, *J* = 7.7 Hz, 1H), 8.04 (d, *J* = 8.1 Hz, 1H), 7.76–7.68 (m, 1H), 4.57 (q, *J* = 7.1 Hz, 1H), 3.72 (dd, *J* = 14.0, 7.0 Hz, 1H), 1.59 (d, *J* = 7.0 Hz, 1H), 1.48 (t, *J* = 7.1 Hz, 1H); ^13^C{^1^H} NMR (100 MHz, CD_3_OD) *δ* 165.8, 162.5, 134.8, 131.8, 128.3, 126.4, 120.4, 117.9, 62.9, 28.8, 21.0, 14.7. HRMS (ESI): *m*/*z* [M + H]^+^ calcd for C_13_H_17_N_2_O_2_ 233.1285, found: 233.1283.

#### Benzimidazoles 2p

Following the general procedure compound 2p was obtained from 1p (172 mg, 1.0 mmol). The crude product was purified by silica-gel column chromatography (DCM/MeOH = 100/1 to 20/1) to afford the title compound 2p (244 mg, 91%) as a foamy solid; ^1^H NMR (400 MHz, chloroform-*d*) *δ* 7.52 (s, 2H), 7.21 (dd, *J* = 6.0, 3.2 Hz, 2H), 6.44 (d, *J* = 2.1 Hz, 2H), 6.37 (d, *J* = 2.1 Hz, 1H), 4.21 (s, 6H), 3.73 (s, 3H); ^13^C NMR (101 MHz, CDCl_3_) *δ* 161.3, 153.0, 138.3, 122.4, 107.1, 99.2, 55.3, 36.2. HRMS (ESI): *m*/*z* [M + H]^+^ calcd for C_16_H_17_N_2_O_2_ 269.1285.

#### Benzimidazoles 2q

Following the general procedure compound 2q was obtained from 1q (173 mg, 1.0 mmol). The crude product was purified by silica-gel column chromatography (DCM/MeOH = 100/1 to 20/1) to afford the title compound 2q (140 mg, 88%) as a white solid; ^1^H NMR (400 MHz, Methanol-*d*_4_) *δ* 8.24 (d, *J* = 4.4 Hz, 1H), 7.83 (dd, *J* = 8.0, 1.3 Hz, 1H), 7.21 (dd, *J* = 8.0, 4.9 Hz, 1H), 2.19 (s, 0H), 1.43–1.03 (m, 4H); ^13^C NMR (101 MHz, CD_3_OD) *δ* 160.4, 151.6, 142.4, 130.7, 121.8, 117.5, 9.2, 8.4. HRMS (ESI): *m*/*z* [M + H]^+^ calcd for C_9_H_10_N_3_ 160.0869.

#### Benzimidazoles 2r

Following the general procedure compound 2r was obtained from 1r (173 mg, 1.0 mmol). The crude product was purified by silica-gel column chromatography (DCM/MeOH = 100/1 to 20/1) to afford the title compound 2r (150 mg, 93%) as a light yellow solid; ^1^H NMR (400 MHz, Methanol-*d*_4_) *δ* 8.62 (dd, *J* = 5.2, 1.2 Hz, 1H), 8.33 (dd, *J* = 8.2, 1.3 Hz, 1H), 7.66 (dd, *J* = 8.2, 5.2 Hz, 1H), 3.16 (t, *J* = 7.6 Hz, 2H), 1.98 (q, *J* = 7.5 Hz, 2H), 1.09 (t, *J* = 7.4 Hz, 3H); ^13^C NMR (101 MHz, CD_3_OD) *δ* 164.7, 145.0, 135.1, 134.6, 131.5, 111.2, 29.8, 20.4, 12.5. HRMS (ESI): *m*/*z* [M + H]^+^ calcd for C_9_H_12_N_3_ 162.1026.

#### Benzimidazoles 2s

Following the general procedure, compound 2s and was obtained from 1s (173 mg, 1.0 mmol). The crude product was purified by silica-gel column chromatography (DCM/MeOH = 80/1 to 50/1) to afford compound 2s (153 mg, 95%) as a foamy solid; ^1^H NMR (400 MHz, CD_3_OD) *δ* 8.62 (dd, *J* = 5.2, 1.2 Hz, 1H), 8.33 (dd, *J* = 8.2, 1.3 Hz, 1H), 7.66 (dd, *J* = 8.2, 5.2 Hz, 1H), 3.16 (t, *J* = 7.6 Hz, 2H), 1.98 (q, *J* = 7.5 Hz, 2H), 1.09 (t, *J* = 7.4 Hz, 3H); ^13^C NMR (101 MHz, CD_3_OD) *δ* 160.5, 147.0, 144.8, 127.5, 125.6, 121.8, 30.4, 21.7, 13.8. HRMS (ESI): *m*/*z* [M + H]^+^ calcd for C_9_H_11_N_3_ 235.1077, found: 162.1023.

#### Benzimidazoles 2t

Following the general procedure compound 2t was obtained from 1t (173 mg, 1.0 mmol). The crude product was purified by silica-gel column chromatography (DCM/MeOH = 100/1 to 20/1) to afford the title compound 2t (234 mg, 87%) as a white solid; ^1^H NMR (400 MHz, methanol-*d*_4_) *δ* 8.30 (s, 1H), 7.91 (s, 1H), 7.25 (dd, *J* = 7.9, 4.9 Hz, 1H), 6.51 (d, *J* = 1.7 Hz, 2H), 6.37 (s, 1H), 4.86 (s, 3H), 4.19 (s, 2H), 3.74 (s, 6H), 3.31 (s, 3H); ^13^C NMR (101 MHz, CD_3_OD) *δ* 162.7, 144.5, 139.6, 119.4, 107.9, 100.0, 55.7, 36.6. HRMS (ESI): *m*/*z* [M + H]^+^ calcd for C_15_H_16_N_3_O_2_ 270.1237.

#### Benzimidazoles 2u

Following the general procedure compound 2u was obtained from 1u (172 mg, 1.0 mmol). The crude product was purified by silica-gel column chromatography (DCM/MeOH = 100/1 to 20/1) to afford the title compound 2u (213 mg, 95%) as a light yellow solid; ^1^H NMR (400 MHz, methanol-*d*_4_) *δ* 8.24 (dd, *J* = 7.8, 1.7 Hz, 1H), 7.64 (dd, *J* = 6.0, 3.2 Hz, 2H), 7.47 (ddd, *J* = 8.7, 7.5, 1.7 Hz, 1H), 7.24 (dd, *J* = 6.1, 3.2 Hz, 2H), 7.19 (d, *J* = 8.3 Hz, 1H), 7.15–7.08 (m, 1H), 4.05 (s, 3H); ^13^C NMR (101 MHz, CD_3_OD) *δ* 157.3, 149.6, 138.1, 131.4, 129.5, 122.2, 120.7, 117.5, 114.4, 111.4, 54.8. HRMS (ESI): *m*/*z* [M + H]^+^ calcd for C_14_H_13_N_2_O 225.1022.

#### Benzimidazoles 2v

Following the general procedure compound 2v was obtained from 1v (172 mg, 1.0 mmol). The crude product was purified by silica-gel column chromatography (DCM/MeOH = 100/1 to 30/1) to give 2v (186 mg, 93%) as a yellow solid; m.p. 168–169 °C; ^1^H NMR (400 MHz, methanol-*d*_4_) *δ* 8.20 (dd, *J* = 3.9, 1.1 Hz, 1H), 8.11 (dd, *J* = 5.0, 1.1 Hz, 1H), 7.78 (dt, *J* = 6.8, 3.4 Hz, 2H), 7.60 (dt, *J* = 6.2, 3.4 Hz, 2H), 7.43 (dd, *J* = 4.9, 3.9 Hz, 1H); ^13^C NMR (101 MHz, CD_3_OD) *δ* 145.8, 135.5, 134.5, 132.7, 130.6, 127.8, 124.9, 114.7. HRMS (ESI): *m*/*z* [M + H]^+^ calcd for C_11_H_9_S_2_ 201.0481.

#### Benzimidazoles 2w

Following the general procedure, compound 2w and was obtained from 1w (172 mg, 1.0 mmol). The crude product was purified by silica-gel column chromatography (DCM/MeOH = 100/1 to 30/1) to give 2w (190 mg, 75%) as a white solid; m.p. 221–223 °C; ^1^H NMR (400 MHz, DMSO-*d*_6_) *δ* 13.33 (s, 1H), 8.31 (s, 1H), 8.25–8.16 (m, 2H), 7.66 (dd, *J* = 5.9, 3.2 Hz, 2H), 7.32–7.23 (m, 2H), 2.64 (s, 3H); ^13^C{^1^H} NMR (100 MHz, DMSO-*d*_6_) *δ* 149.4, 149.3, 139.9, 134.7, 134.1, 130.8, 130.7, 125.8, 125.3, 123.2, 115.9, 20.3. HRMS (ESI) *m*/*z* [M + H]^+^ calcd for C_14_H_12_N_3_O_2_ 254.0924, found 254.0919.

#### Benzimidazoles 2x

Following the general procedure compound 2x was obtained from 1x (172 mg, 1.0 mmol). The crude product was purified by silica-gel column chromatography (DCM/MeOH = 100/1 to 30/1) to give 2x (198 mg, 85%) as a yellow solid; ^1^H NMR (400 MHz, DMSO-*d*_6_) *δ* 12.40 (s, 1H), 7.81–7.66 (m, 3H), 7.47 (s, 2H), 7.10 (dd, *J* = 5.9, 3.2 Hz, 2H), 6.69 (d, *J* = 8.3 Hz, 1H), 5.35 (s, 2H), 2.14 (s, 3H); ^13^C NMR (101 MHz, DMSO) *δ* 154.7, 148.7, 143.1, 137.1, 131.6, 128.3, 121.9, 121.9, 121.2, 118.77, 117.6, 113.8, 110.4, 32.2, 17.92. HRMS (ESI): *m*/*z* [M + H]^+^ calcd for C_14_H_14_N_3_ 224.1128.

#### Benzimidazoles 2y

Following the general procedure compound 2y was obtained from 1y (172 mg, 1.0 mmol). The crude product was purified by silica-gel column chromatography (DCM/MeOH = 100/1 to 30/1) to give 2y (186 mg, 96%) as a yellow solid; ^1^H NMR (400 MHz, DMSO-*d*_6_) *δ* 12.90 (s, 1H), 8.30–8.03 (m, 2H), 7.67–7.45 (m, 5H), 7.33–6.96 (m, 2H); ^13^C NMR (101 MHz, DMSO) *δ* 151.6, 130.6, 130.3, 129.4, 126.8, 122.5. HRMS (ESI): *m*/*z* [M + H]^+^ calcd for C_13_H_11_N_2_ 195.0917.

#### Benzimidazoles 2z

Following the general procedure compound 2z was obtained from 1z (172 mg, 2.0 mmol). The crude product was purified by silica-gel column chromatography (DCM/MeOH = 100/1 to 30/1) to give 2z (204 mg, 78%) as a yellow solid; ^1^H NMR (400 MHz, Methanol-*d*_4_) *δ* 8.36 (d, *J* = 8.2 Hz, 2H), 8.08 (d, *J* = 8.3 Hz, 2H), 7.90 (dd, *J* = 6.2, 3.2 Hz, 2H), 7.68 (dd, *J* = 6.2, 3.1 Hz, 2H); ^13^C NMR (101 MHz, CD_3_OD) *δ* 149.3, 135.9, 135.6, 133.2, 130.1, 128.3, 128.1, 128.1, 128.0, 127.9, 127.9, 126.2, 123.5, 115.2; ^19^F NMR (377 MHz, CD_3_OD) *δ* −63.13, −63.61. HRMS (ESI): *m*/*z* [M + H]^+^ calcd for C_14_H_10_F_3_N_2_ 263.0791.

#### Benzimidazoles 2aa

Following the general procedure compound 2aa was obtained from 1aa (172 mg, 1.0 mmol). The crude product was purified by silica-gel column chromatography (DCM/MeOH = 100/1 to 30/1) to give 2aa (259 mg, 95%) as a yellow solid; ^1^H NMR (400 MHz, DMSO-*d*_6_) *δ* 13.33–12.82 (s, 1H), 8.42–8.33 (t, *J* = 1.7 Hz, 1H), 8.22–8.14 (dt, *J* = 7.8, 1.1 Hz, 1H), 7.74–7.68 (ddd, *J* = 8.0, 1.9, 0.9 Hz, 1H), 7.66–7.57 (s, 2H), 7.57–7.47 (t, *J* = 7.9 Hz, 1H), 7.30–7.15 (m, 2H); ^13^C NMR (101 MHz, DMSO) *δ* 150.1, 132.9, 132.8, 131.6, 129.3, 125.8, 122.7. HRMS (ESI): *m*/*z* [M + H]^+^ calcd for C_13_H_10_BrN_2_ 273.0022.

#### Benzimidazoles 2bb

Following the general procedure compound 2bb was obtained from 1bb (172 mg, 1.0 mmol). The crude product was purified by silica-gel column chromatography (DCM/MeOH = 100/1 to 30/1) to give 2bb (187 mg, 96%) as a yellow solid; ^1^H NMR (400 MHz, Methanol-*d*_4_) *δ* 8.69 (d, *J* = 5.0 Hz, 2H), 8.12–7.96 (m, 2H), 7.64 (dd, *J* = 5.9, 3.2 Hz, 2H), 7.40–7.22 (m, 2H); ^13^C NMR (101 MHz, CD_3_OD) *δ* 151.1, 150.0, 139.2, 124.8, 122.1, 116.6, 112.7. HRMS (ESI): *m*/*z* [M + H]^+^ calcd for C_12_H_10_N_3_ 196.0896.

#### Benzimidazoles 2cc

Following the general procedure compound 2cc was obtained from 1cc (172 mg, 1.0 mmol). The crude product was purified by silica-gel column chromatography (DCM/MeOH = 100/1 to 30/1) to give 2cc (147 mg, 93%) as a yellow solid; ^1^H NMR (400 MHz, Chloroform-*d*) *δ* 7.51 (dd, *J* = 6.0, 3.2 Hz, 2H), 7.19 (dd, *J* = 6.0, 3.2 Hz, 2H), 2.16–1.99 (m, 1H), 1.35–1.19 (m, 2H), 1.18–0.97 (m, 2H); ^13^C NMR (101 MHz, CDCl_3_) *δ* 156.5, 135.5, 122.2, 114.3, 9.6, 8.8, −0.01. HRMS (ESI): *m*/*z* [M + H]^+^ calcd for C_10_H_11_N_2_ 159.0917.

##### Synthesis of bendazol

The reaction mixture, consisting of 1 (1 eq.), potassium-2-methylbutan-2-olate (2 eq.), and 2-phenylacetonitrile 3 (2 eq.) in the presence of CuI (0.1 eq.), was vigorously stirred in a reaction vessel for 17 hours at 110–120 °C. Upon completion, the reaction was cooled to 25 °C and quenched with water. The resulting mixture was then extracted with ethyl acetate, and the organic layer was washed with brine, dried over Na_2_SO_4_, filtered, and concentrated *in vacuo*. The crude product was subsequently purified by re-crystallization from DCM/hexane, yielding bendazol as a light yellow solid (80.9 g, 82%). ^1^H NMR (400 MHz, DMSO-*d*_6_) *δ* 15.53 (s, 1H), 7.77 (dt, *J* = 6.7, 3.4 Hz, 2H), 7.62–7.46 (m, 4H), 7.44–6.86 (m, 3H), 4.57 (s, 2H); ^13^C NMR (101 MHz, DMSO) *δ* 152.5, 134.0, 130.7, 129.1, 128.9, 127.6, 125.5, 113.8, 31.8.

##### Synthesis of thiabendazole

Following the general procedure, this material was obtained as white solid in a yield 93.2 g (78%). ^1^H NMR (400 MHz, DMSO-*d*_6_) *δ* 13.00 (s, 1H), 9.34 (d, *J* = 2.0 Hz, 1H), 8.47 (d, *J* = 1.9 Hz, 1H), 7.61 (dd, *J* = 55.4, 7.1 Hz, 2H), 7.23 (dt, *J* = 8.6, 4.9 Hz, 2H); ^13^C NMR (101 MHz, DMSO) *δ* 155.5, 147.0, 146.9, 143.7, 134.3, 122.5, 121.7, 119.3, 118.7, 111.7.

## Conflicts of interest

The authors declare that they have no apparent conflicting financial interests or personal connections that could have appeared to impact the findings presented in this manuscript.

## Supplementary Material

RA-014-D4RA00245H-s001
