# Supplementary material for: Improved and ligand-free copper-catalyzed cyclization for an efficient synthesis of benzimidazoles from o-bromoarylamine and nitriles
Source: RSC Adv. 2024 Feb 26;14(10):6906–16. doi: 10.1039/d4ra00245h (PMC10895414; doi:10.1039/d4ra00245h)
Supplement: RA-014-D4RA00245H-s001 [file RA-014-D4RA00245H-s001.pdf]

Electronic Supplementary Material (ESI) for RSC Advances.
This journal is © The Royal Society of Chemistry 2024

Supporting Information

Improved and Ligand-free Copper-Catalyzed Cyclization for an Efficient Synthesis of Benzimidazoles from *O*-Bromoarylamine and Nitriles

Emmanuel Mintah Bonku,[†,‡,§] Hongjian Qin,[†,‡,⁑,§] Abdullajon Odilov,[†,‡] Safomuddin Abduahadi,[†,‡] Samuel Desta Guma,[†,‡] Feipu Yang,[†] Fuqiang Zhu,[ɸ] Haji A. Aisa,[‡,⁑,*] Jingshan Shen,[†,‡,*]

[†]State Key Laboratory of Drug Research, Shanghai Institute of Materia Medica, Chinese Academy of Sciences, 555 Zuchongzhi Road, Shanghai 201203, P. R. China

[‡]University of Chinese Academy of Sciences, No. 19A Yuquan Road, Beijing 100049, P. R. China

[⁑]State Key Laboratory Basis of Xinjiang Indigenous Medicinal Plants Resource Utilization, Xinjiang Technical Institute of Physics and Chemistry, Chinese Academy of Sciences, Urumqi, Xinjiang 830011, P. R. China

[ɸ]Topharman Shanghai Co., Ltd., No.388 Jialilue Road, Zhangjiang Hitech Park, Shanghai 201203 P.R. China

*Corresponding authors:

*E-mail: shenjingshan@simm.ac.cn

*E-mail: haji@ms.xjb.ac.cn

[§]These authors contributed equally to this work.

Context

Analytical Data of Related Compounds

Benzimidazoles 2

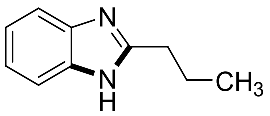

^{1}H NMR (500 MHz, $CDCl_3$) δ 7.92 (brs, 1H), 7.56 (dd, J = 6.0, 3.2 Hz, 2H), 7.35 – 7.16 (m, 2H), 2.93 (t, J = 7.6 Hz, 2H), 1.95 – 1.86 (m, 2H), 1.01 (t, J = 7.4 Hz, 3H)

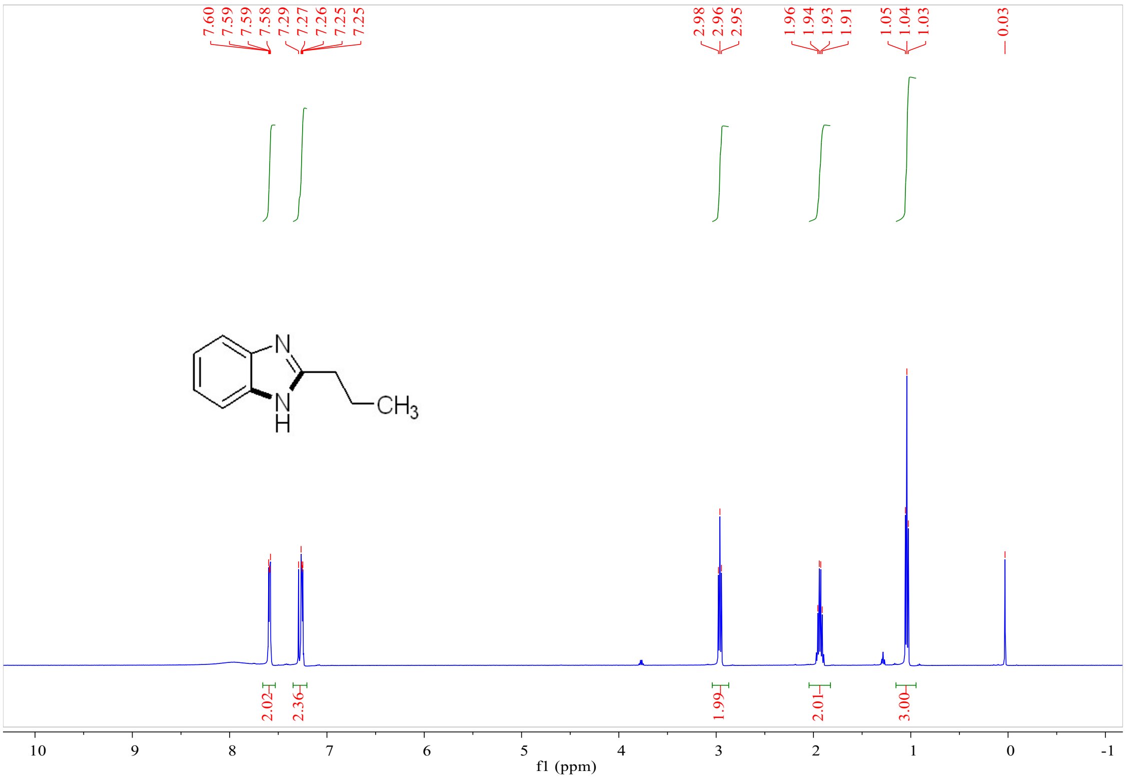

Figure S1 ^{1}HNMR of compound **2** in $CDCl_3$

^{13}C NMR (125 MHz, $CDCl_3$) δ 155.6, 138.5, 122.1, 114.6, 31.2, 21.8, 13.8; HRMS (ESI) m/z [M + H]$^+$ calcd for $C_{10}H_{13}N_2$ 161.1073, found 161.1071.

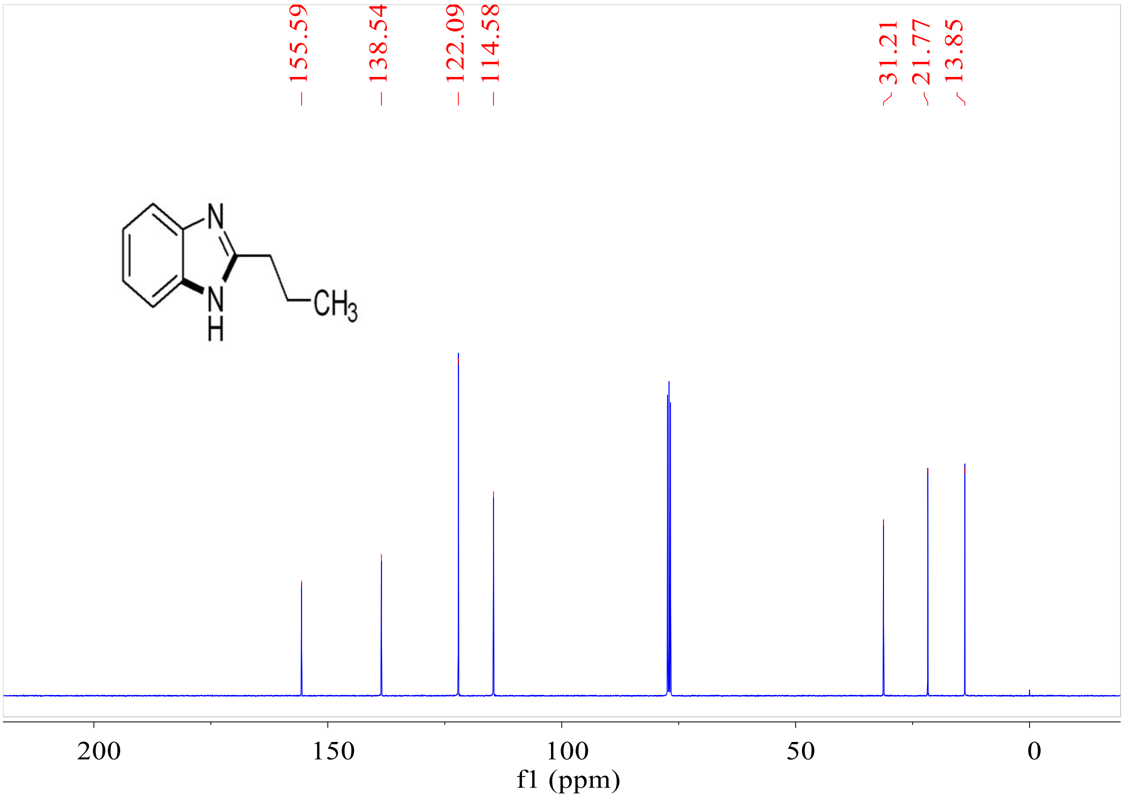

Figure S2 ^{13}C NMR of compound **2** in $CDCl_3$

Benzimidazoles 2a

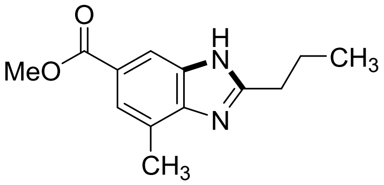

^{1}H NMR (400 MHz, $CDCl_3$) δ 8.87 (brs, 1H), 8.11 (s, 1H), 7.77 (s, 1H), 3.91 (s, 3H), 2.93 (t, J = 7.6 Hz, 2H), 2.57 (s, 3H), 1.94 – 1.83 (m, 2H), 0.96 (t, J = 7.4 Hz, 3H)

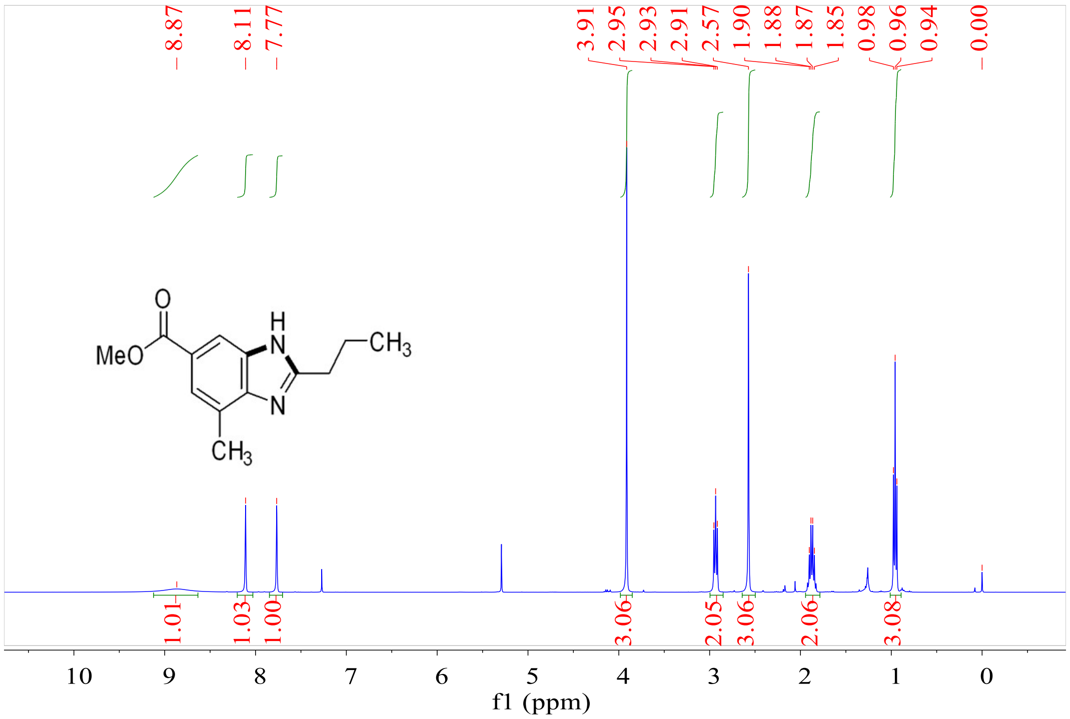

Figure S3 ^{1}H NMR of compound **2a** in $CDCl_3$

^{13}C NMR (100 MHz, $CDCl_3$) δ 168.1, 157.3, 141.8, 137.5, 124.6, 124.2, 124.1, 114.3, 52.1, 31.3, 21.8, 17.1, 13.8; HRMS (ESI): m/z $[M + H]^+$ calcd for $C_{13}H_{17}N_2O_2$ 233.1285, found: 233.1281.

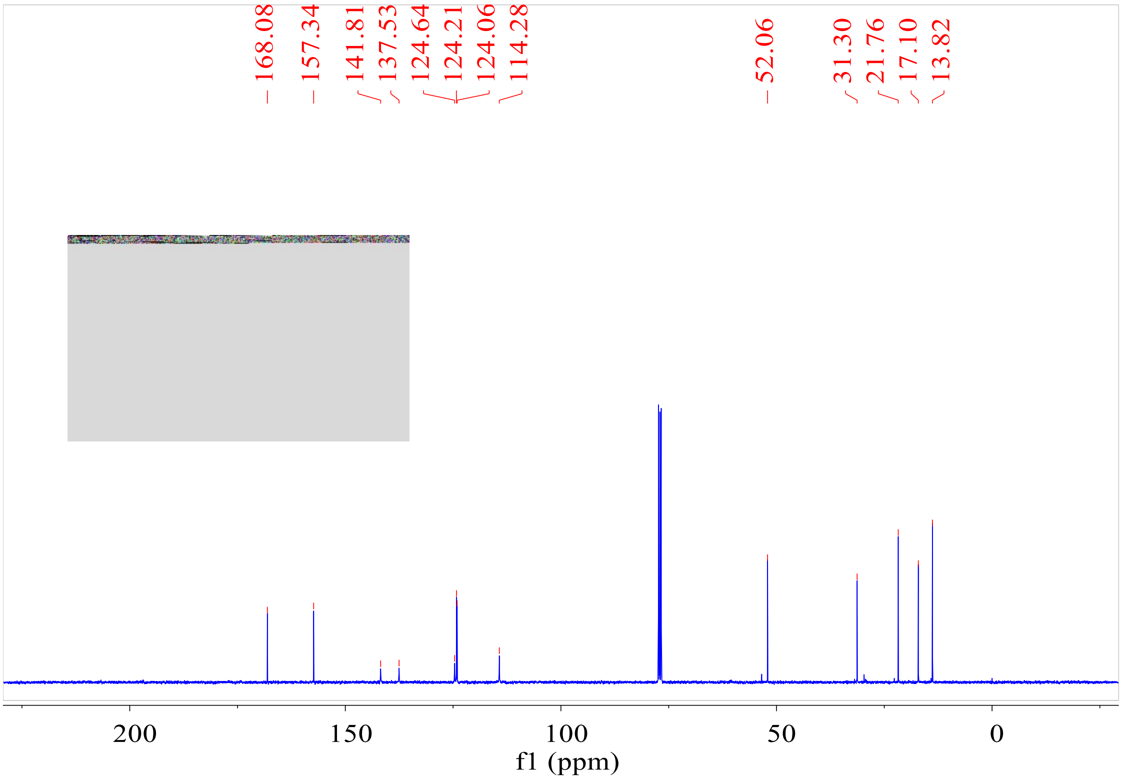

Figure S4 ^{13}C NMR of compound **2a** in $CDCl_3$

Benzimidazoles 2b

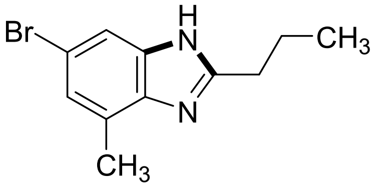

Two sets of ^{1}HNMR data representing two isomers (10:9) were observed as indicative of the presence of tautomerism; ^{1}H NMR (400 MHz, DMSO-d_6, major isomer) δ 12.30 (s, 1 H), 7.51 (s, 1 H), 7.08 (s, 1H), 2.77 (t, J = 7.1 Hz, 2H), 2.45 (s, 3 H), 1.78 (dt, J = 14.3, 7.2 Hz, 2H), 0.94 (t, J = 7.4 Hz, 3H); HNMR (400 MHz, DMSO-d_6, minor isomer) δ 12.24 (s, 1 H), 7.40 (s, 1H), 7.08 (s, 1H), 2.77 (t, J = 7.1 Hz, 2H), 2.48 (s, 3H), 1.78 (dt, J = 14.3, 7.2 Hz, 2H), 0.94 (t, J = 7.4 Hz, 3H)

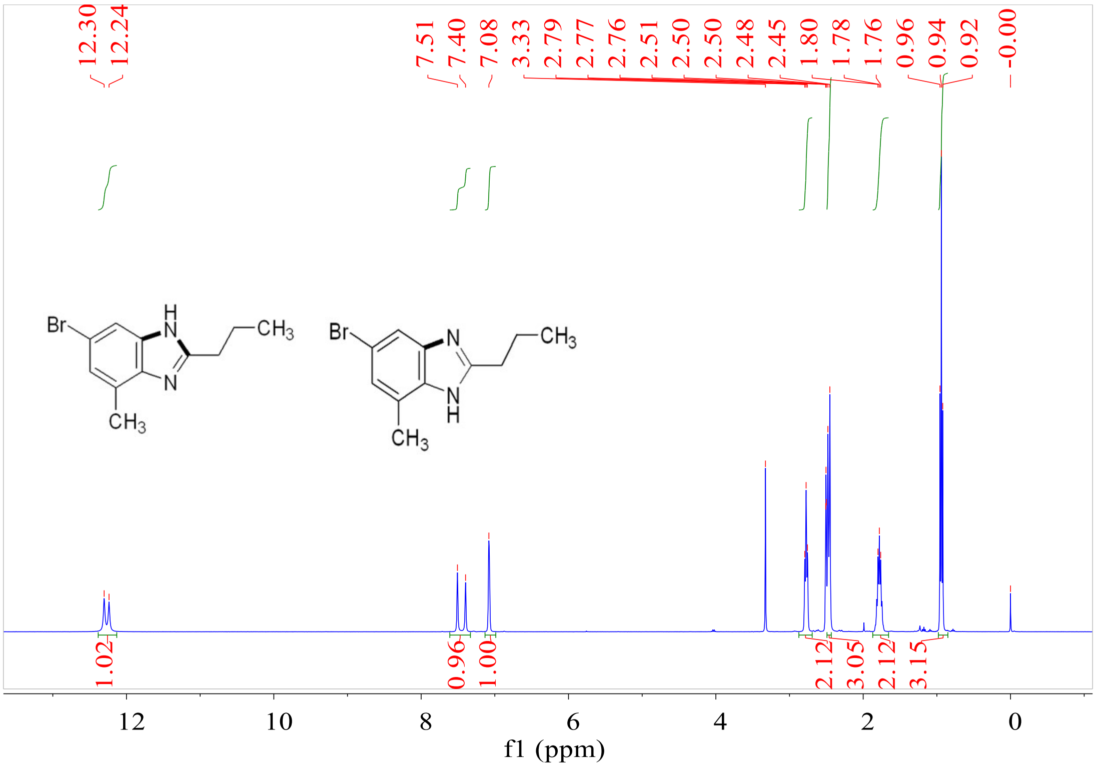

Figure S5 ^{1}H NMR of compound **2b** in DMSO-d_6

^{13}C NMR (101 MHz, DMSO-d_6, minor isomer) δ 156.8, 155.6, 144.9, 142.3, 135.4, 133.6, 130.3, 124.9, 124.2, 123.2, 118.4, 113.8, 113.4, 111.3, 30.1, 30.9, 21.4, 17.1, 16.7, 14.1. HRMS (ESI): m/z [M + H]$^+$ calcd for $C_{11}H_{14}BrN_2$ 253.0335, found: 253.0332.

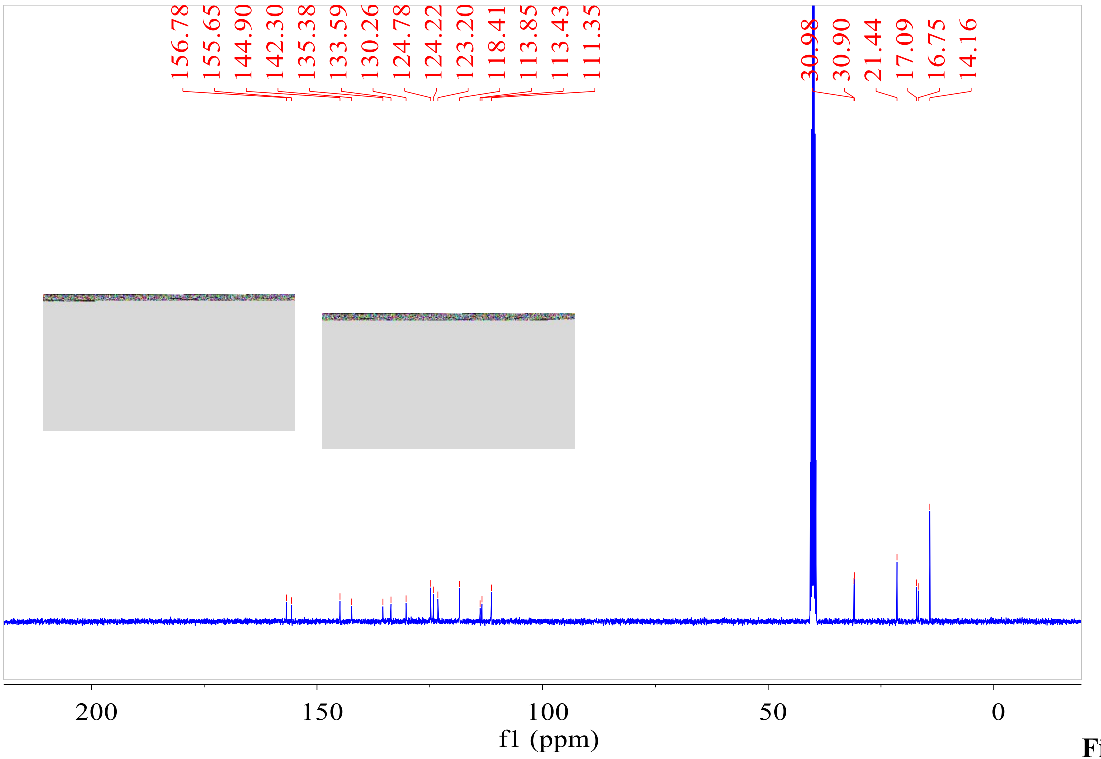

Fi

gure S6 ^{13}C NMR of compound **2b** in DMSO-d_6

Benzimidazoles 2c

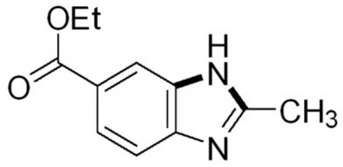

^{1}H NMR (400 MHz, $CDCl_3$) δ 8.30 (s, 1H), 7.98 (dd, J = 8.5, 1.4 Hz, 1H), 7.58 (d, J = 8.5 Hz, 1H), 4.42 (q, J = 7.1 Hz, 2H), 2.70 (s, 3H), 1.43 (t, J = 7.1 Hz, 3H).

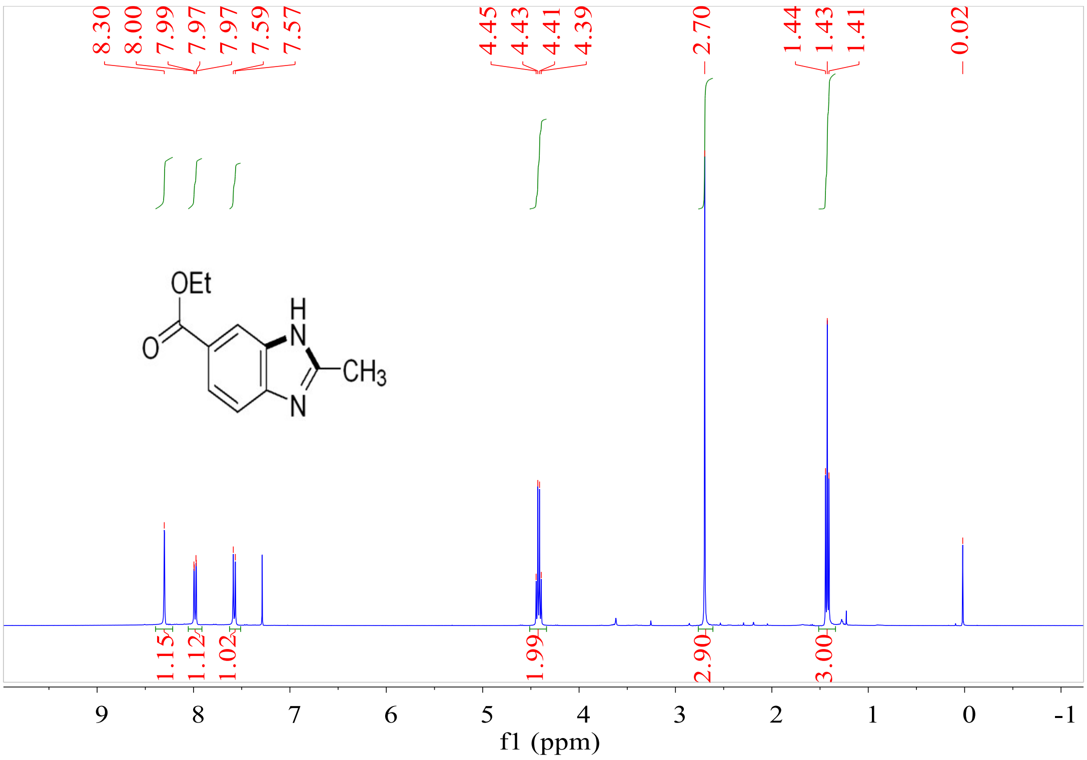

Figure S7 ^{1}H NMR of compound **2c** in $CDCl_3$

^{13}C NMR (100 MHz, $CDCl_3$) δ 167.4, 153.7, 142.4, 138.1, 124.6, 123.9, 114.2, 60.1, 15.1, 14.3.

HRMS (ESI): m/z $[M + H]^+$ calcd for $C_{11}H_{13}N_2O_2$ 205.0972, found: 205.0969.

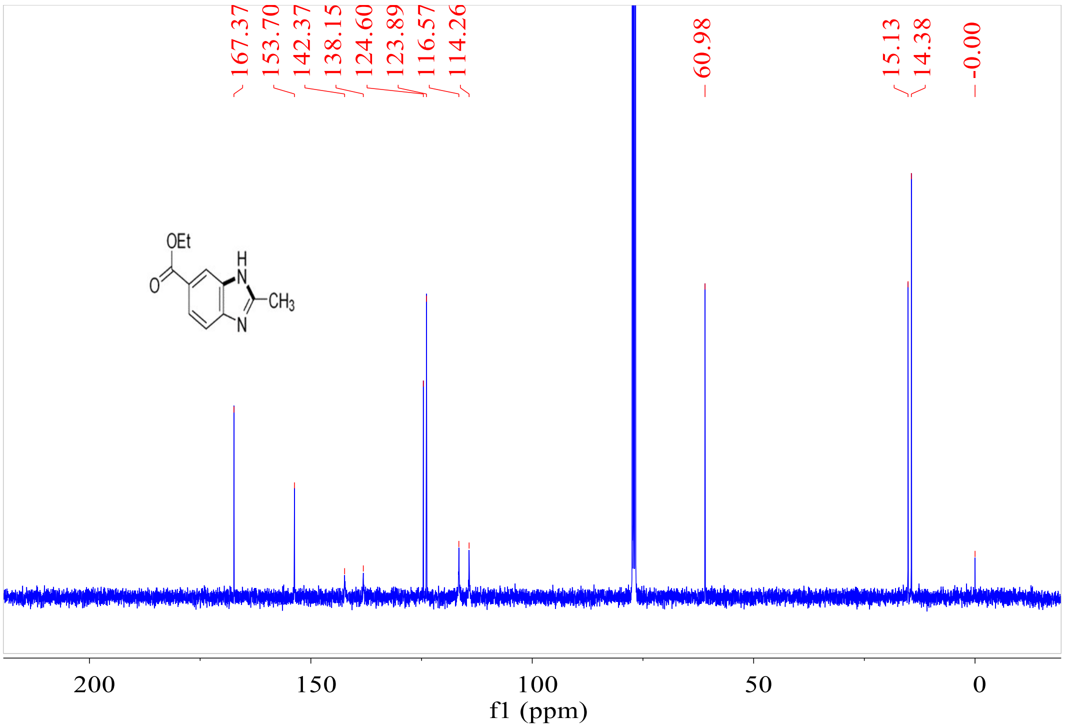

Figure S8 ^{13}C NMR of compound **2c** in $CDCl_3$

Benzimidazoles 2d

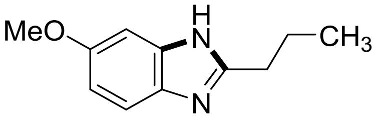

^{1}H NMR (400 MHz, $CDCl_3$) δ 7.36 (d, J = 8.7 Hz, 1H), 6.97 (d, J = 1.3 Hz, 1H), 6.83 – 6.74 (m, 1H), 3.73 (s, 3H), 2.82 (t, J = 7.5 Hz, 2H), 1.86 – 1.72 (m, 2H), 0.90 (t, J = 7.3 Hz, 3H)

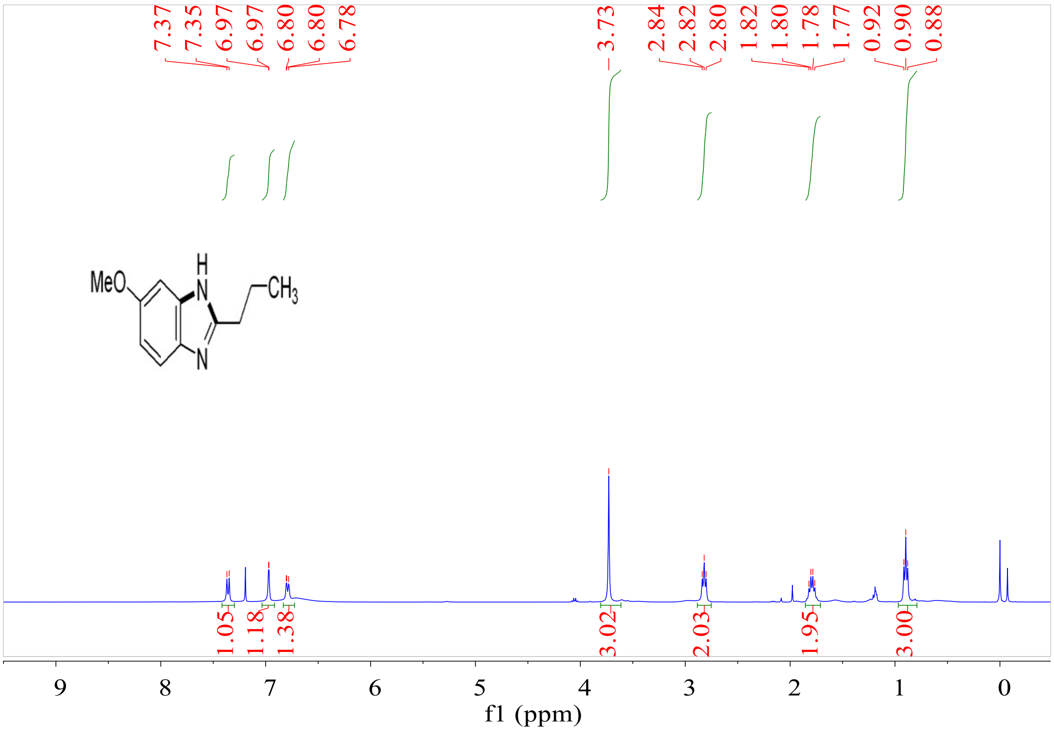

Figure S9 ^{1}H NMR of compound **2d** in $CDCl_3$

^{13}C NMR (100 MHz, $CDCl_3$) δ 156.4, 154.5, 137.9, 132.4, 115.1, 111.8, 97.5, 55.8, 30.9, 21.6, 13.7;

HRMS (ESI): m/z $[M + H]^+$ calcd for $C_{11}H_{15}N_2O$ 191.1179, found: 191.1177.

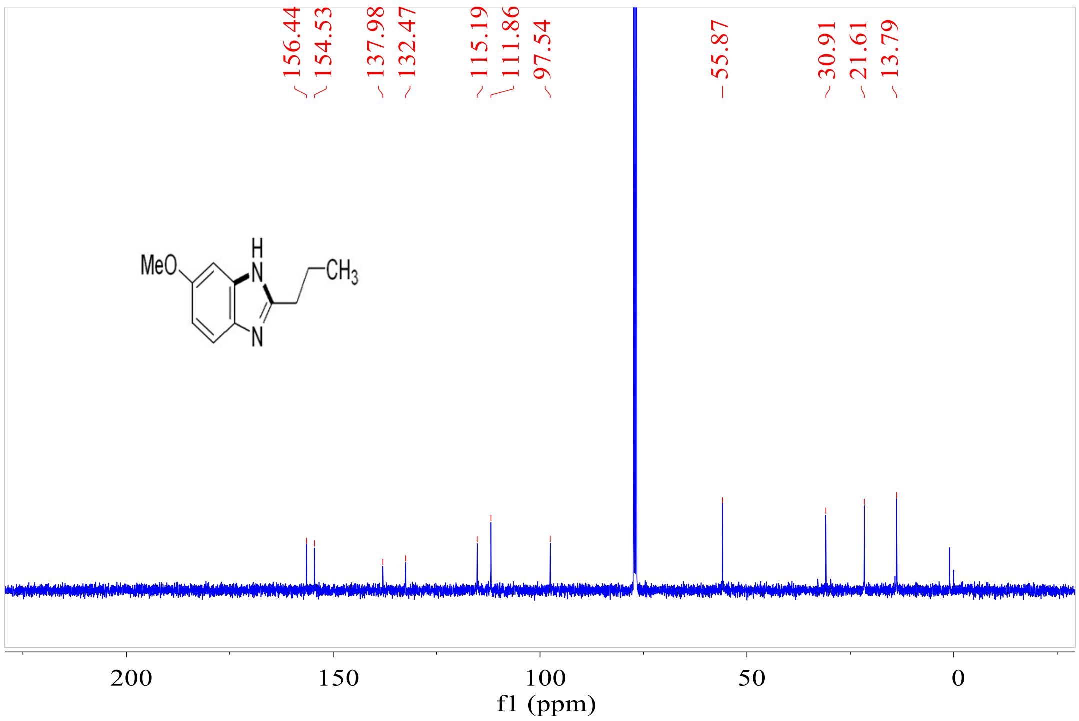

Figure S10 ^{13}C NMR of compound **2d** in $CDCl_3$

Benzimidazoles 2e

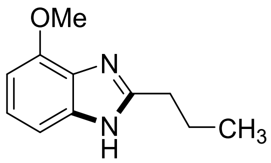

^{1}H NMR (400 MHz, $CDCl_3$) δ 7.22 – 7.07 (m, 2H), 6.67 (dd, J = 7.5, 1.2 Hz, 1H), 3.94 (s, 3H), 2.90 (t, J = 7.4 Hz, 2H), 2.03 – 1.76 (m, 2H), 0.99 (t, J = 7.4 Hz, 3H);

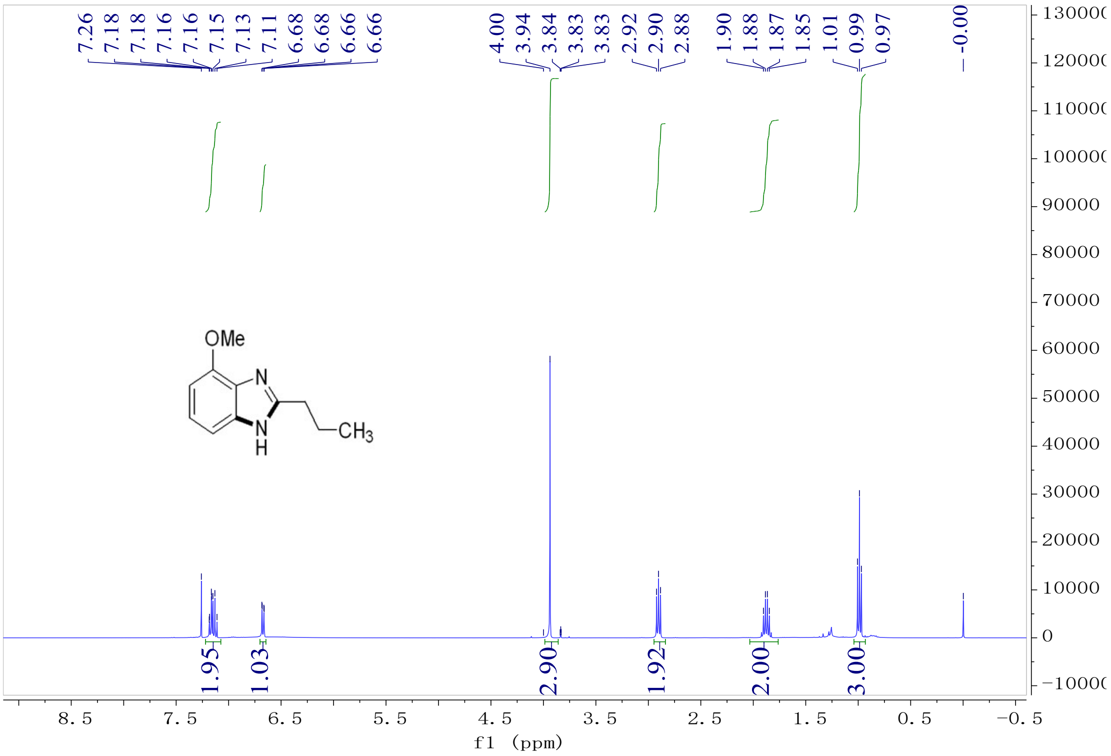

Figure S11 ^{1}H NMR of compound **2e** in $CDCl_3$

^{13}C NMR (100 MHz, $CDCl_3$) δ 154.0, 148.3, 139.7, 128.6, 122.6, 107.4, 102.6, 55.5, 31.1, 21.7, 13.8; HRMS (ESI): m/z $[M + H]^+$ calcd for $C_{11}H_{15}N_2O$ 191.1179, found: 191.1176.

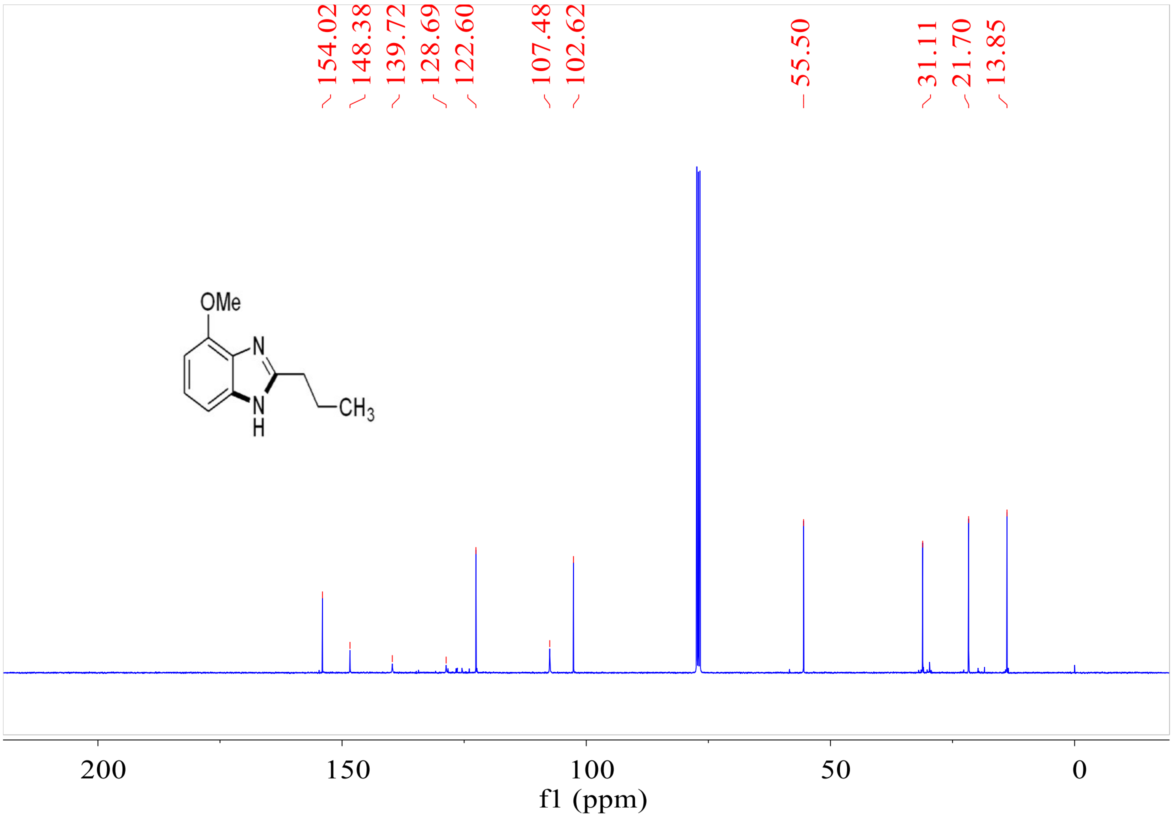

Figure S12 ^{13}C NMR of compound **2e** in $CDCl_3$

Benzimidazoles 2f

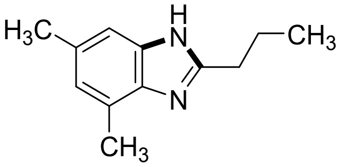

^{1}H NMR (400 MHz, CD_3OD) δ 7.34 (s, 1H), 7.20 (s, 1H), 3.16 – 3.12 (m, 2 H), 2.59 (s, 3H), 2.49 (s, 3H), 2.07 – 1.86 (m, 2H), 1.07 (t, J = 7.4 Hz, 3H)

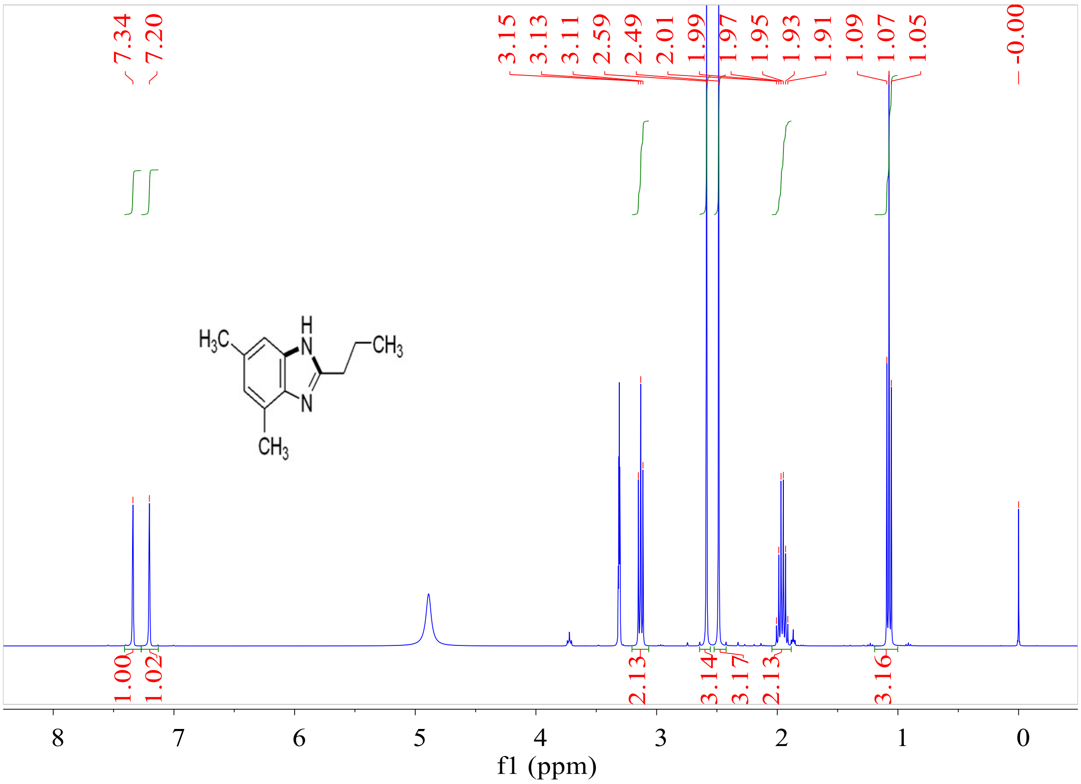

Figure S13 ^{1}H NMR of compound **2f** in CD_3OD

^{13}C NMR (100 MHz, CD_3OD) δ 153.3, 136.7, 131.0, 128.6, 127.9, 123.6, 110.1, 27.8, 20.6, 20.1, 15.1, 12.3; HRMS (ESI): m/z $[M + H]^+$ calcd for $C_{12}H_{17}N_2$ 189.1386, found: 189.1383

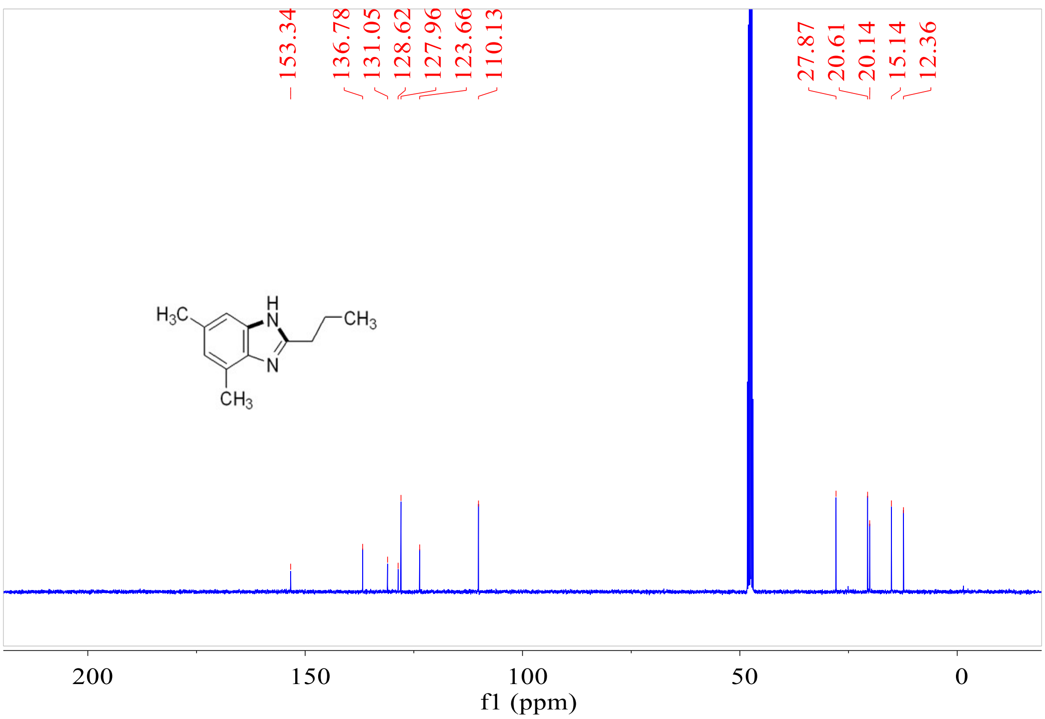

Figure S14 ^{13}C NMR of compound **2f** in CD_3OD

Benzimidazoles 2g

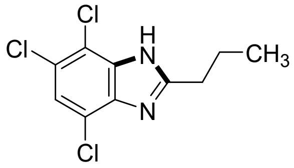

^{1}H NMR (400 MHz, DMSO-d_6) δ 13.12 (s, 1H), 7.52 (s, 1H), 2.84 (t, J = 7.5 Hz, 2H), 1.87 – 1.75 (m, 2H), 0.95 (t, J = 7.4 Hz, 3H)

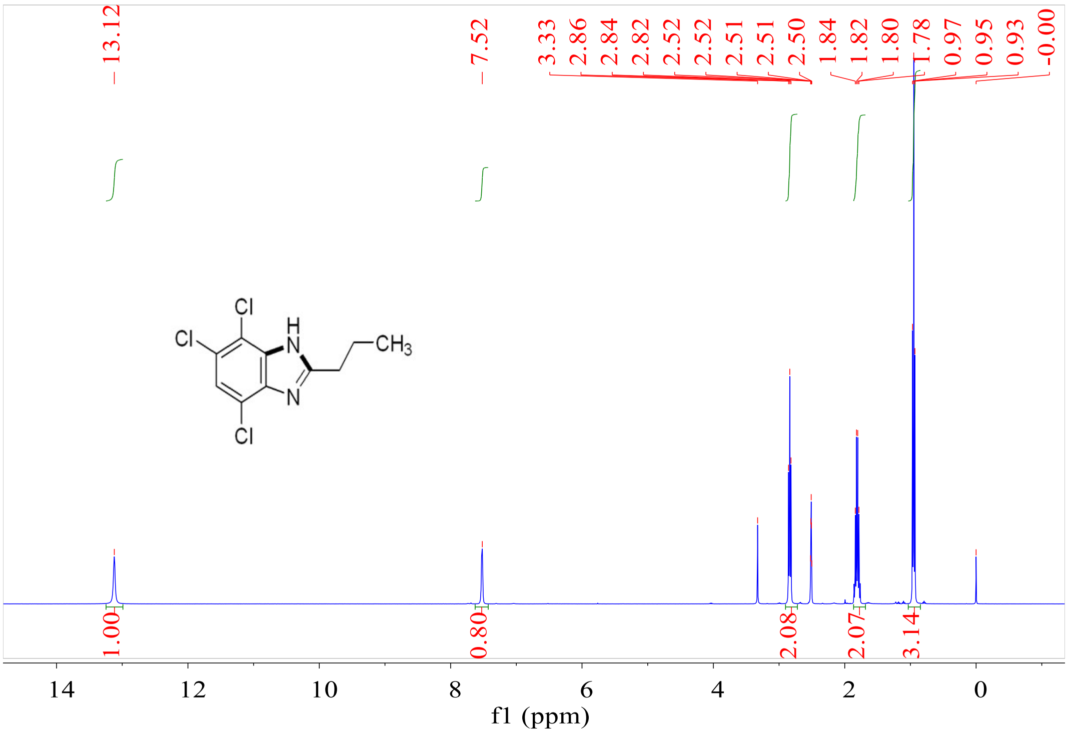

Figure S15 ^{1}H NMR of compound **2g** in DMSO-d_6

^{13}C NMR (100 MHz, CD_3OD) δ 159.6, 132.6, 131.2, 130.7, 128.1, 119.3, 117.7, 29.65, 22.6, 13.9;

HRMS (ESI): m/z $[M + H]^+$ calcd for $C_{10}H_{10}Cl_3N_2$262.9904, found: 262.9898.

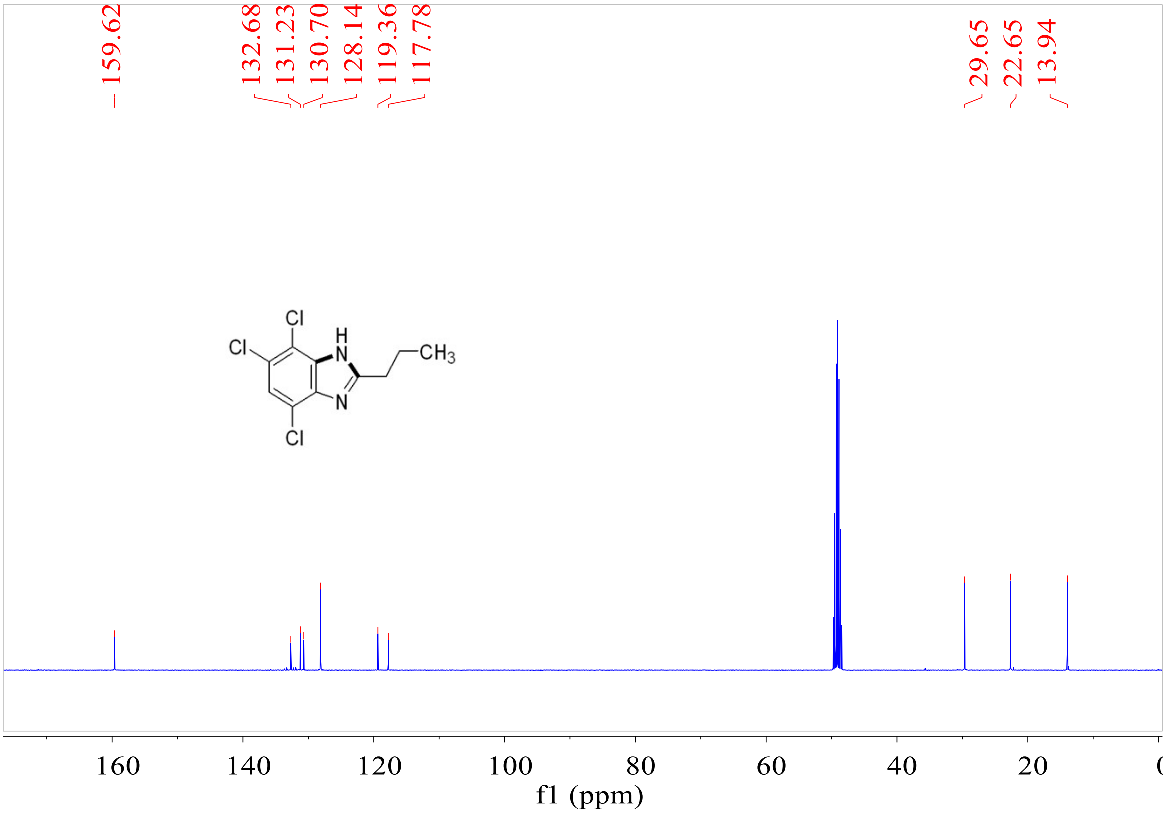

Figure S16 ^{13}C NMR of compound **2g** in CD_3OD

Benzimidazoles 2h

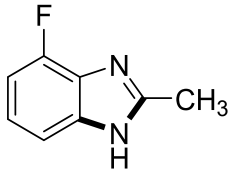

Two sets of ¹HNMR data representing two isomers (3:1) were observed as indicative of the presence of tautomerism; ¹H NMR (400 MHz, DMSO-d_6, major isomer) δ 12.47 (s, 1H), 7.24 (d, J = 8.0 Hz, 1H), 7.13 – 7.03 (m, 1H), 6.99 – 6.84 (m, 1H), 2.49 (s, 3H). ¹HNMR (400 MHz, DMSO-d_6, minor isomer) δ 12.71 (s, 1H), 7.34 (d, J = 7.6 Hz, 1H), 7.13 – 7.03 (m, 1H), 7.01 – 6.89 (m, 1H), 2.49 (s, 3H).

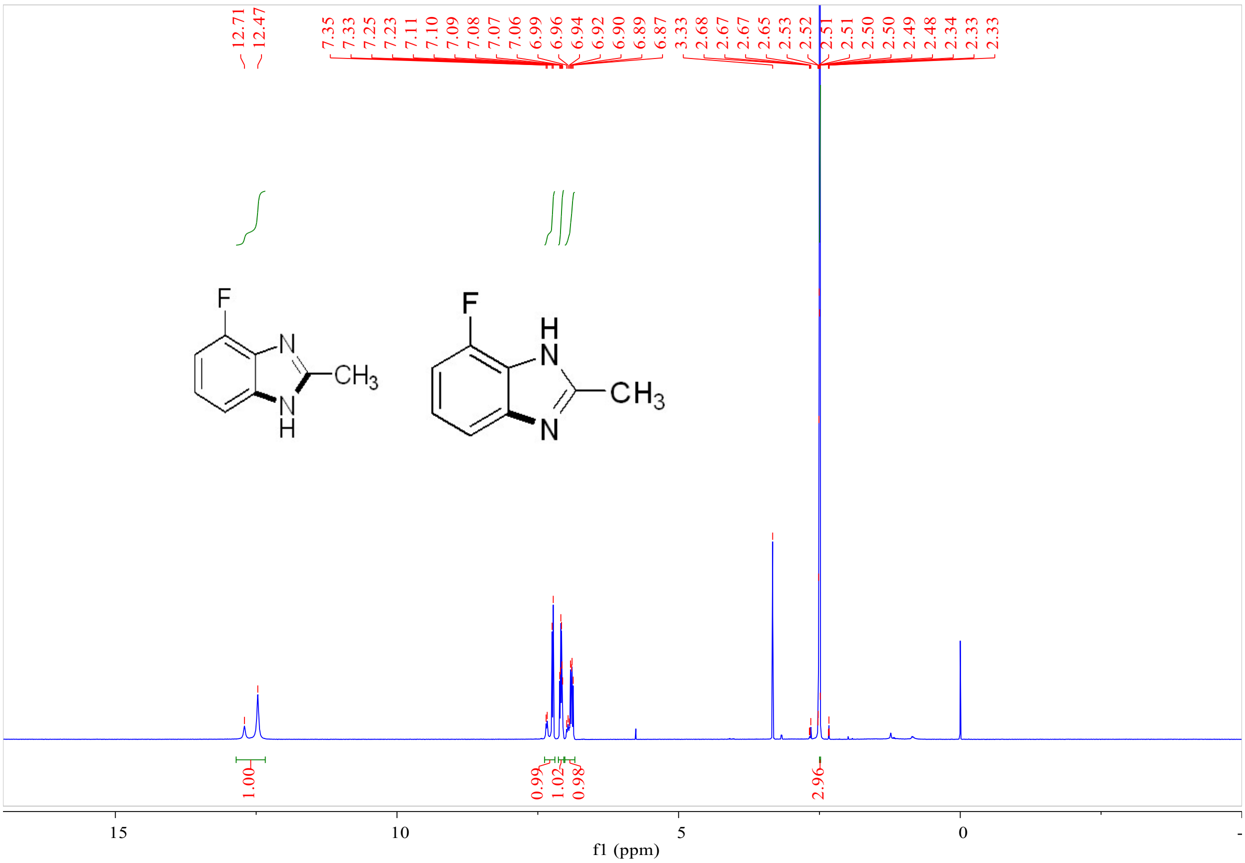

Figure S17 ¹H NMR of compound **2h** in DMSO-d_6

^{13}C NMR (101 MHz, DMSO) δ 154.2, 152.3, 151.7, 138.1, 138.0, 132.0, 122.3, 122.2, 107.5, 106.80, 106.6, 15.0; HRMS (ESI): m/z $[M + H]^+$ calcd for $C_8H_8FN_2$151.0666, found: 151.0662.

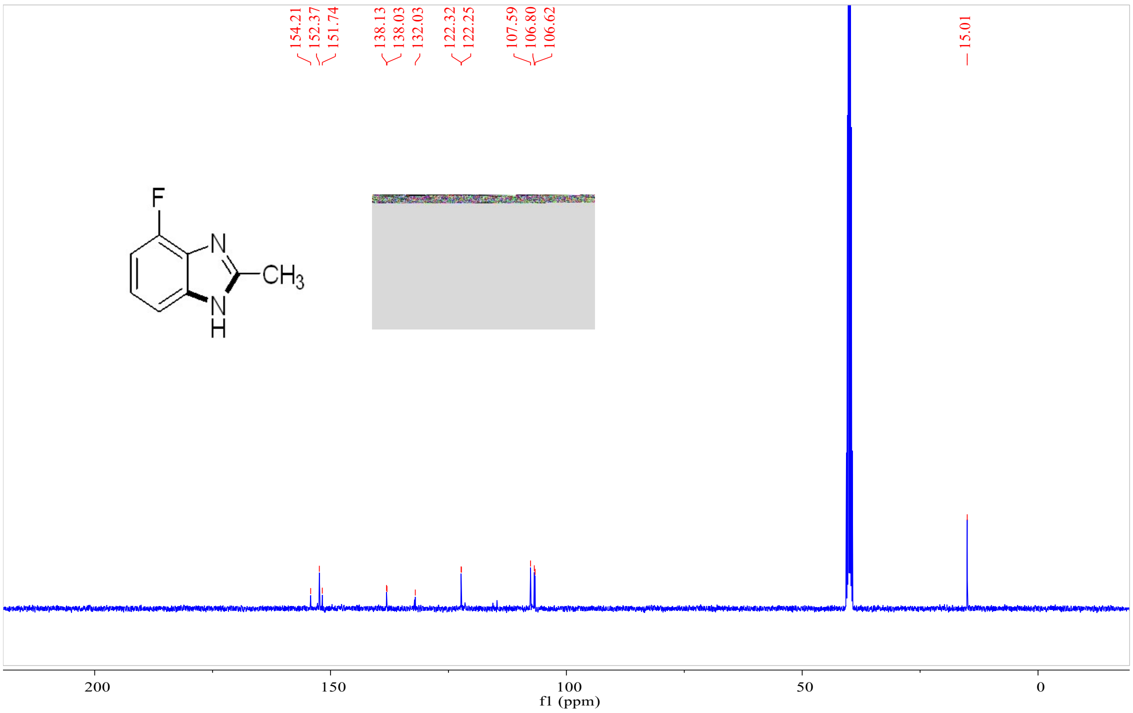

Figure S18 ^{13}C NMR of compound **2h** in DMSO-d_6

^{19}F NMR (377 MHz, DMSO) δ -135.23.

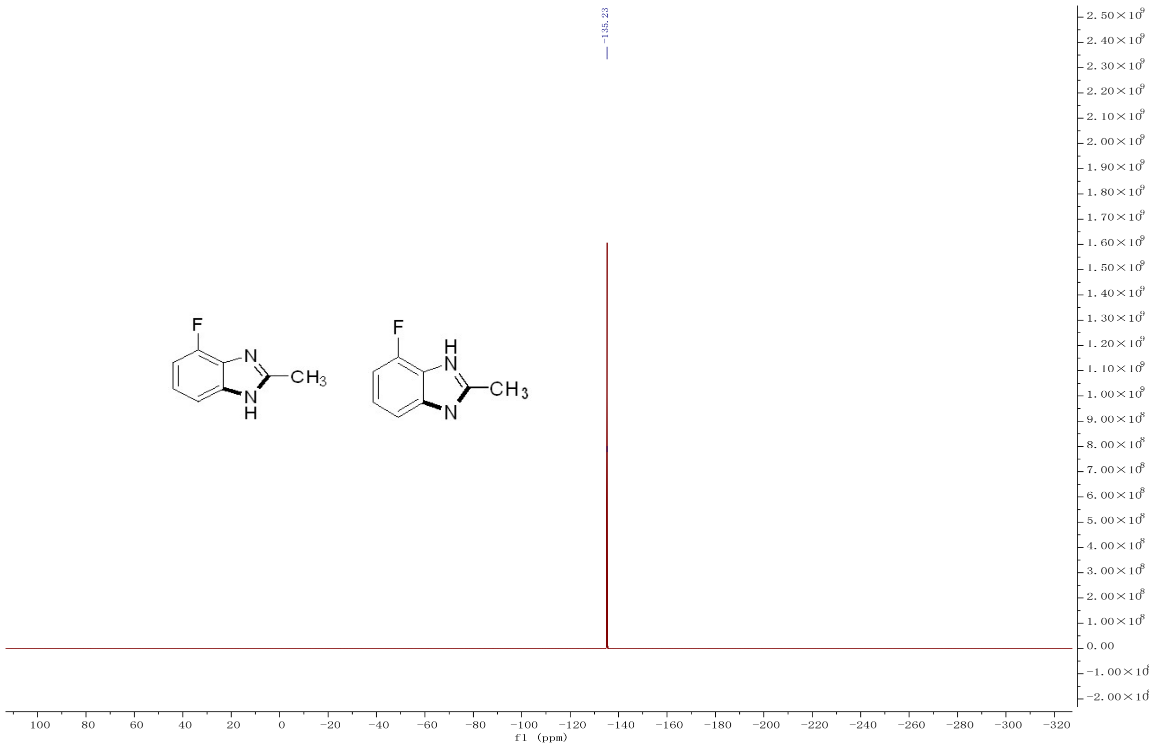

Figure S19 ^{19}F NMR of compound **2h** in DMSO-d_6

Benzimidazoles 2i

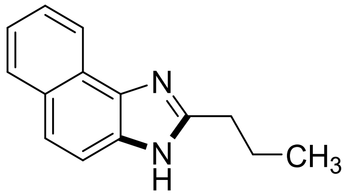

^{1}H NMR (400 MHz, CD_3OD) δ 8.36 – 8.28 (m, 1H), 8.09 – 8.01 (m, 1H), 7.97 – 7.89 (m, 1H), 7.78 – 7.67 (m, 2H), 7.67 – 7.60 (m, 1H), 3.22 (t, J = 7.7 Hz, 2H), 2.08 – 1.95 (m, 2H), 1.11 (t, J = 7.3 Hz, 3H).

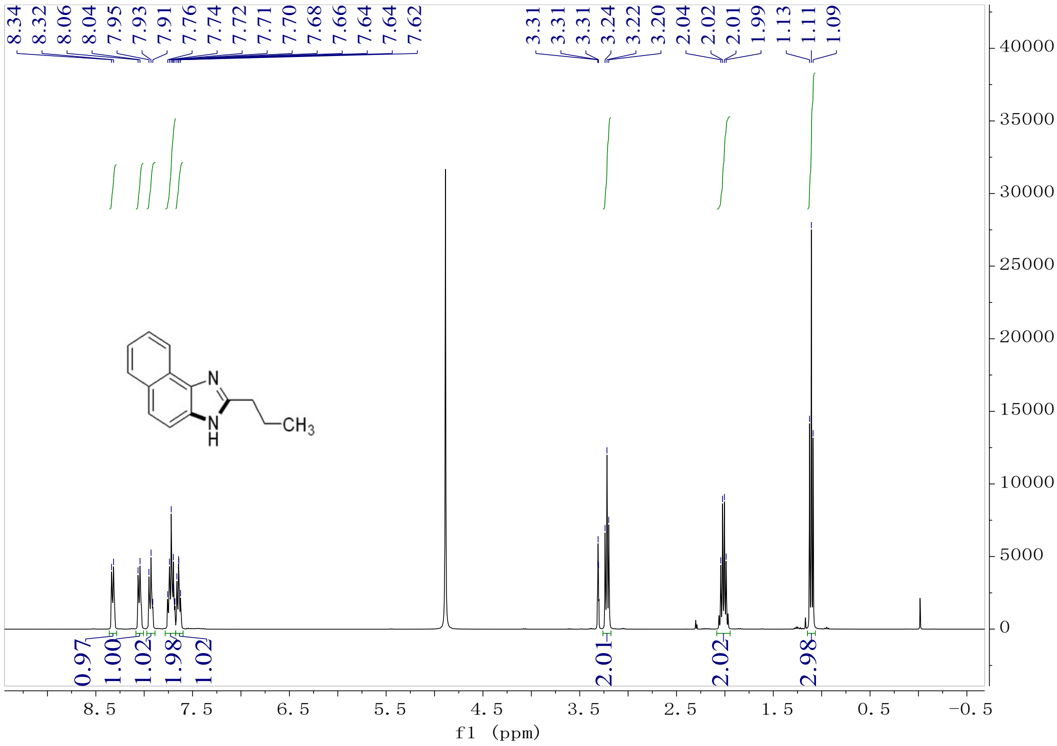

Figure S20 ^{1}H NMR of compound **2i** in CD_3OD

^{13}C NMR (100 MHz, CD_3OD) δ 153.0, 132.6, 132.6, 130.3, 129.3, 129.2, 128.6, 127.9, 122.1, 121.9, 113.1, 29.2, 22.1, 13.8; HRMS (ESI): m/z $[M + H]^+$ calcd for $C_{14}H_{15}N_2$ 211.1230, found: 211.1226.

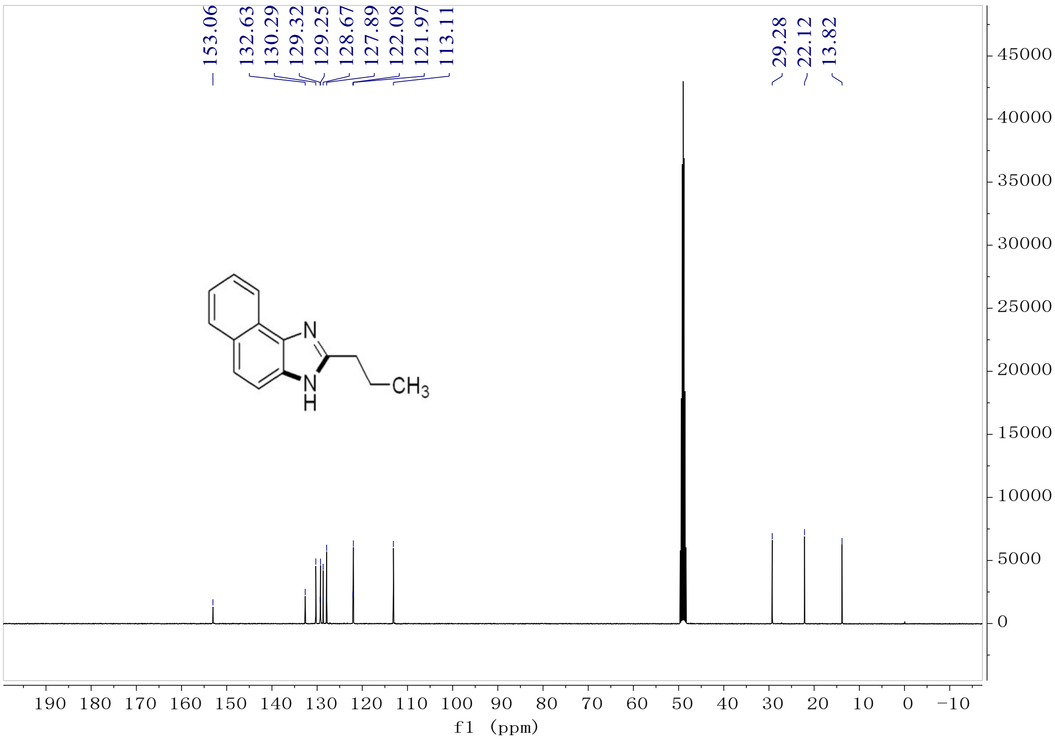

Figure S21 ^{13}C NMR of compound **2i** in CD_3OD

Benzimidazoles 2j

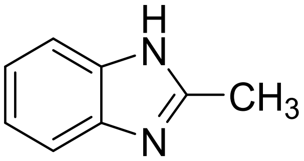

^{1}H NMR (400 MHz, DMSO-d_6) δ 12.15 (s, 1H), 7.43 (d, J = 3.2 Hz, 2H), 7.09 (dd, J = 5.9, 3.1 Hz, 2H), 2.56 – 2.21 (m, 3H).

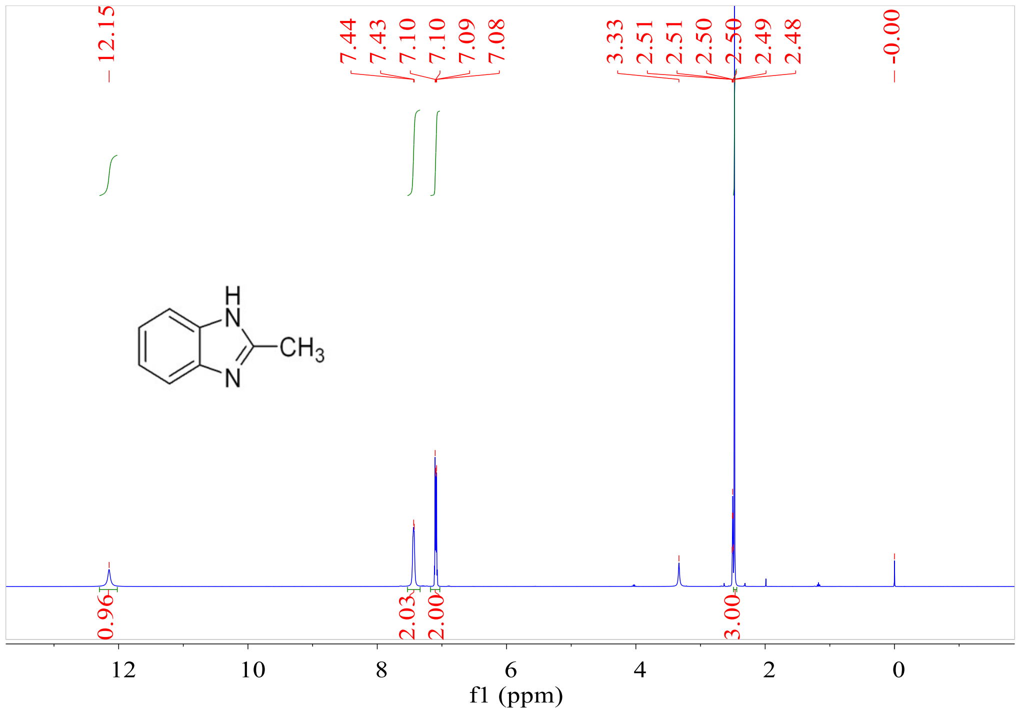

Figure S22 ^{1}HNMR of compound **2j** in DMSO-d_6

^{13}C NMR (101 MHz, DMSO) δ 151.6, 121.4, 40.6, 40.4, 15.1; HRMS (ESI): m/z $[M + H]^+$ calcd for $C_8H_9N_2$ 133.0760

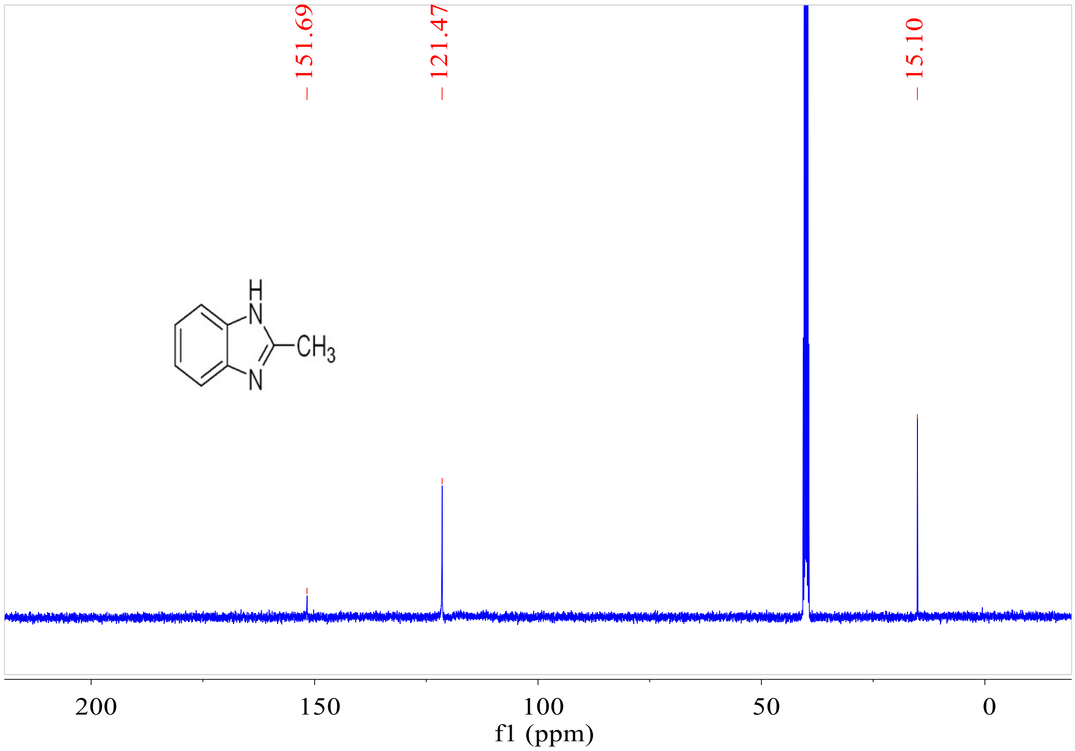

Figure S23 ^{13}CNMR of compound **2j** in DMSO-d_6

Benzimidazoles 2k

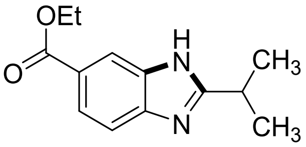

^{1}H NMR (400 MHz, $CDCl_3$) δ 8.41 (d, J = 0.7 Hz, 1H), 8.26 (dd, J = 8.6, 1.4 Hz, 1H), 7.86 (d, J = 8.6 Hz, 1H), 4.46 (q, J = 7.1 Hz, 2H), 3.59 (dt, J = 14.0, 7.0 Hz, 1H), 1.60 (d, J = 7.0 Hz, 6H), 1.45 (t, J = 7.1 Hz, 3H)

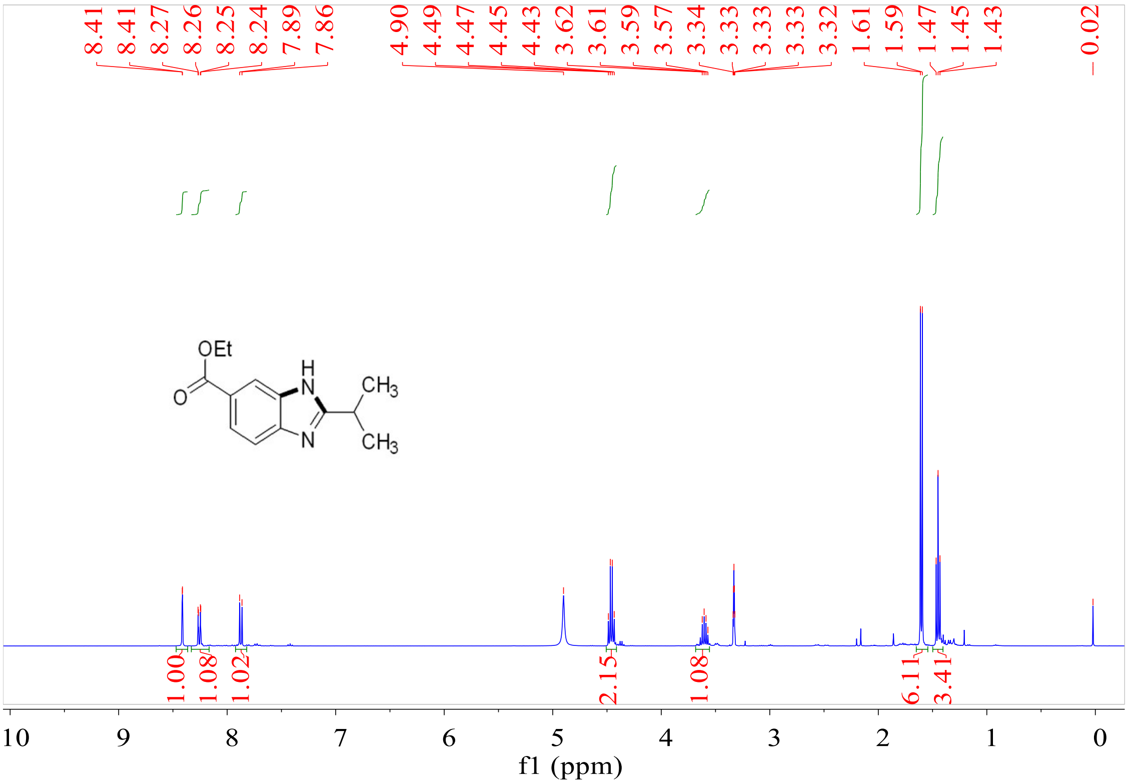

Figure S24 ^{1}HNMR of compound **2k** in $CDCl_3$

$^{13}C\{^{1}H\}$ NMR (100 MHz, CD_3OD) δ 166.8, 162.5, 135.2, 132.1, 129.9, 128.2, 116.5, 114.8, 62.8, 29.1, 20.3, 14.6; HRMS (ESI): m/z $[M + H]^+$ calcd for $C_{13}H_{17}N_2O_2$ 233.1285, found: 233.1282.

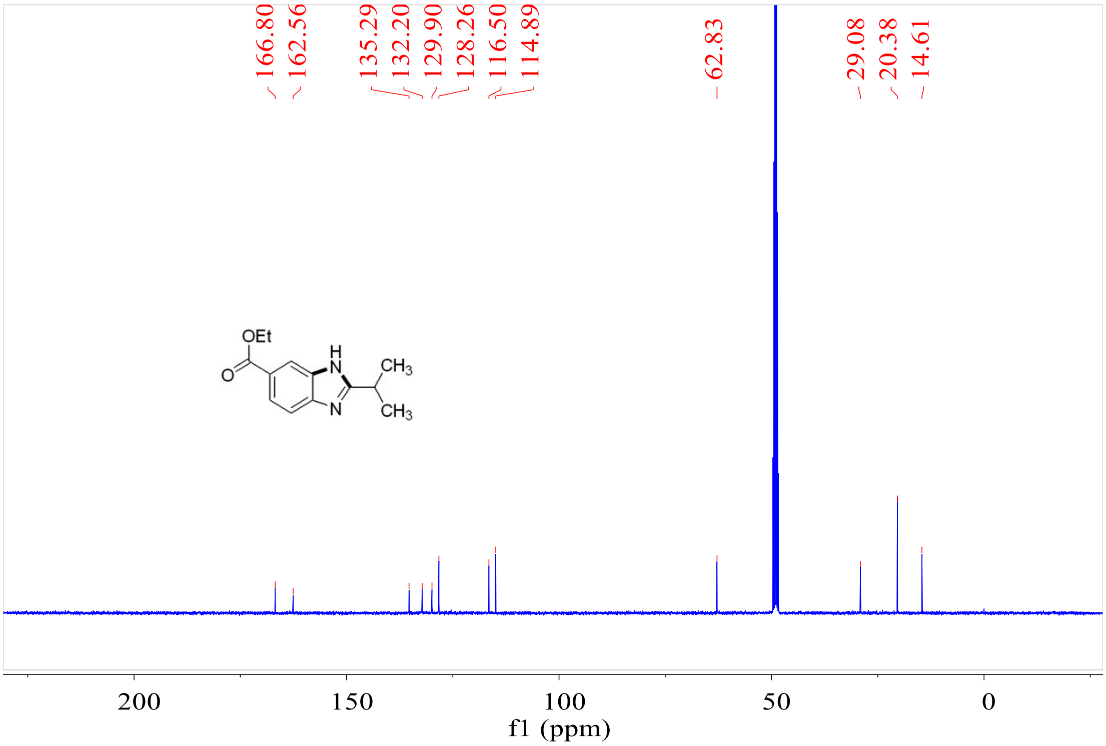

Figure S25 ^{13}CNMR of compound **2k** in CD_3OD

Benzimidazoles 2l

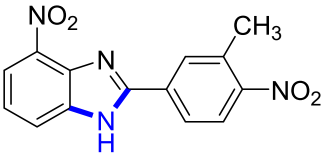

^{1}H NMR (400 MHz, DMSO-d_6) δ 9.76 – 9.38 (s, 1H), 8.03 – 7.94 (m, 2H), 7.71 – 7.67 (m, 1H), 7.53 – 7.51 (s, 1H), 7.31 – 7.24 (m, 1H), 7.03 – 6.97 (dd, J = 8.1, 1.0 Hz, 1H), 2.24 – 1.93 (s, 3H).

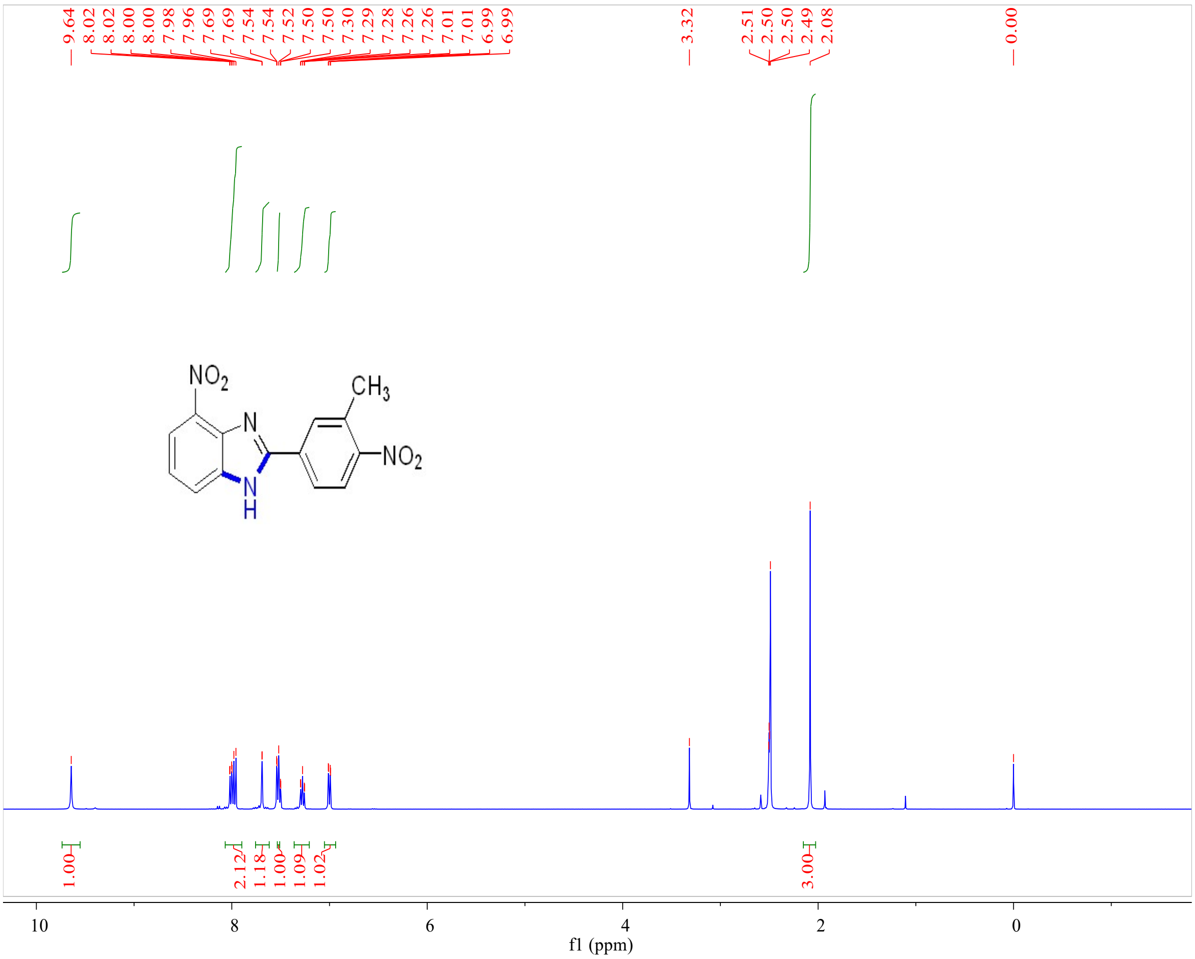

Figure S26 ^{1}HNMR of compound **2l** in DMSO-d_6

$^{13}C\{^{1}H\}$ NMR (100 MHz, DMSO-d_6) δ 167.9, 152.8, 150.4, 142.1, 135., 134.5, 134.4, 133.4, 133.4, 128.1, 127.1, 125.8, 125.2, 125.1, 19.7; HRMS (ESI): m/z $[M + H]^+$ calcd for $C_{14}H_{14}N_4O_4$ 299.0775, found: 299.0772.

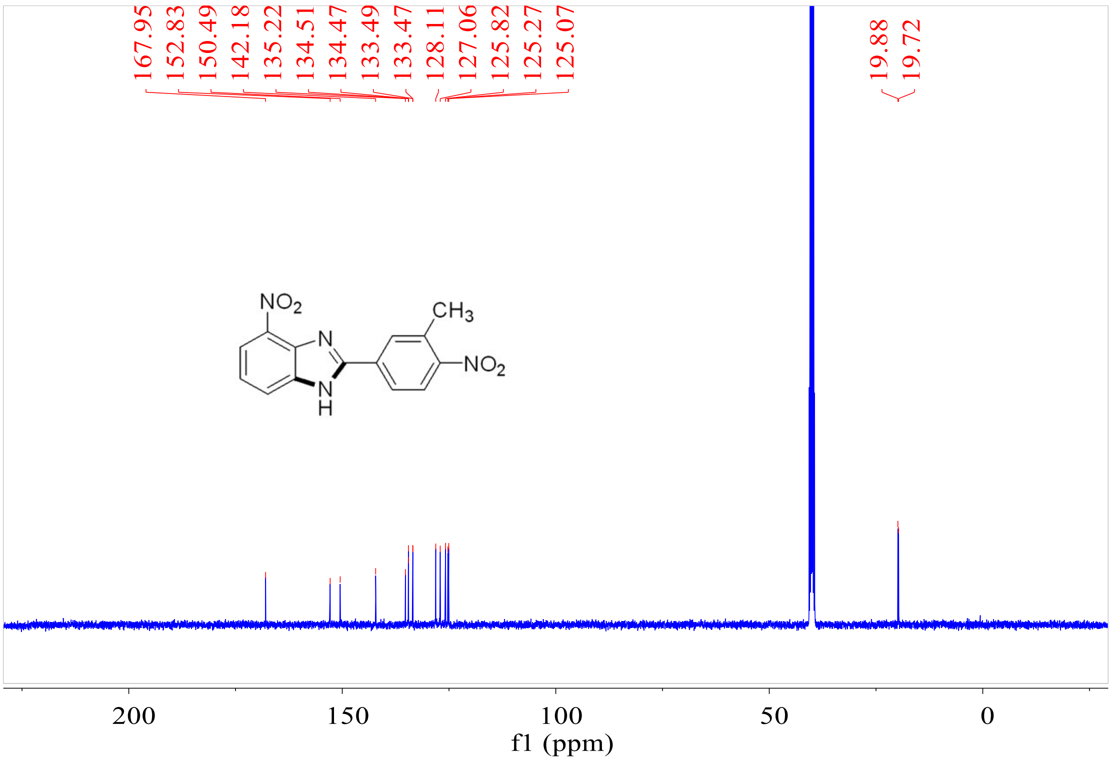

Figure S27 ^{13}CNMR of compound **2l** in DMSO-d_6

Benzimidazoles **2m**

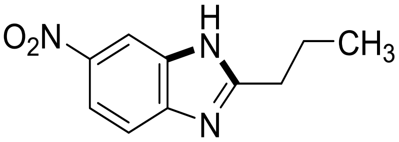

^{1}H NMR (400 MHz, CD_3OD) δ 8.66 (d, J = 1.9 Hz, 1H), 8.46 (dd, J = 9.0, 2.1 Hz, 1H), 7.97 (d, J = 9.0 Hz, 1H), 3.28 – 3.20 (m, 2H), 2.07 – 1.95 (m, 2H), 1.12 (t, J = 7.4 Hz, 3H).

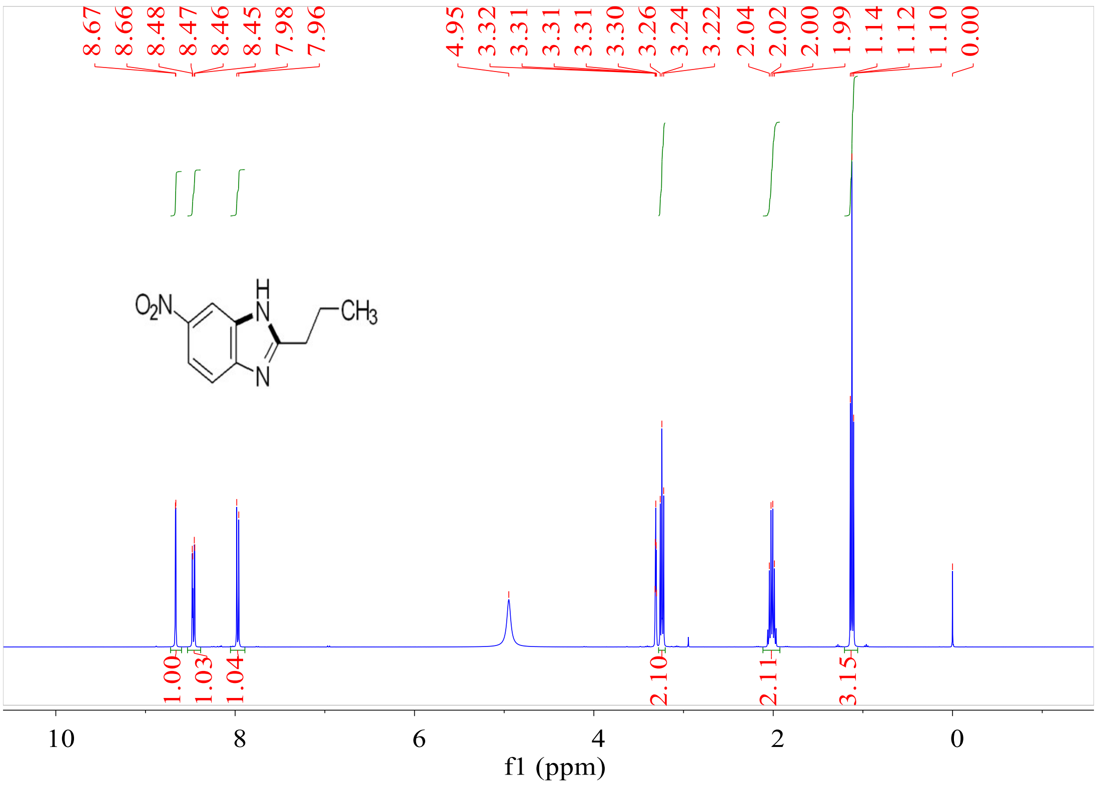

Figure S28 ^{1}HNMR of compound **2m** in CD_3OD

$^{13}C\{^1H\}$ NMR (100 MHz, CD_3OD) δ 158.8, 145.8, 134.8, 130.7, 121.1, 114.3, 110.1, 28.3, 20.1, 12.3; HRMS (ESI): m/z $[M + H]^+$ calcd for $C_{10}H_{12}N_3O_2$ 206.0924, found: 206.0921.

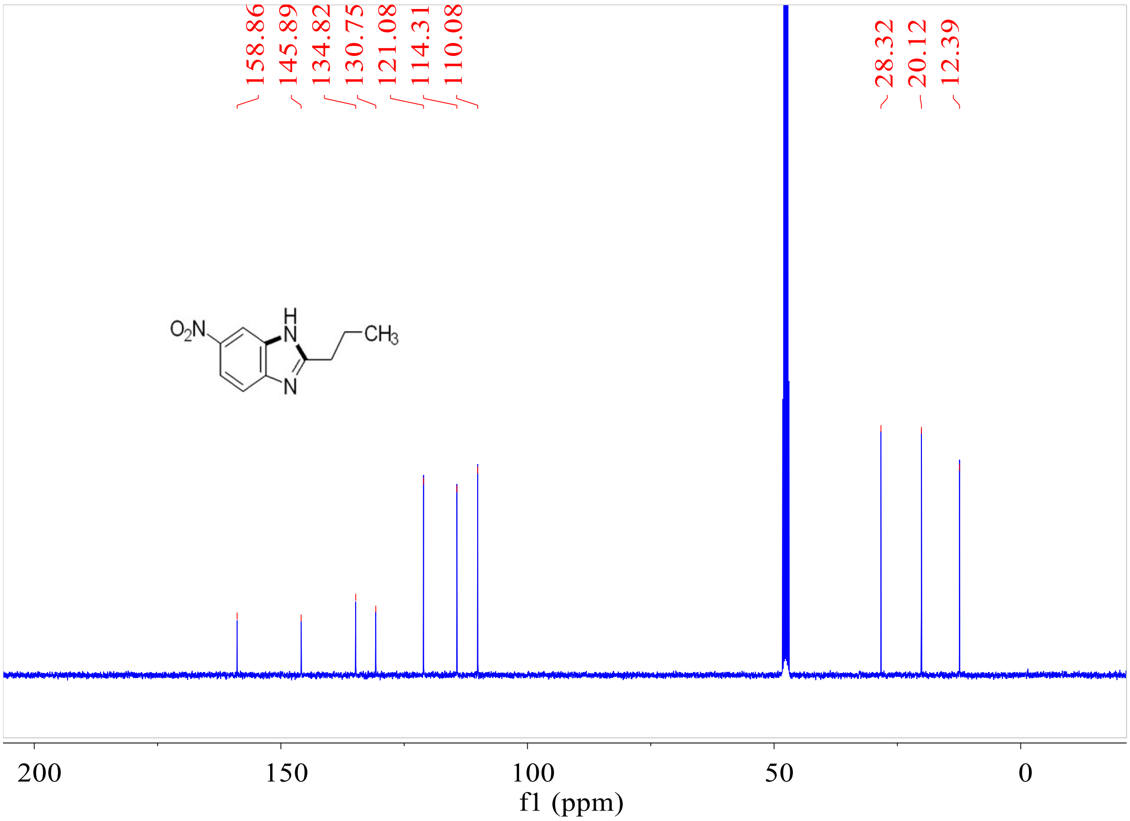

Figure S29 ^{13}CNMR of compound **2m** in CD_3OD

Benzimidazoles 2n

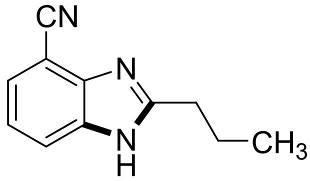

^{1}H NMR (400 MHz, CD_3OD) δ 8.12 (d, J = 8.3 Hz, 1H), 8.02 (d, J = 7.7 Hz, 1H), 7.75 (t, J = 8.0 Hz, 1H), 3.24 (t, J = 7.7 Hz, 2H), 2.12 – 1.93 (m, 2H), 1.13 (t, J = 7.4 Hz, 3H).

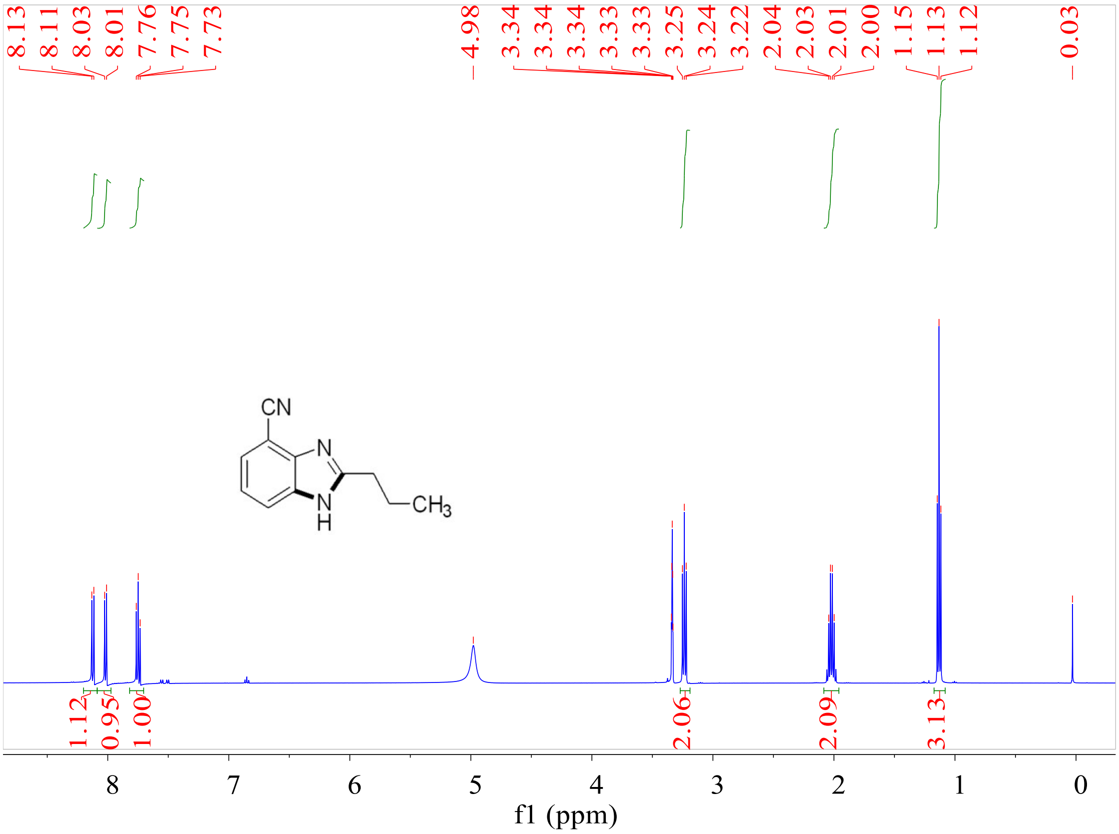

Figure S30 ^{1}HNMR of compound **2n** in CD_3OD

$^{13}C\{^{1}H\}$ NMR (100 MHz, CD_3OD) δ 157.0, 131.8, 131.6, 130.5, 126.1, 118.7, 113.9, 97.9, 28.1, 20.4, 12.4; HRMS (ESI): m/z $[M + H]^+$ calcd for $C_{11}H_{12}N_3$ 186.1026, found: 186.1024.

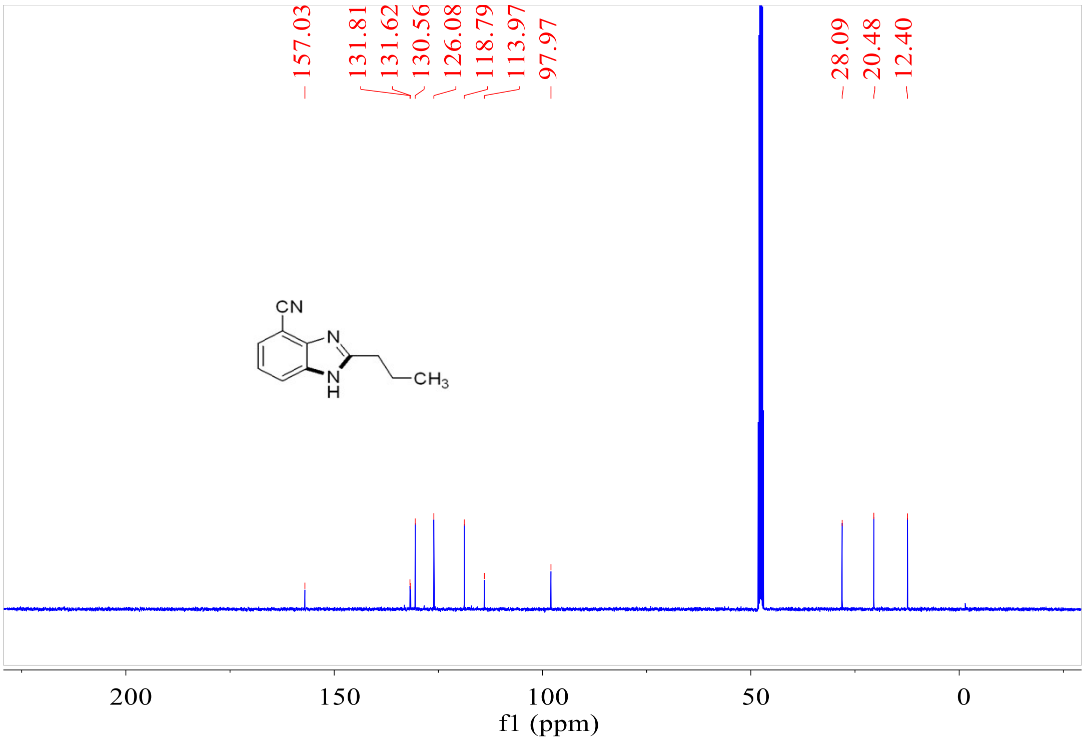

Figure S31 ^{13}CNMR of compound **2n** in CD_3OD

Benzimidazoles 2o

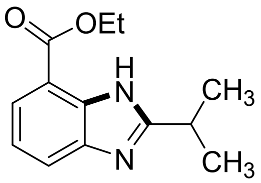

2o ^{1}H NMR (400 MHz, CD_3OD) δ 8.22 (d, J = 7.7 Hz, 1H), 8.04 (d, J = 8.1 Hz, 1H), 7.76 – 7.68 (m, 1H), 4.57 (q, J = 7.1 Hz, 1H), 3.72 (dd, J = 14.0, 7.0 Hz, 1H), 1.59 (d, J = 7.0 Hz, 1H), 1.48 (t, J = 7.1 Hz, 1H)

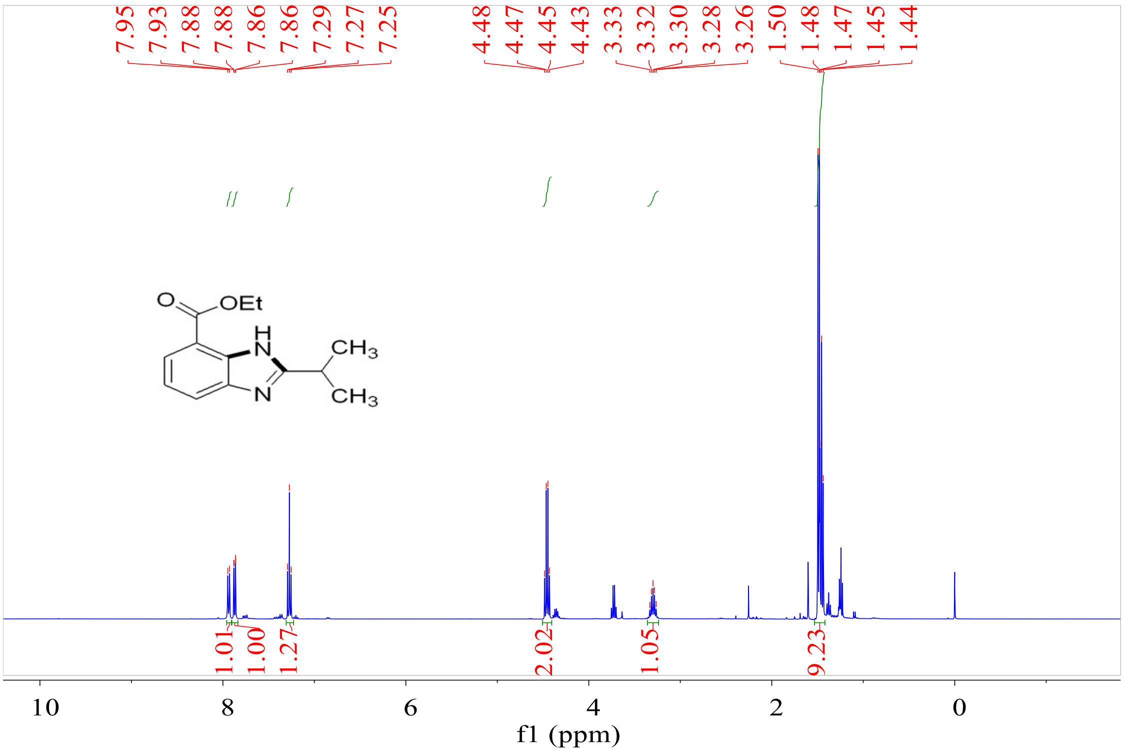

Figure S32 ^{1}HNMR of compound **2o** in CD_3OD

$^{13}C\{^1H\}$ NMR (100 MHz, CD_3OD) δ 165.8, 162.5, 134.8, 131.8, 128.3, 126.4, 120.4, 117.9, 62.9, 28.8, 21.0, 14.7; HRMS (ESI): m/z $[M + H]^+$ calcd for $C_{13}H_{17}N_2O_2$ 233.1285, found: 233.1283.

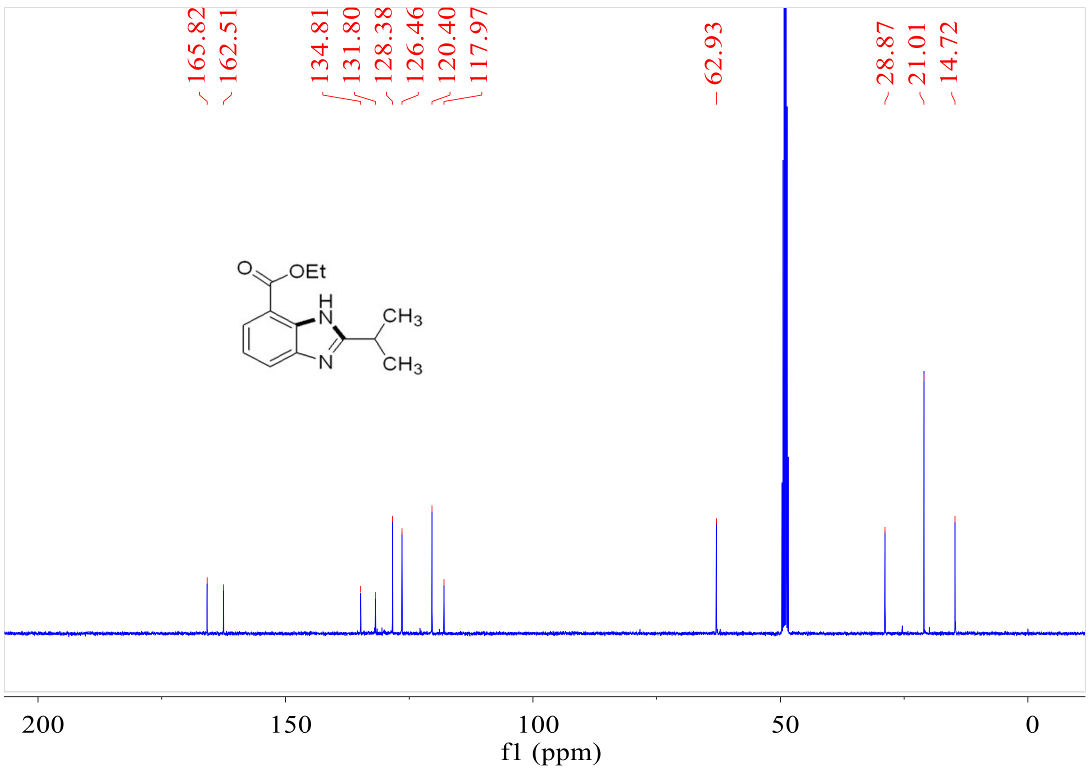

Figure S33 ^{13}CNMR of compound **2o** in CD_3OD

Benzimidazoles 2p

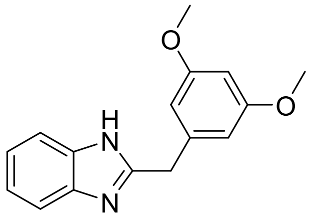

^{1}H NMR (400 MHz, $CDCl_3$) δ 7.52 (s, 2H), 7.21 (dd, J = 6.0, 3.2 Hz, 2H), 6.44 (d, J = 2.1 Hz, 2H), 6.37 (d, J = 2.1 Hz, 1H), 4.21 (s, 6H), 3.73 (s, 3H).

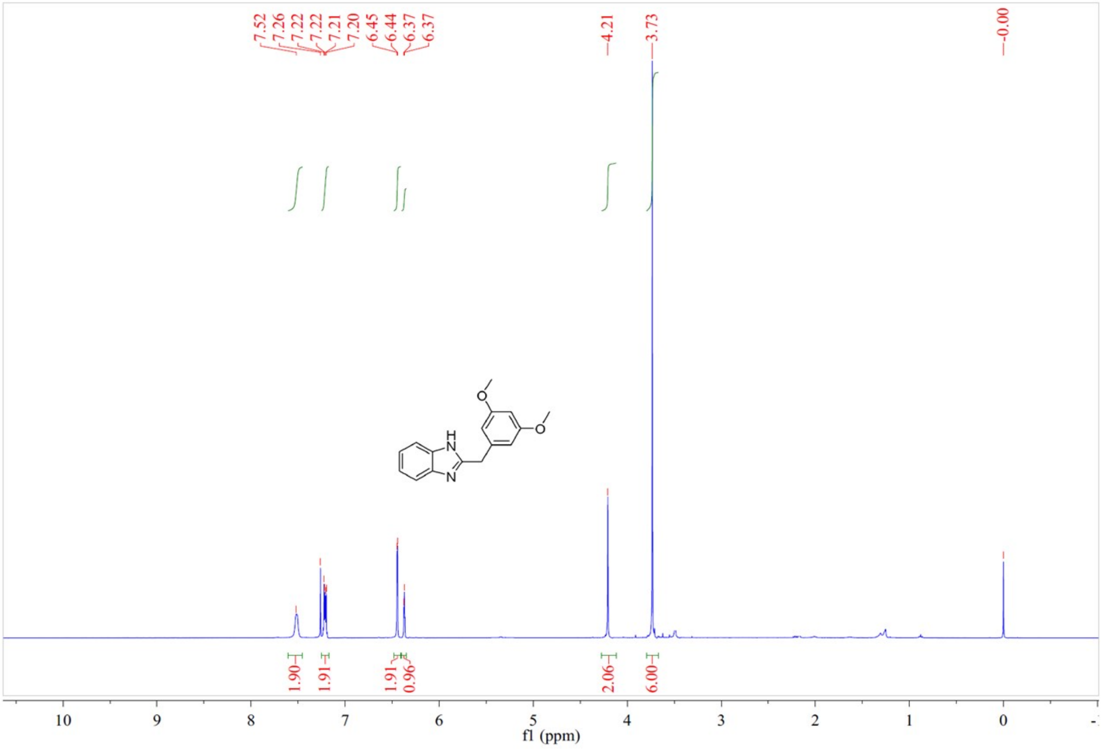

Figure S34 ^{1}HNMR of compound **2p** in $CDCl_3$

^{13}C NMR (101 MHz, $CDCl_3$) δ 161.3, 153.0, 138.3, 122.4, 107.1, 99.2, 55.3, 36.2; HRMS (ESI): m/z $[M + H]^+$ calcd for $C_{16}H_{17}N_2O_2$ 269.1285

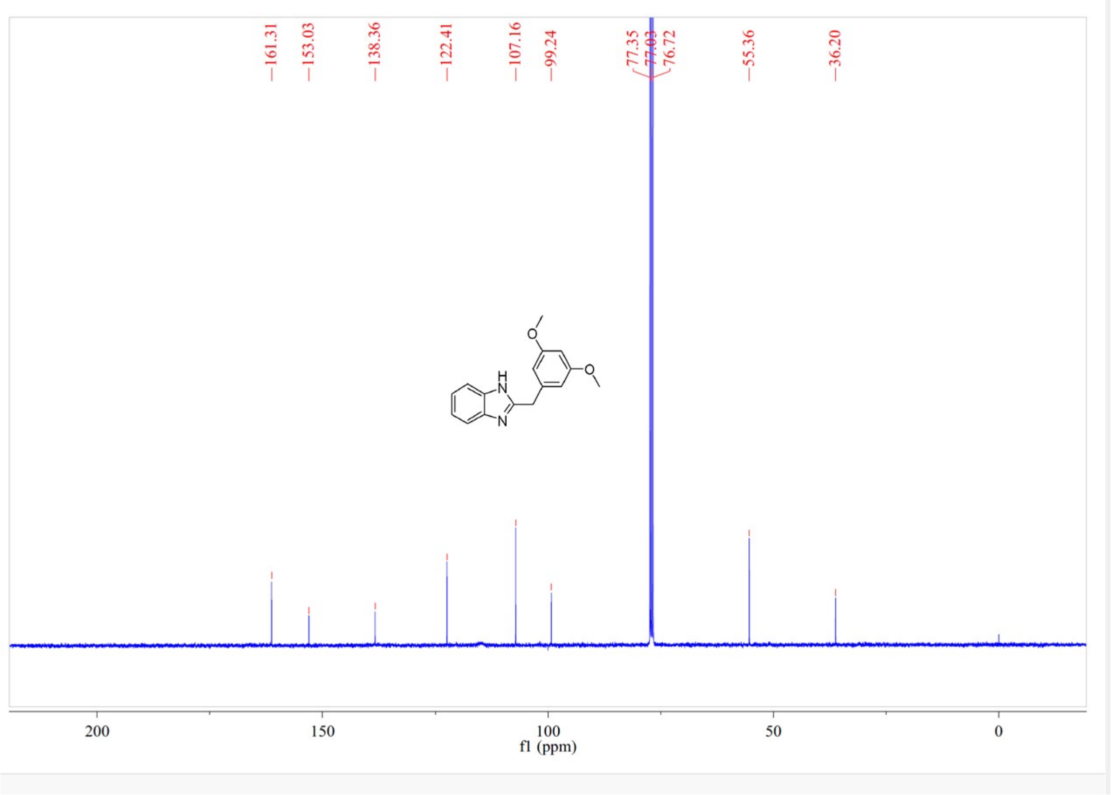

Figure S35 ^{13}CNMR of compound **2p** in $CDCl_3$

Benzimidazoles 2q

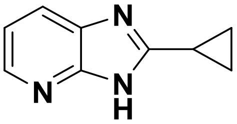

^{1}H NMR (400 MHz, CD_3OD) δ 8.24 (d, *J* = 4.4 Hz, 1H), 7.83 (dd, *J* = 8.0, 1.3 Hz, 1H), 7.21 (dd, *J* = 8.0, 4.9 Hz, 1H), 2.19 (s, 0H), 1.43 – 1.03 (m, 4H).

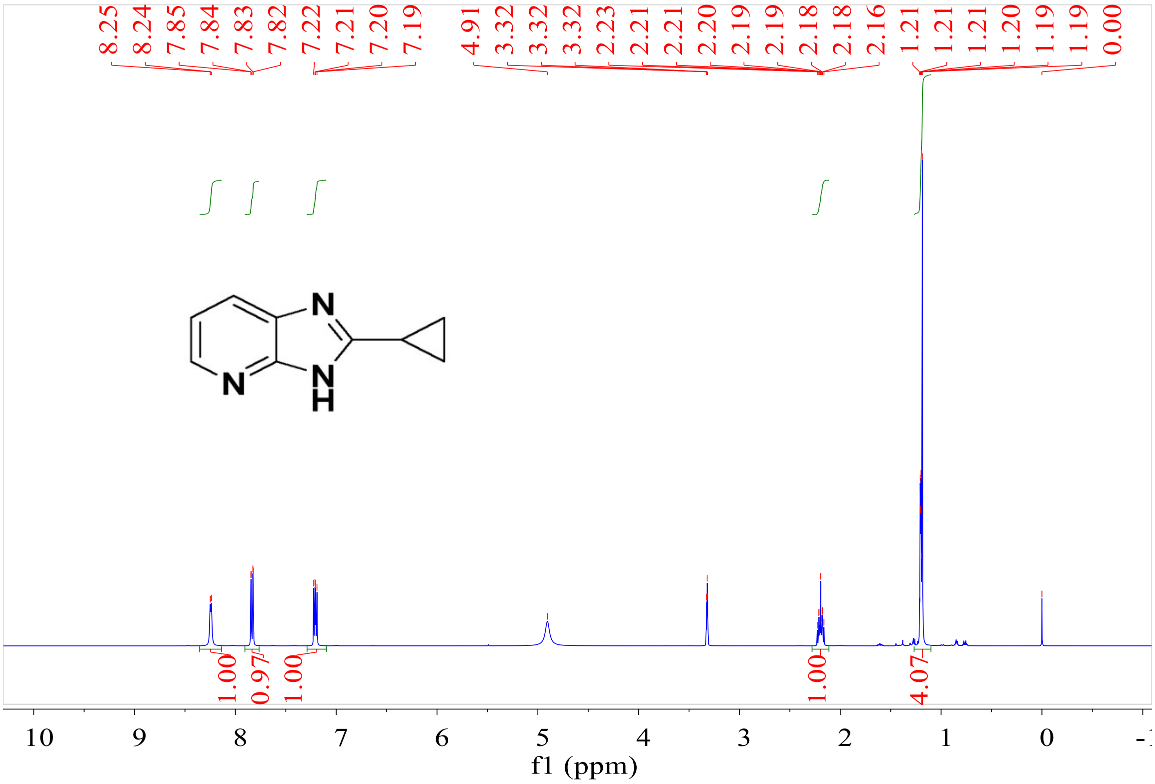

Figure S36 ^{1}HNMR of compound **2q** in CD_3OD

^{13}C NMR (101 MHz, CD_3OD) δ 160.4, 151.6, 142.4, 130.7, 121.8, 117.5, 9.2, 8.4; HRMS (ESI): m/z $[M + H]^+$ calcd for $C_9H_{10}N_3$ 160.0869

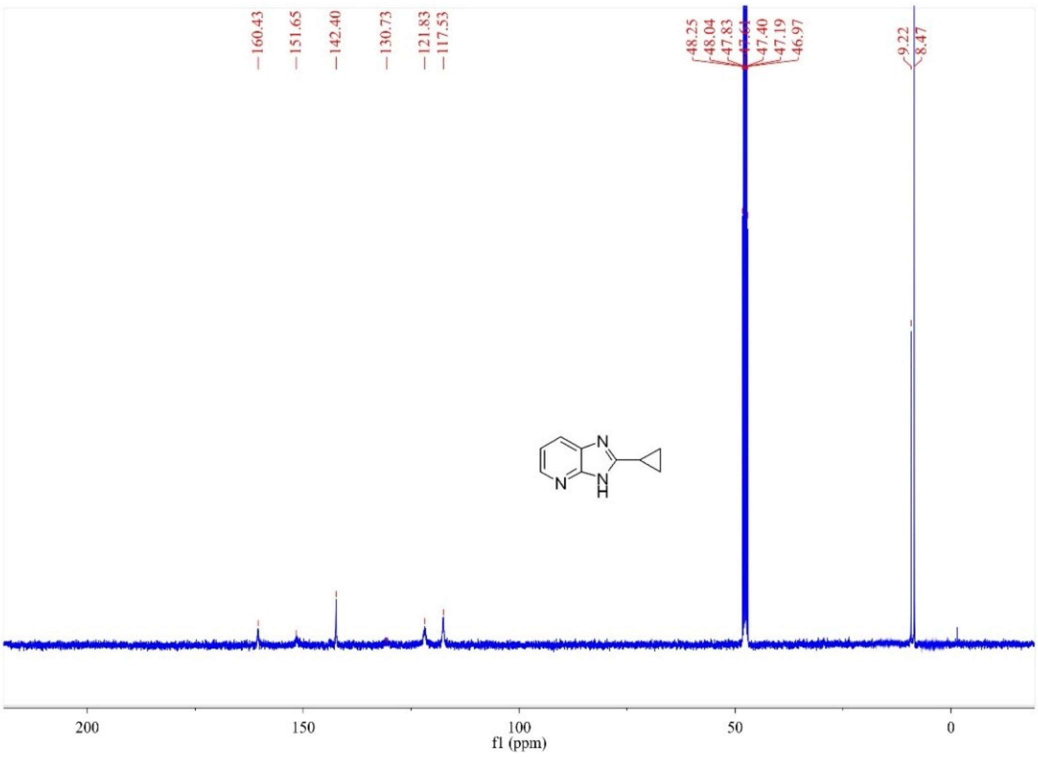

Figure S37 ^{1}HNMR of compound **2q** in CD_3OD

Benzimidazoles 2r

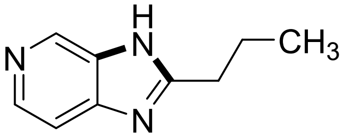

^{1}H NMR (400 MHz, CD_3OD) δ 8.62 (dd, *J* = 5.2, 1.2 Hz, 1H), 8.33 (dd, *J* = 8.2, 1.3 Hz, 1H), 7.66 (dd, *J* = 8.2, 5.2 Hz, 1H), 3.16 (t, *J* = 7.6 Hz, 2H), 1.98 (q, *J* = 7.5 Hz, 2H), 1.09 (t, *J* = 7.4 Hz, 3H).

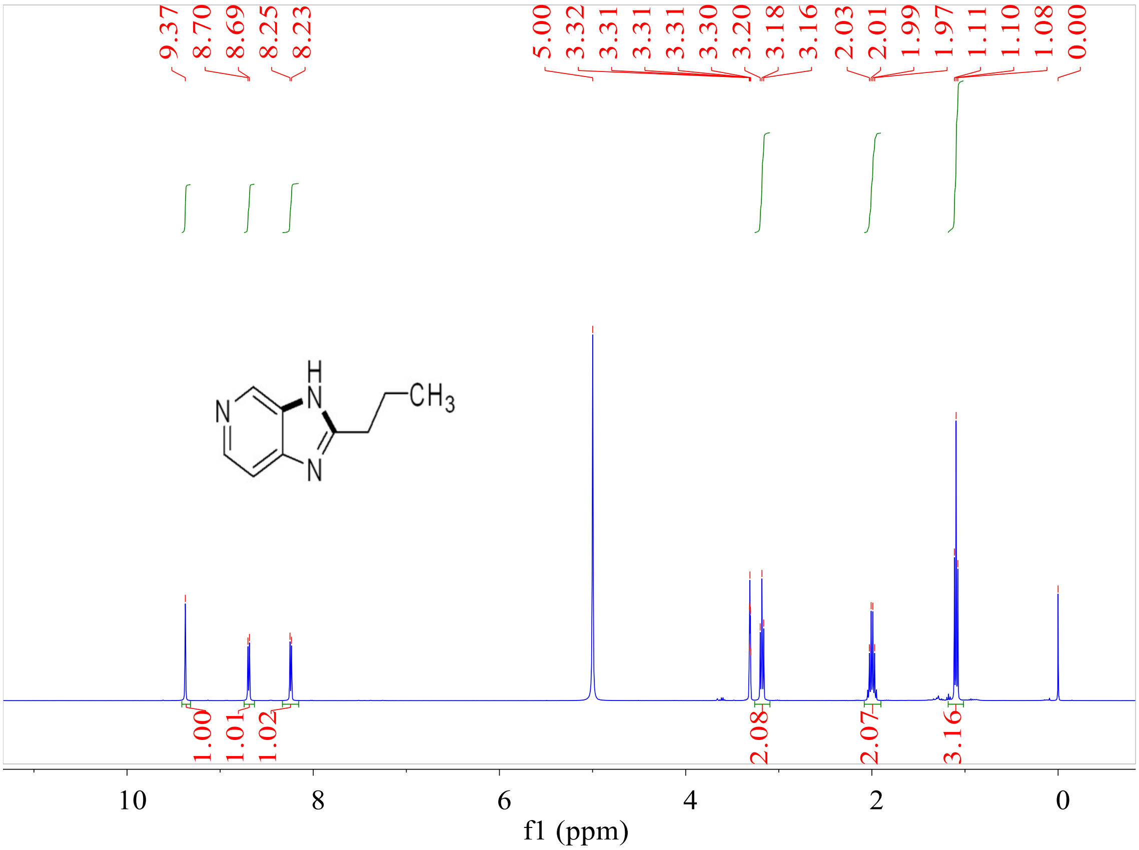

Figure S38 ^{1}HNMR of compound **2r** in CD_3OD

^{13}C NMR (101 MHz, CD_3OD) δ 164.7, 145.0, 135.1, 134.6, 131.5, 111.2, 29.8, 20.4, 12.5; HRMS (ESI): m/z $[M + H]^+$ calcd for $C_9H_{12}N_3$ 162.1026.

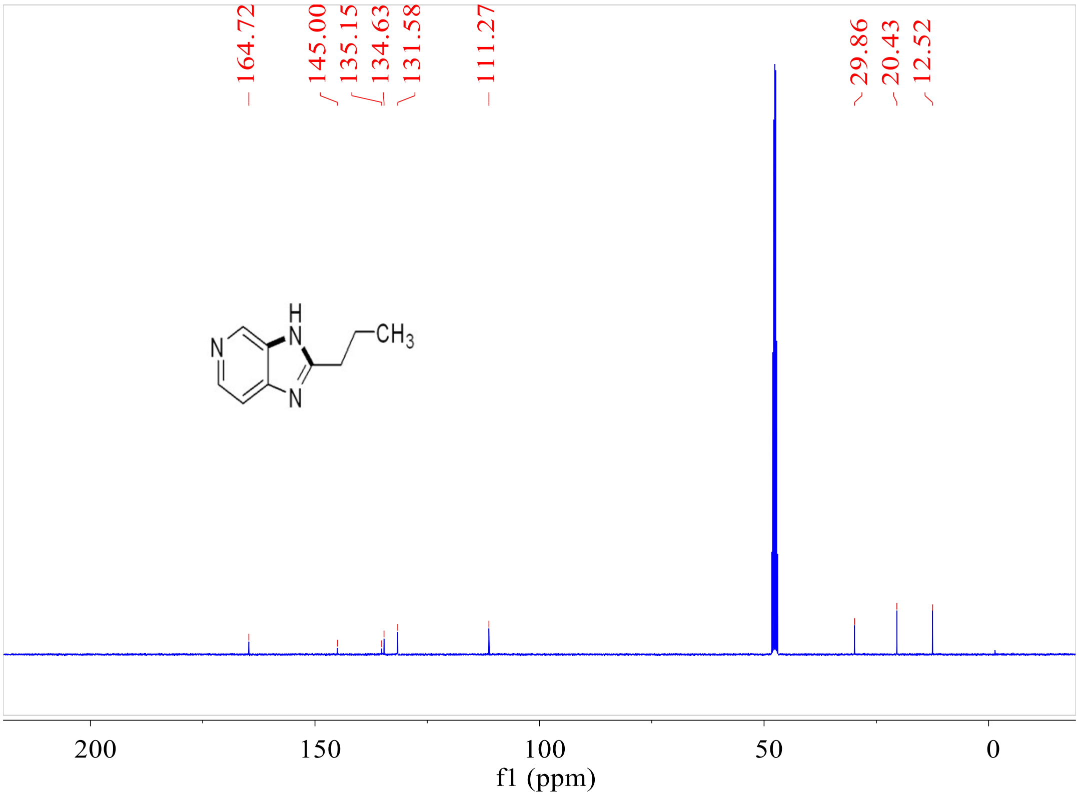

Figure S39 ^{13}CNMR of compound **2r** in CD_3OD

Benzimidazoles 2s

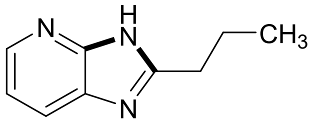

^{1}H NMR (400 MHz, CD_3OD) δ 8.62 (dd, *J* = 5.2, 1.2 Hz, 1H), 8.33 (dd, *J* = 8.2, 1.3 Hz, 1H), 7.66 (dd, *J* = 8.2, 5.2 Hz, 1H), 3.16 (t, *J* = 7.6 Hz, 2H), 1.98 (q, *J* = 7.5 Hz, 2H), 1.09 (t, *J* = 7.4 Hz, 3H).

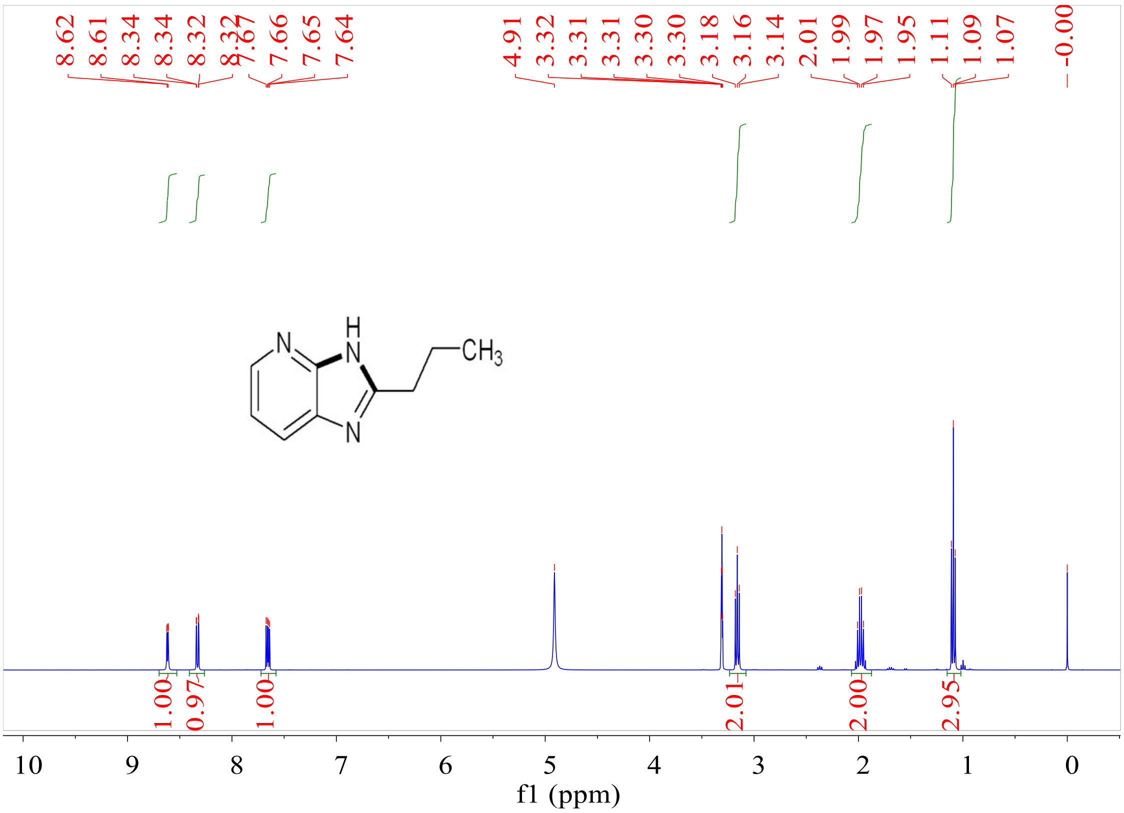

Figure S40 ^{1}HNMR of compound **2s** in CD_3OD

^{13}C NMR (101 MHz, CD_3OD) δ 160.5, 147.0, 144.8, 127.5, 125.6, 121.8, 30.4, 21.7, 13.8; HRMS (ESI): m/z $[M + H]^+$ calcd for $C_9H_{11}N_3$ 235.1077, found: 162.1023.

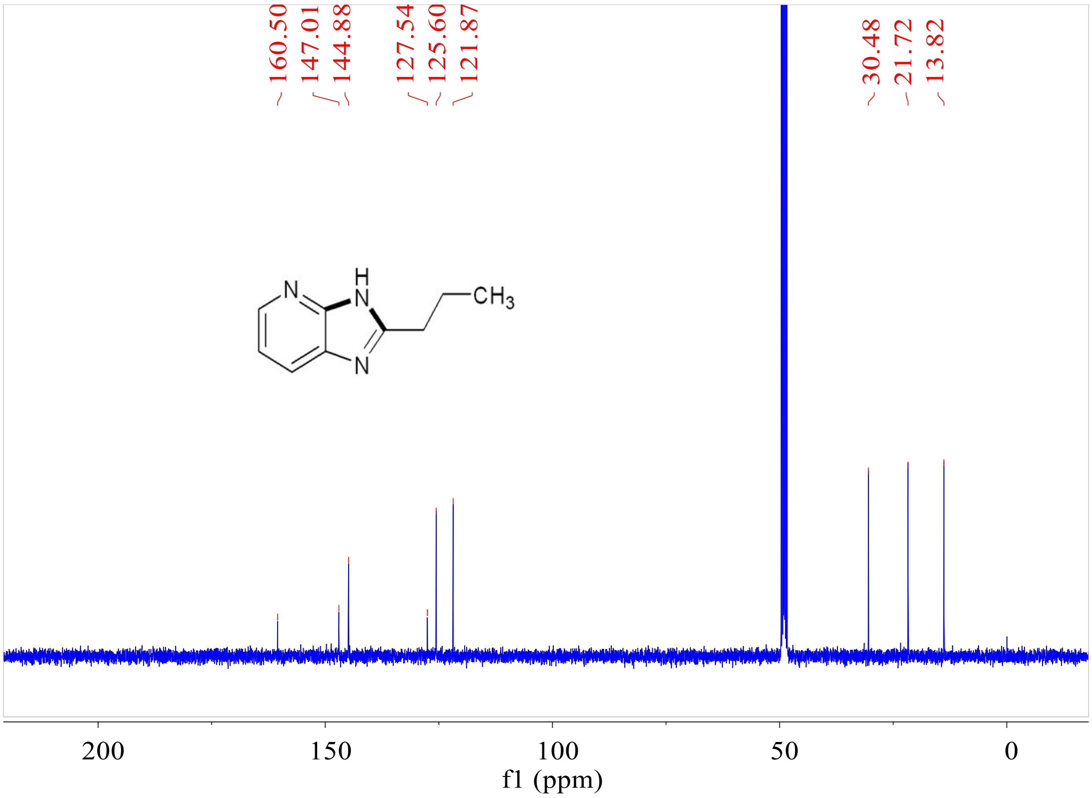

Figure S41 ^{1}HNMR of compound **2s** in CD_3OD

Benzimidazoles 2t

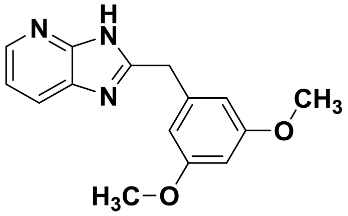

^{1}H NMR (400 MHz, CD_3OD) δ 8.30 (s, 1H), 7.91 (s, 1H), 7.25 (dd, *J* = 7.9, 4.9 Hz, 1H), 6.51 (d, *J* = 1.7 Hz, 2H), 6.37 (s, 1H), 4.86 (s, 3H), 4.19 (s, 2H), 3.74 (s, 6H), 3.31 (s, 3H).

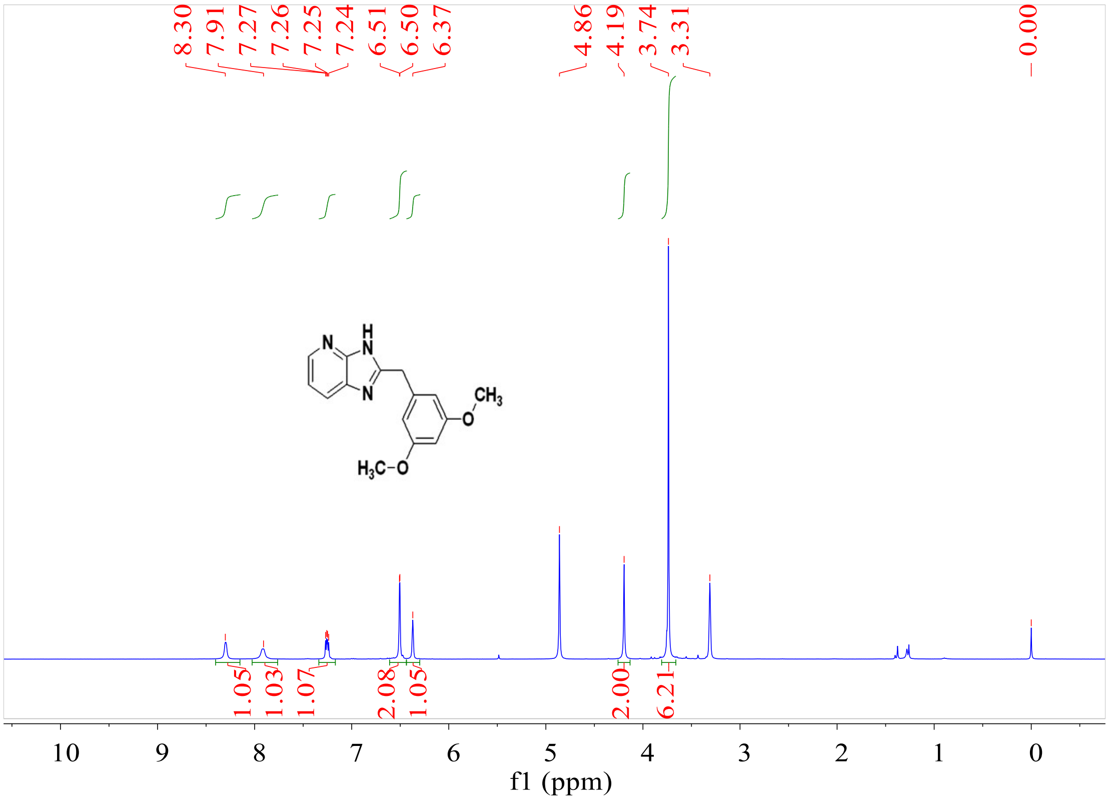

Figure S42 ^{1}HNMR of compound **2t** in CD_3OD

^{13}C NMR (101 MHz, CD_3OD) δ 162.7, 144.5, 139.6, 119.4, 107.9, 100.0, 55.7, 36.6. HRMS (ESI): m/z $[M + H]^+$ calcd for $C_{15}H_{16}N_3O_2$ 270.1237.

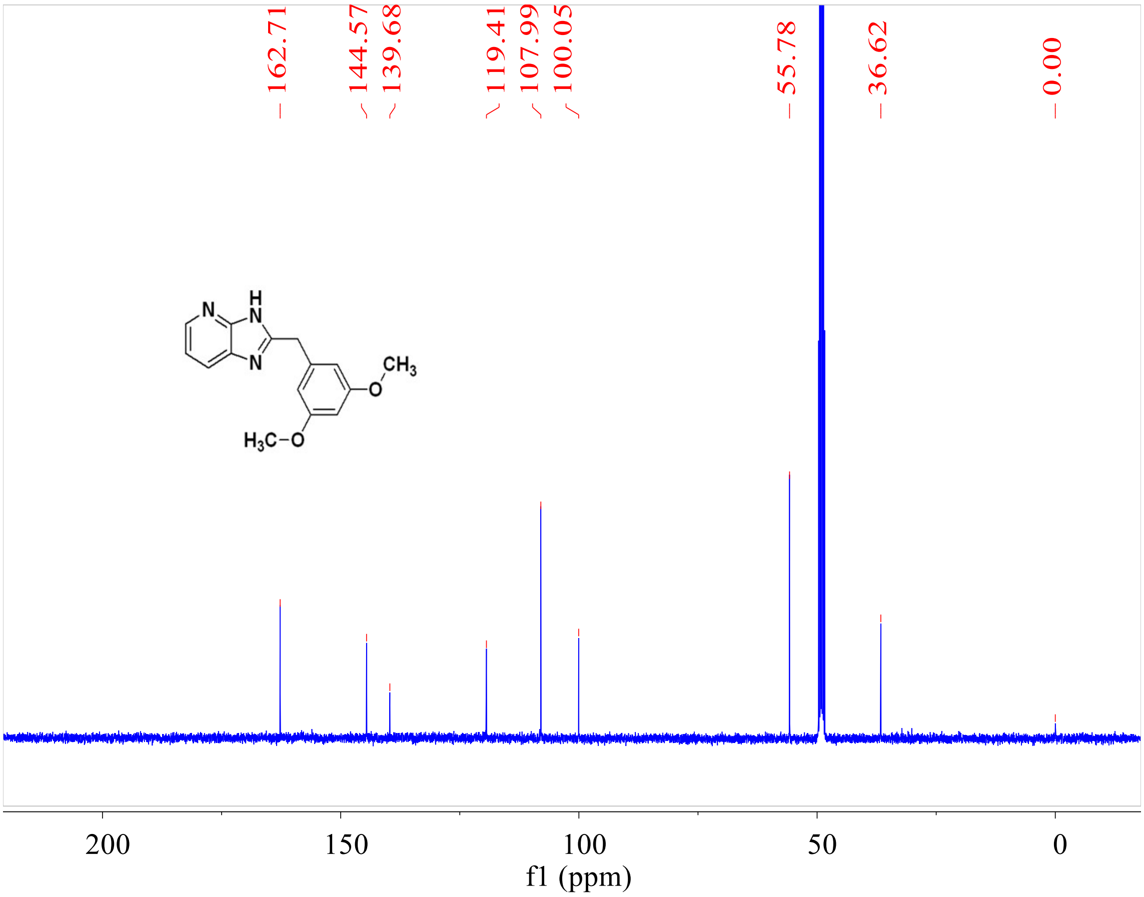

Figure S43 ^{13}CNMR of compound **2t** in CD_3OD

Benzimidazoles 2u

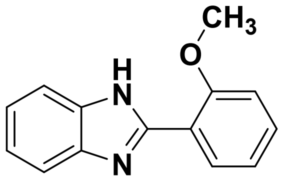

^{1}H NMR (400 MHz, CD_3OD) δ 8.24 (dd, *J* = 7.8, 1.7 Hz, 1H), 7.64 (dd, *J* = 6.0, 3.2 Hz, 2H), 7.47 (ddd, *J* = 8.7, 7.5, 1.7 Hz, 1H), 7.24 (dd, *J* = 6.1, 3.2 Hz, 2H), 7.19 (d, *J* = 8.3 Hz, 1H), 7.15 – 7.08 (m, 1H), 4.05 (s, 3H).

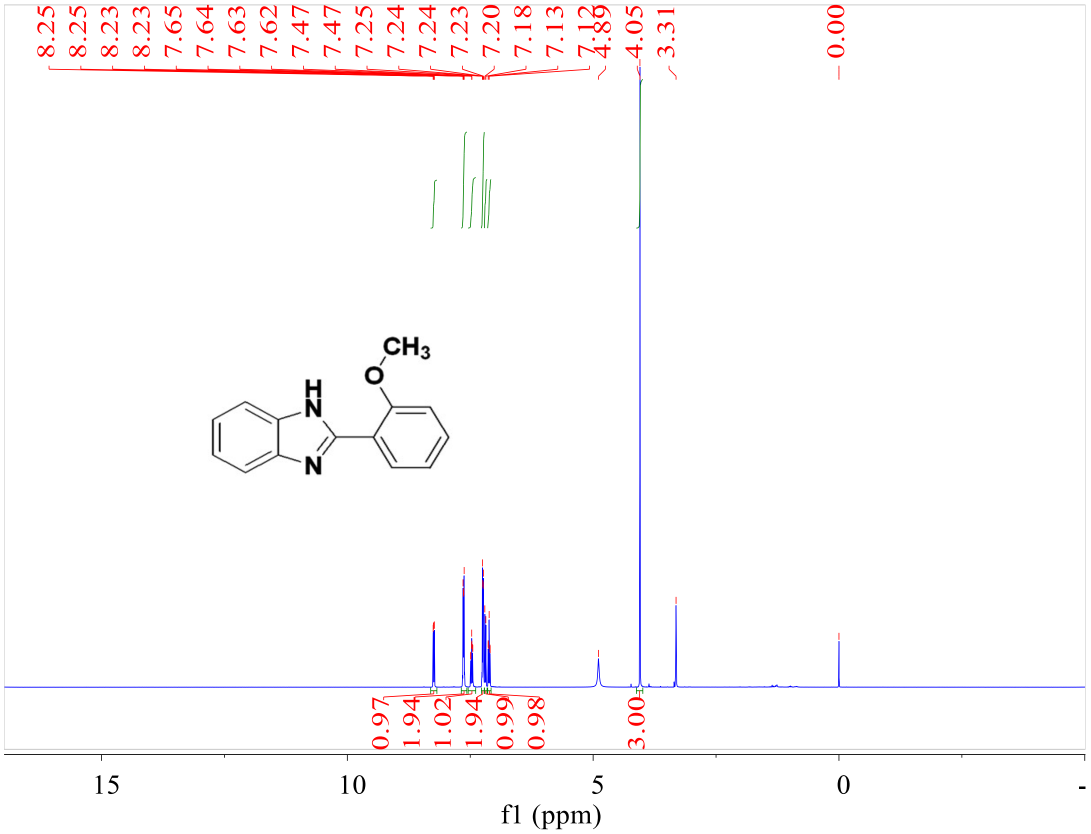

Figure S44 ^{1}HNMR of compound **2u** in CD_3OD

^{13}C NMR (101 MHz, CD_3OD) δ 157.3, 149.6, 138.1, 131.4, 129.5, 122.2, 120.7, 117.5, 114.4, 111.4, 54.8. HRMS (ESI): m/z $[M + H]^+$ calcd for $C_{14}H_{13}N_2O$ 225.1022

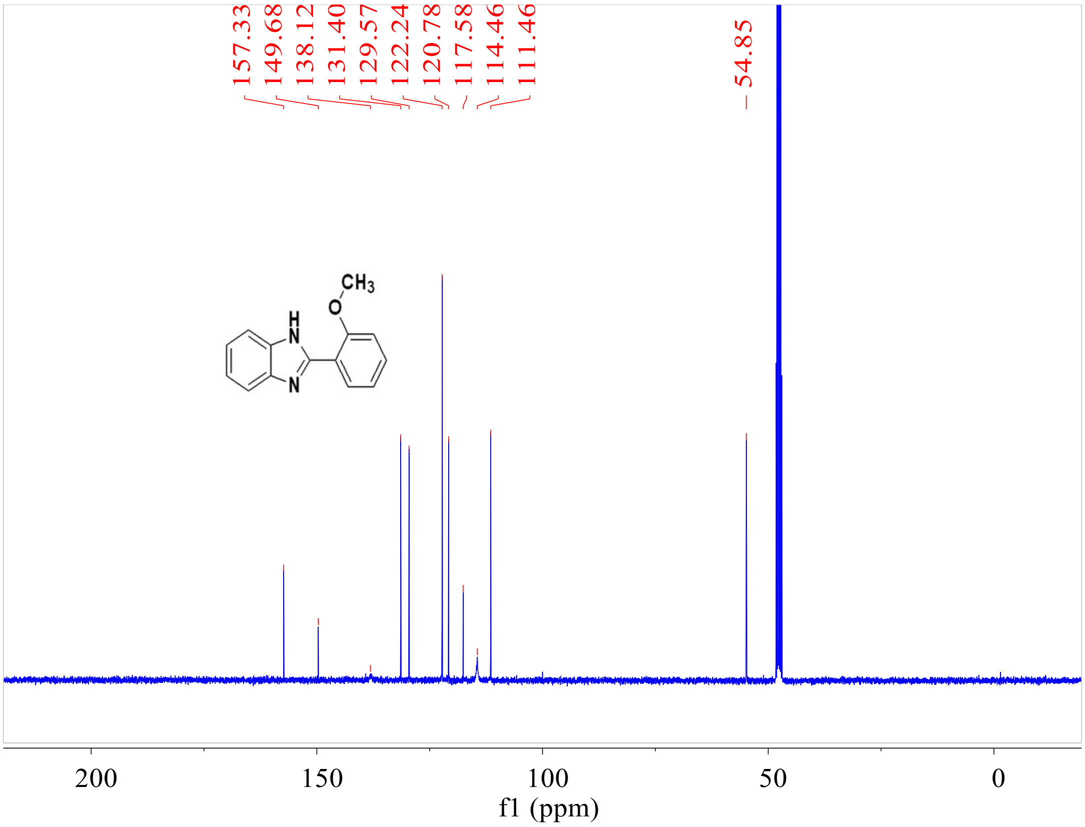

Figure S45 ^{13}CNMR of compound **2u** in CD_3OD

Benzimidazoles 2v

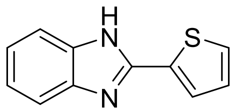

^{1}H NMR (400 MHz, CD_3OD) δ 8.20 (dd, *J* = 3.9, 1.1 Hz, 1H), 8.11 (dd, *J* = 5.0, 1.1 Hz, 1H), 7.78 (dt, *J* = 6.8, 3.4 Hz, 2H), 7.60 (dt, *J* = 6.2, 3.4 Hz, 2H), 7.43 (dd, *J* = 4.9, 3.9 Hz, 1H).

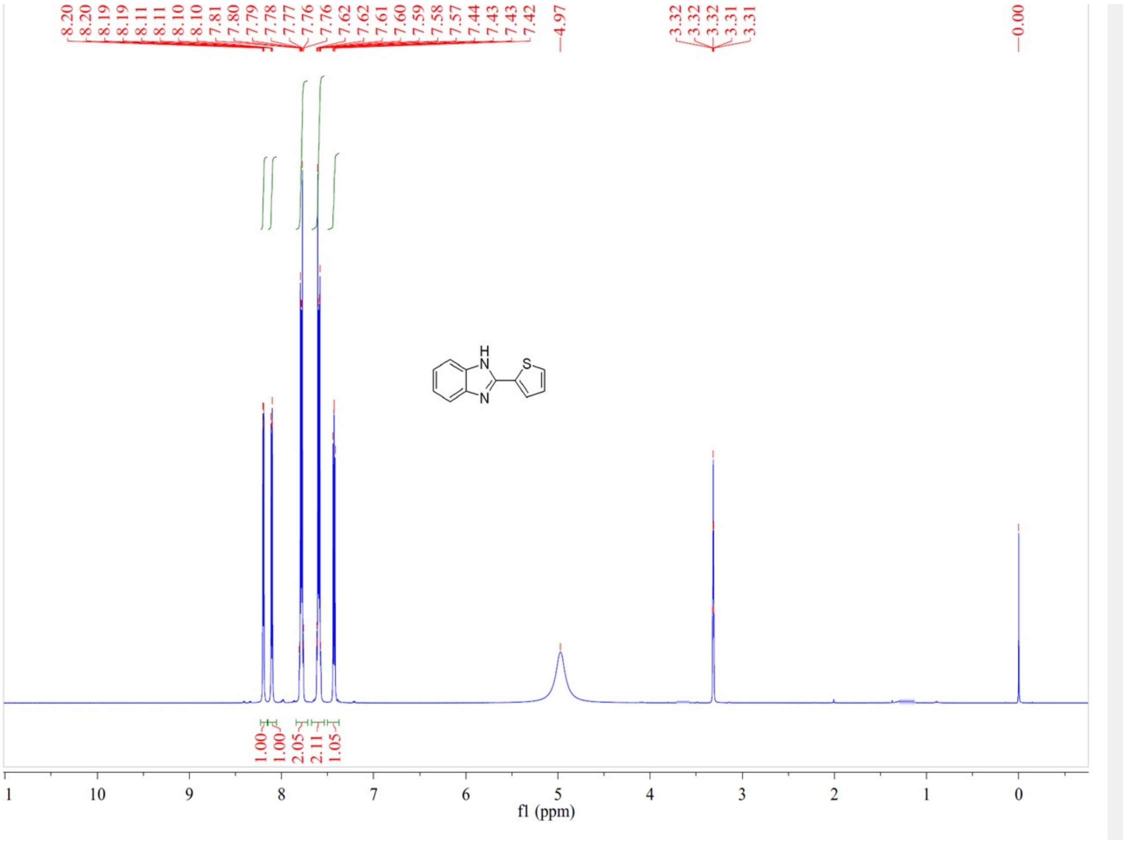

Figure S46 ^{1}HNMR of compound **2v** in CD_3OD

^{13}C NMR (101 MHz, CD_3OD) δ 145.8, 135.5, 134.5, 132.7, 130.6, 127.8, 124.9, 114.7. HRMS (ESI): m/z $[M + H]^+$ calcd for $C_{11}H_9S_2$ 201.0481

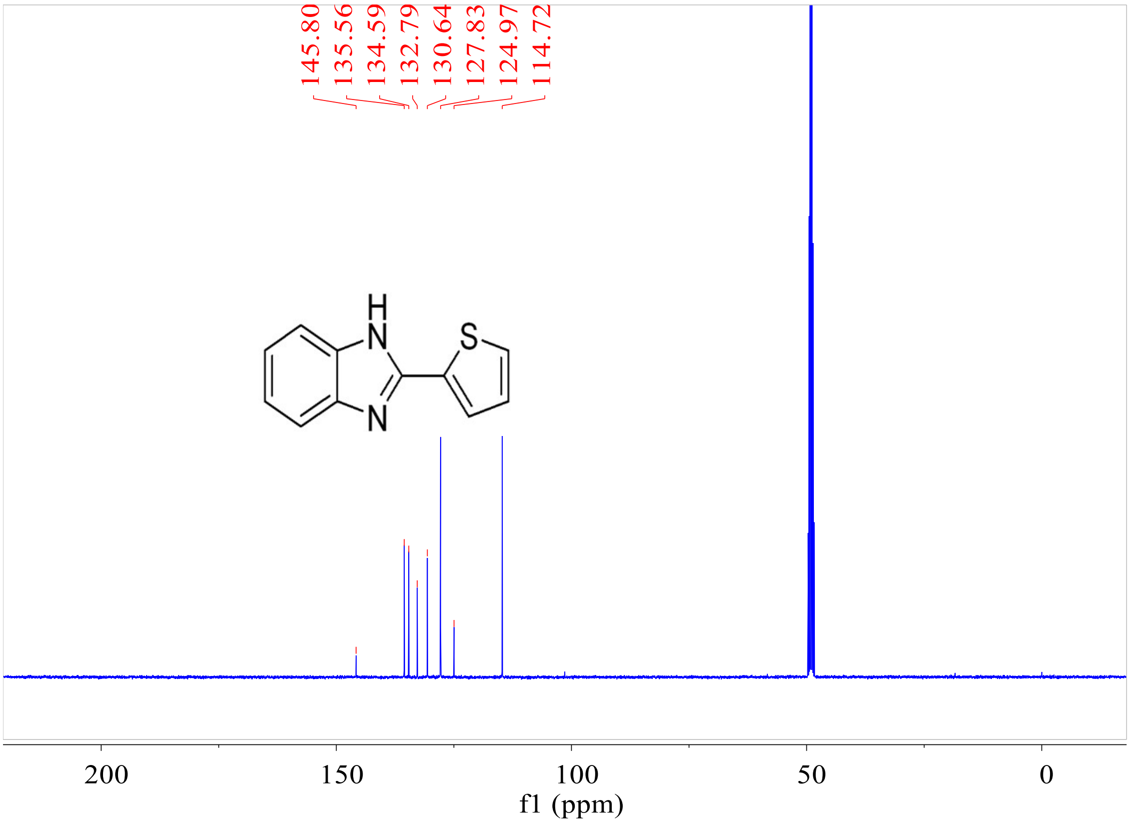

Figure S47 ^{13}CNMR of compound **2v** in Methano CD_3OD

Benzimidazoles 2w

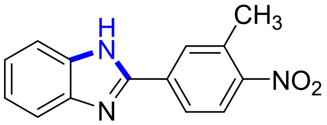

^{1}H NMR (400 MHz, DMSO-d_6) δ 13.33 (s, 1H), 8.31 (s, 1H), 8.25 – 8.16 (m, 2H), 7.66 (dd, J = 5.9, 3.2 Hz, 2H), 7.32 – 7.23 (m, 2H), 2.64 (s, 3H)

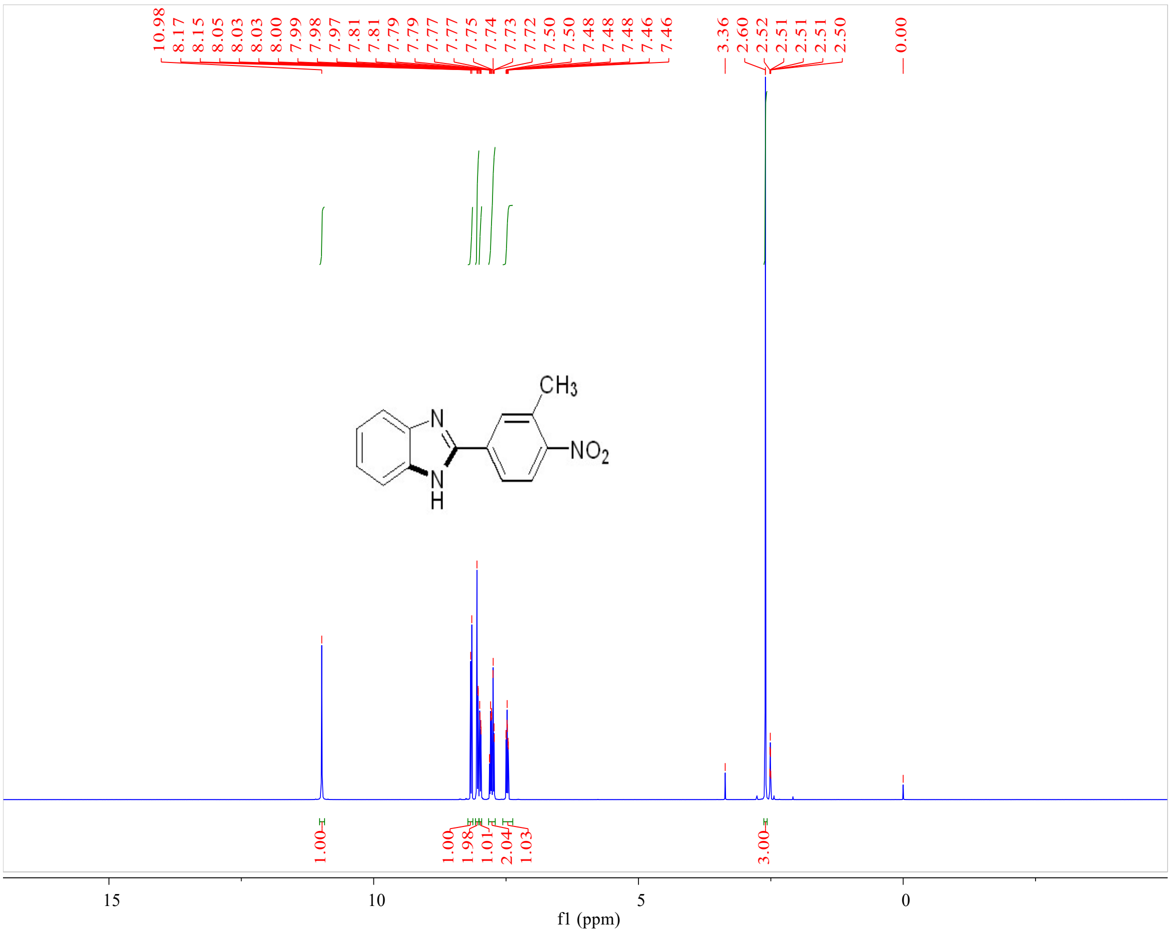

Figure S48 ^{1}HNMR of compound **2w** in DMSO-d_6

$^{13}C\{^{1}H\}$ NMR (100 MHz, DMSO-d_6) δ 149.4, 149.3, 139.9, 134.7, 134.1, 130.8, 130.7, 125.8, 125.3, 123.2, 115.9, 20.3; HRMS (ESI) m/z $[M + H]^+$ calcd for $C_{14}H_{12}N_3O_2$ 254.0924, found 254.0919.

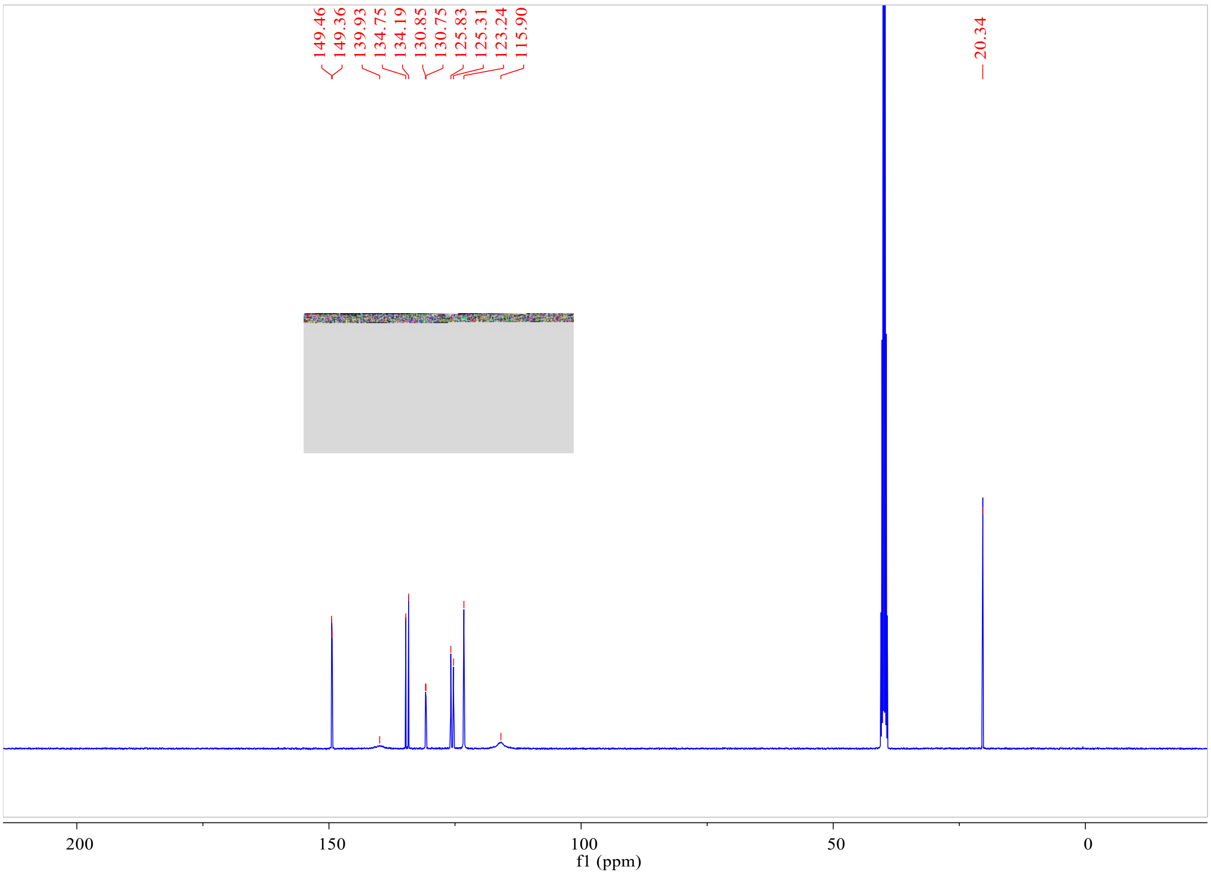

Figure S49 ^{13}CNMR of compound **2w** in DMSO-d_6

Benzimidazoles 2x

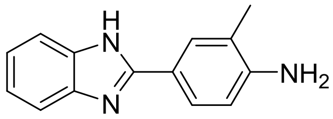

^{1}H NMR (400 MHz, DMSO-d_6) δ 12.40 (s, 1H), 7.81 – 7.66 (m, 3H), 7.47 (s, 2H), 7.10 (dd, J = 5.9, 3.2 Hz, 2H), 6.69 (d, J = 8.3 Hz, 1H), 5.35 (s, 2H), 2.14 (s, 3H).

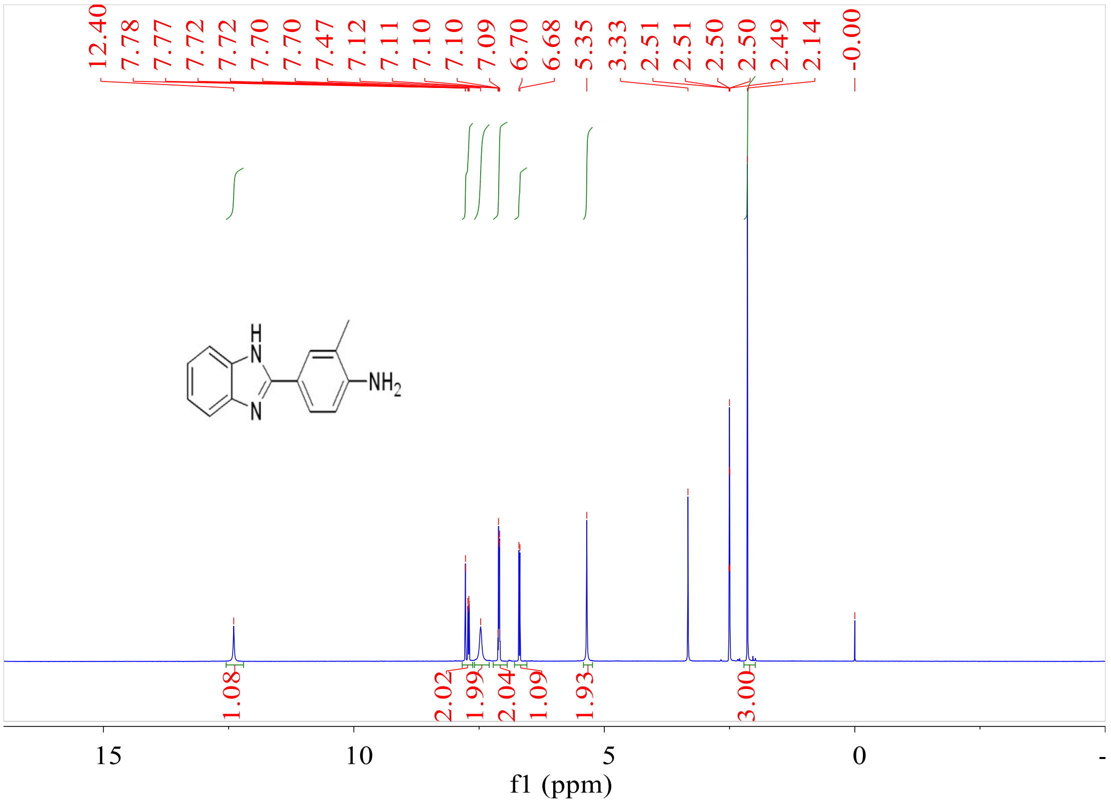

Figure S50 ^{1}HNMR of compound **2x** in DMSO-d_6

^{13}C NMR (101 MHz, DMSO) δ 154.7, 148.7, 143.1, 137.1, 131.6, 128.3, 121.9, 121.9, 121.2, 118.77, 117.6, 113.8, 110.4, 32.2, 17.92.HRMS (ESI): m/z $[M + H]^+$ calcd for $C_{14}H_{14}N_3$ 224.1128

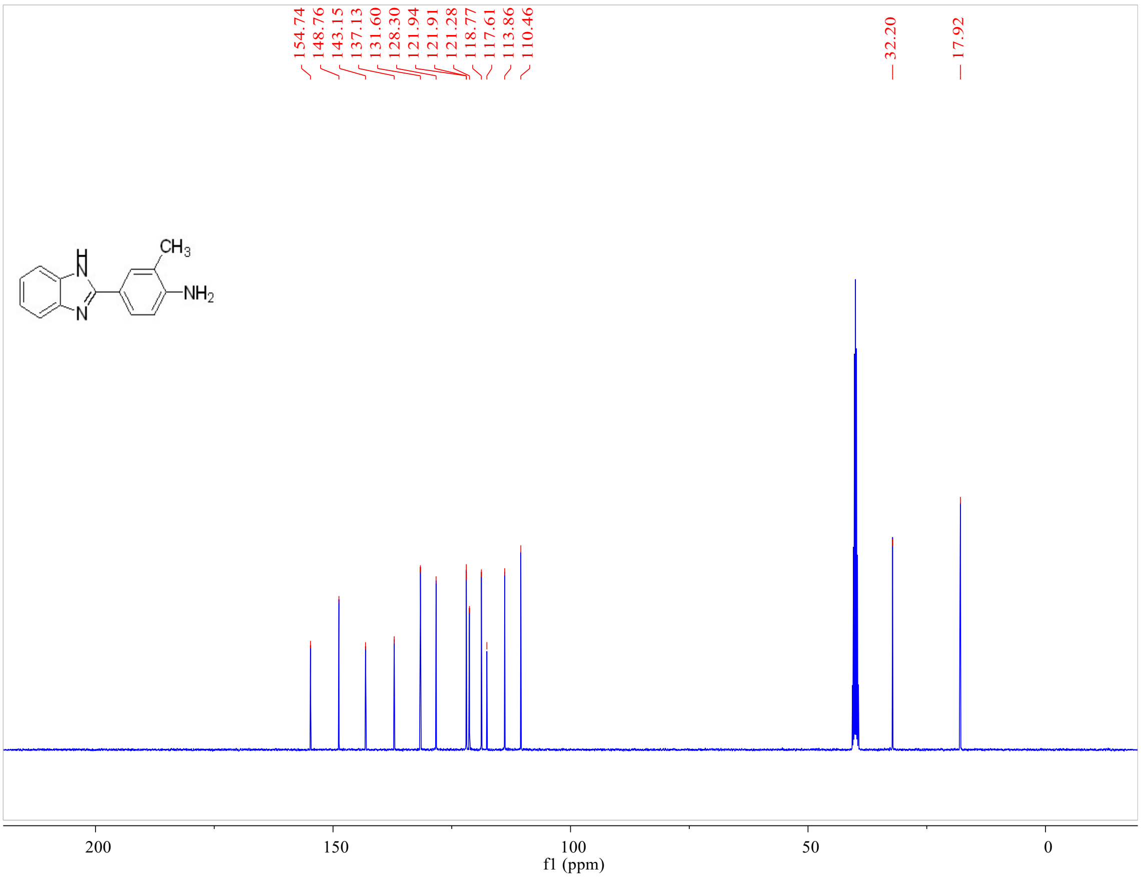

Figure S51 ^{13}CNMR of compound **2x** in DMSO-d_6

Benzimidazoles 2y

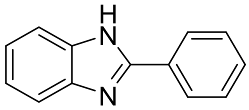

^{1}H NMR (400 MHz, DMSO-d_6) δ 12.90 (s, 1H), 8.30 – 8.03 (m, 2H), 7.67 – 7.45 (m, 5H), 7.33 – 6.96 (m, 2H).

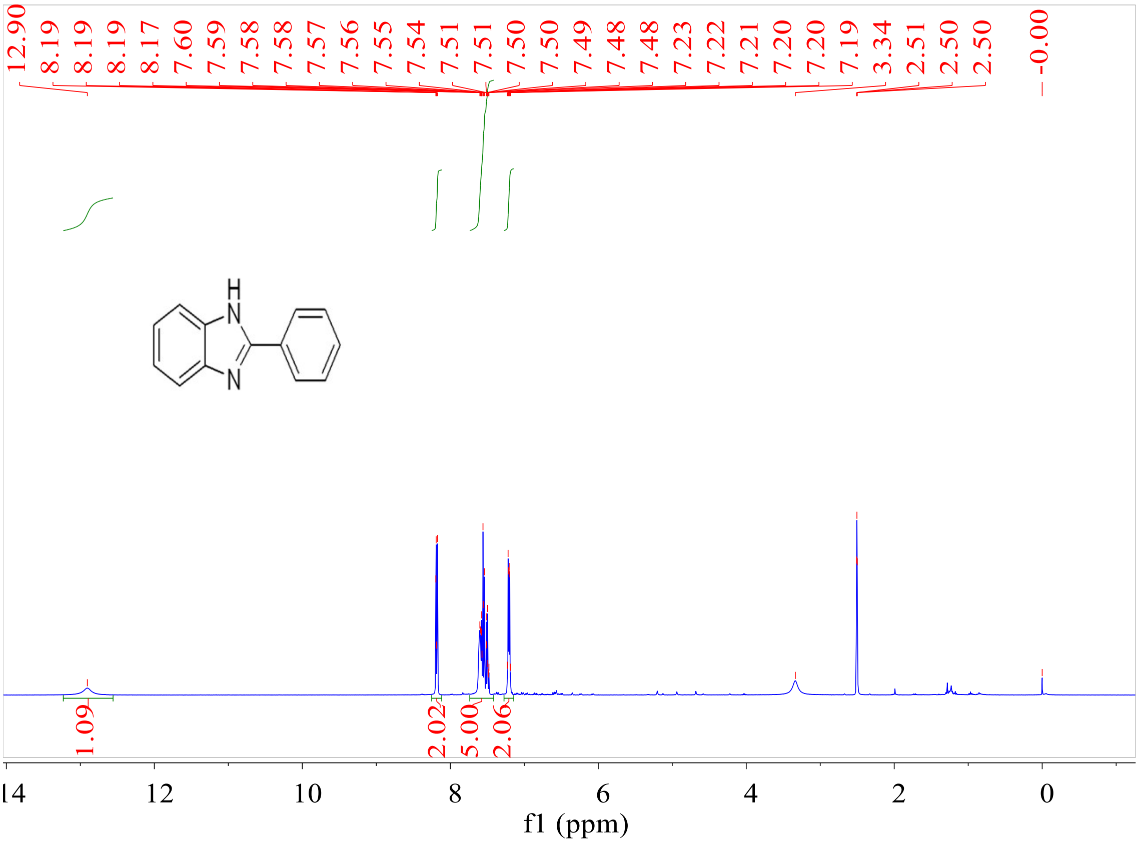

Figure S52 ^{1}HNMR of compound **2y** in DMSO-d_6

^{13}C NMR (101 MHz, DMSO) δ 151.6, 130.6, 130.3, 129.4, 126.8, 122.5; HRMS (ESI): m/z [M + H]$^+$ calcd for $C_{13}H_{11}N_2$ 195.0917

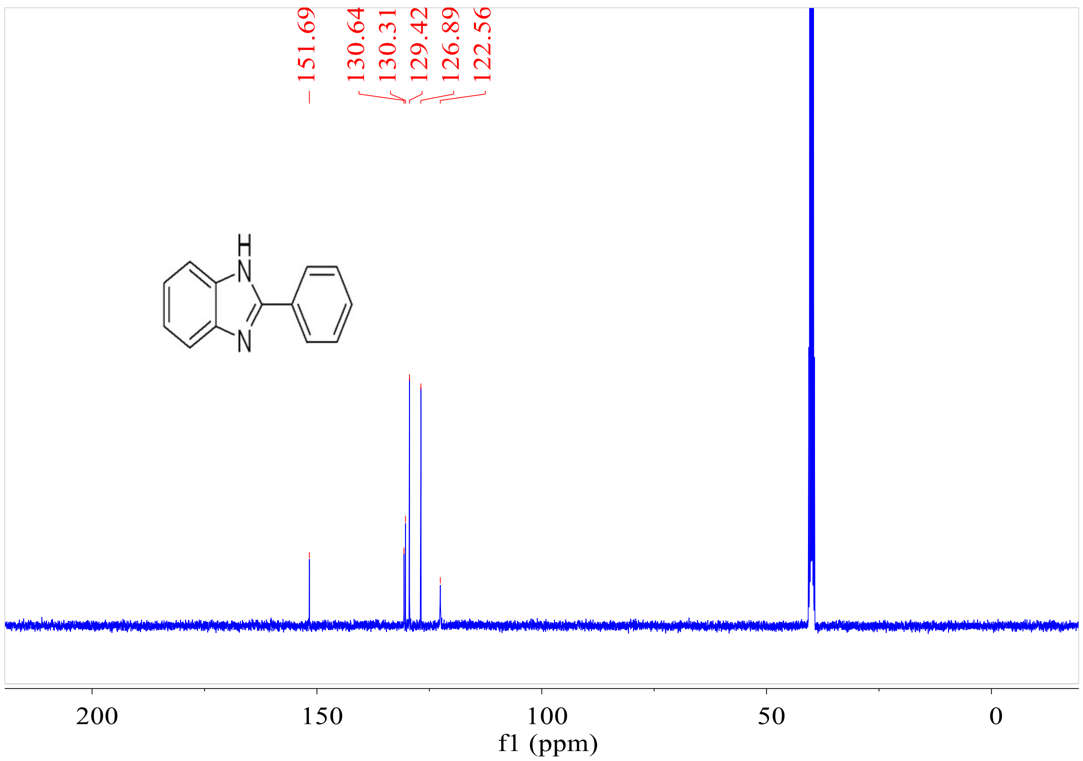

Figure S53 ^{13}CNMR of compound **2y** in DMSO-d_6

Benzimidazoles 2z

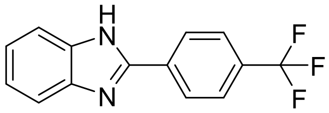

^{1}H NMR (400 MHz, CD_3OD) δ 8.36 (d, *J* = 8.2 Hz, 2H), 8.08 (d, *J* = 8.3 Hz, 2H), 7.90 (dd, *J* = 6.2, 3.2 Hz, 2H), 7.68 (dd, *J* = 6.2, 3.1 Hz, 2H).

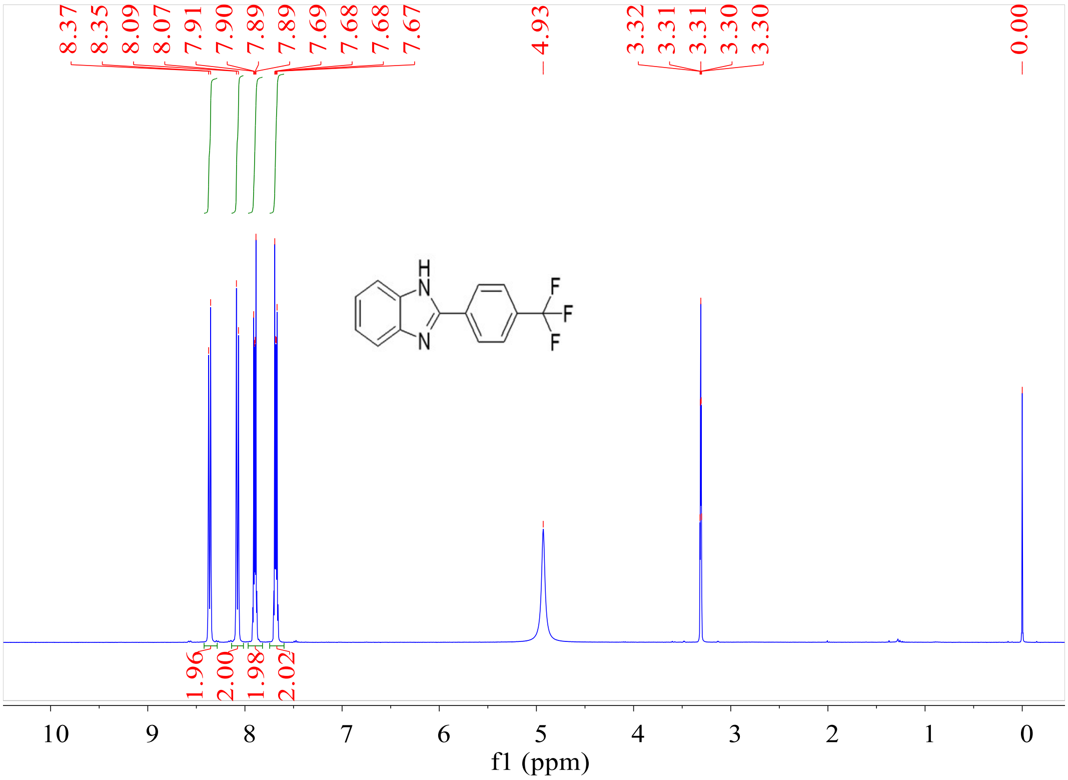

Figure S54 ^{1}HNMR of compound **2z** in CD_3OD

^{13}C NMR (101 MHz, CD_3OD) δ 149.3, 135.9, 135.6, 133.2, 130.1, 128.3, 128.1, 128.1, 128.0, 127.9, 127.9, 126.2, 123.5, 115.2, HRMS (ESI): m/z $[M + H]^+$ calcd for $C_{14}H_{10}F_3N_2$ 263.0791

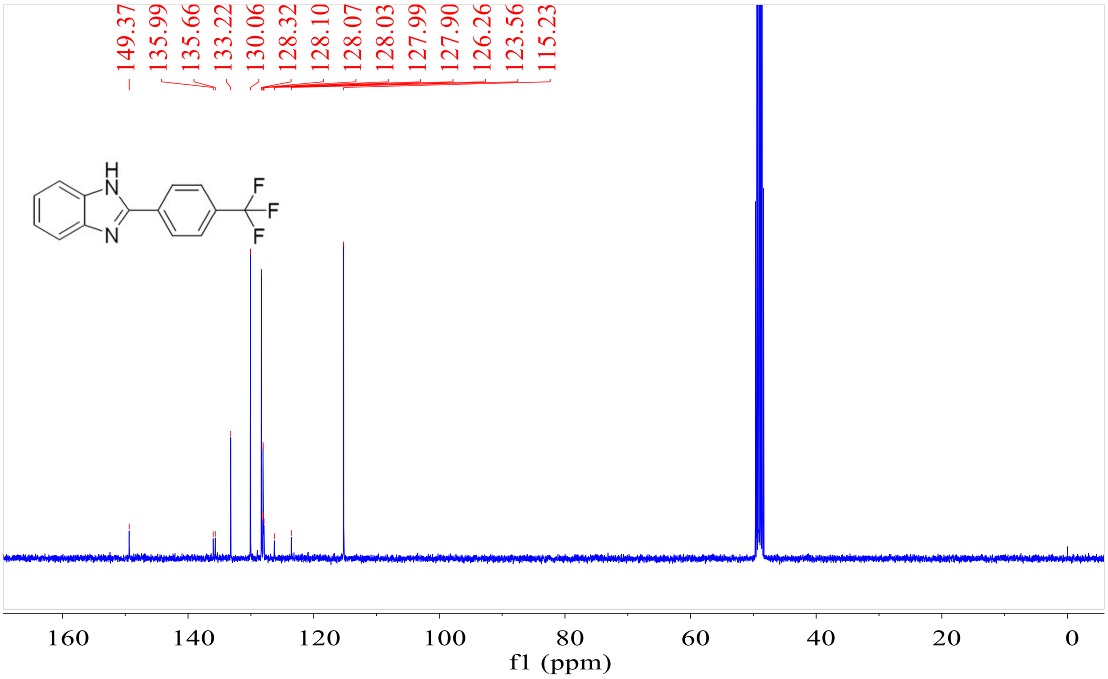

Figure S55 ^{13}CNMR of compound **2z** in CD_3OD

^{19}F NMR (377 MHz, CD_3OD) δ -62.90, -63.13, -63.61.

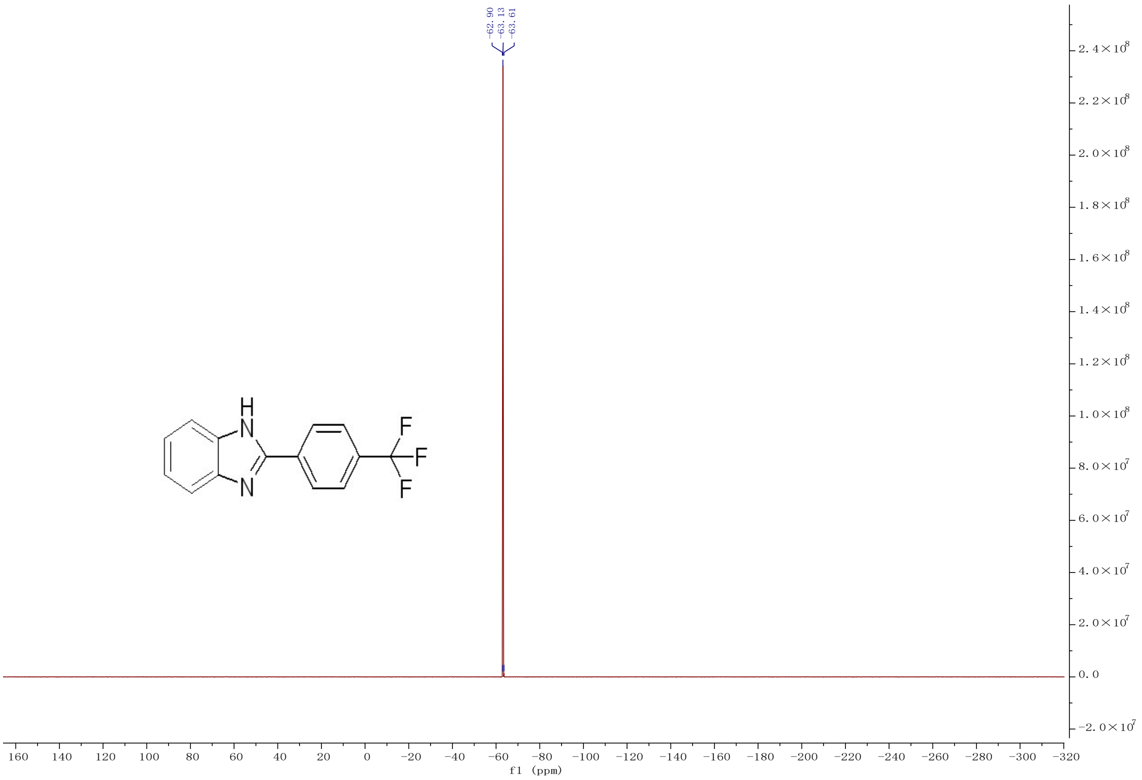

Figure S56 ^{19}F NMR of compound **2z** in CD_3OD

Benzimidazoles 2aa

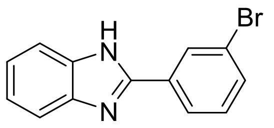

^{1}H NMR (400 MHz, DMSO-d_6) δ 13.33 – 12.82 (s, 1H), 8.42 – 8.33 (t, J = 1.7 Hz, 1H), 8.22 – 8.14 (dt, J = 7.8, 1.1 Hz, 1H), 7.74 – 7.68 (ddd, J = 8.0, 1.9, 0.9 Hz, 1H), 7.66 – 7.57 (s, 2H), 7.57 – 7.47 (t, J = 7.9 Hz, 1H), 7.30 – 7.15 (m, 2H).

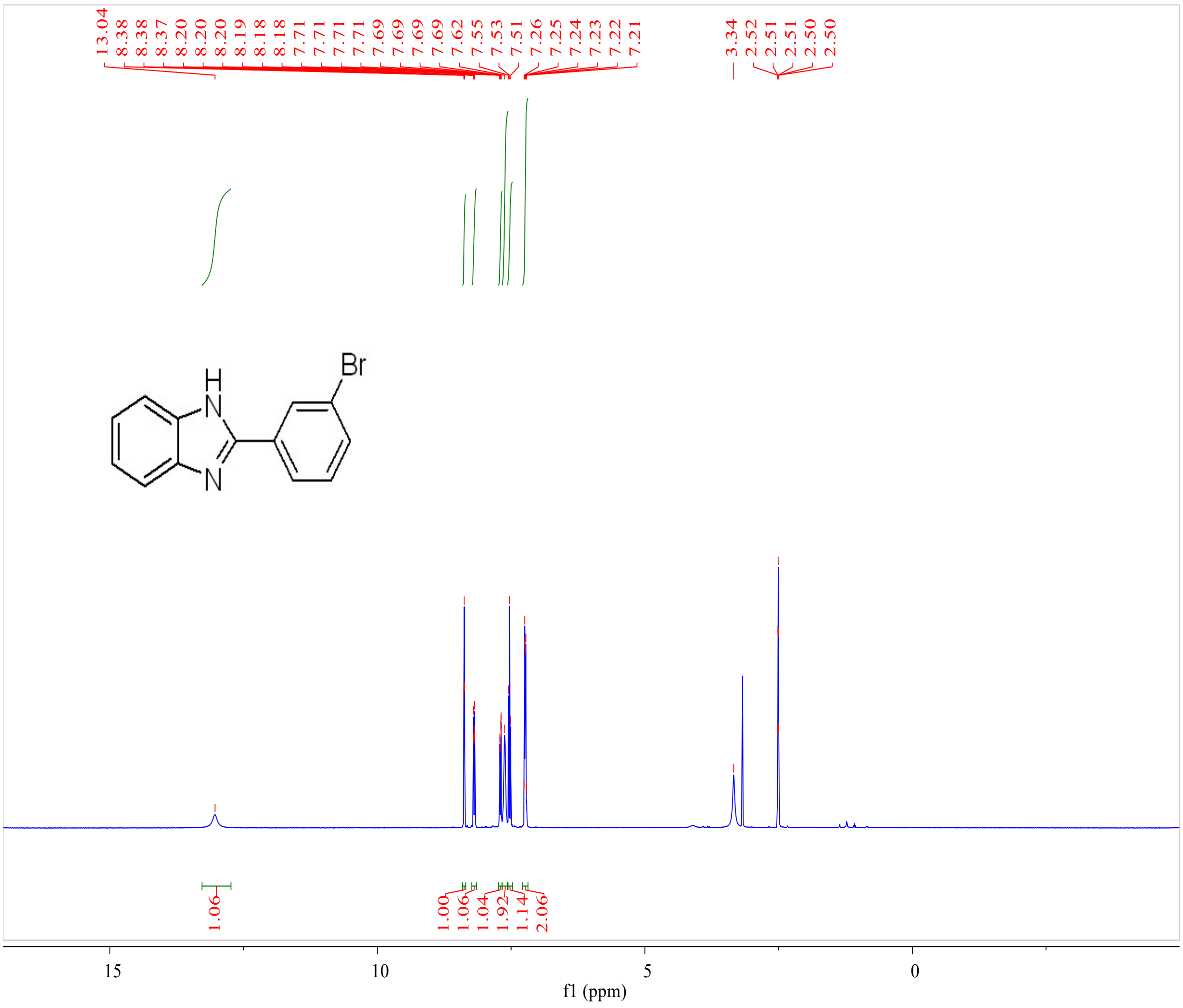

Figure S57 ^{1}HNMR of compound **2aa** in DMSO-d_6

^{13}C NMR (101 MHz, DMSO) δ 150.1, 132.9, 132.8, 131.6, 129.3, 125.8, 122.7, HRMS (ESI): m/z [M + H]$^{+}$ calcd for $C_{13}H_{10}BrN_2$ 273.0022

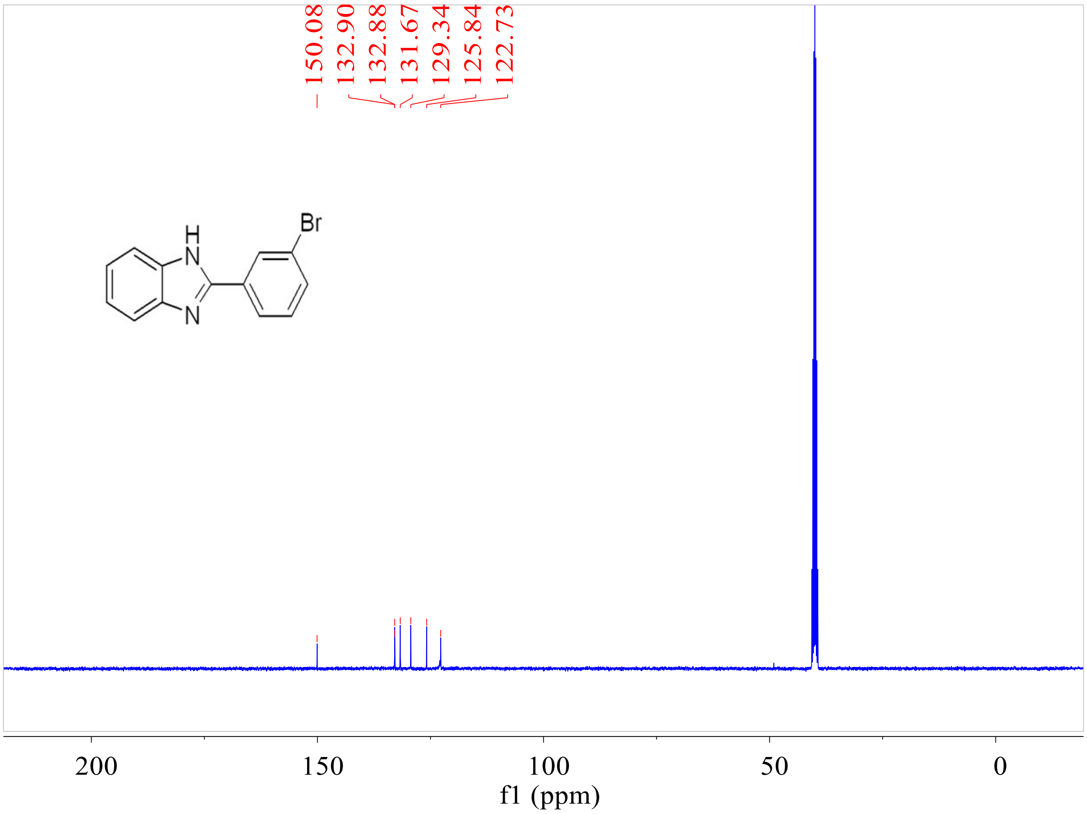

Figure S58 ^{13}CNMR of compound **2aa** in DMSO-d_6

Benzimidazoles 2bb

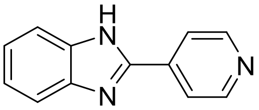

^{1}H NMR (400 MHz, CD_3OD) δ 8.69 (d, *J* = 5.0 Hz, 2H), 8.12 – 7.96 (m, 2H), 7.64 (dd, *J* = 5.9, 3.2 Hz, 2H), 7.40 – 7.22 (m, 2H).

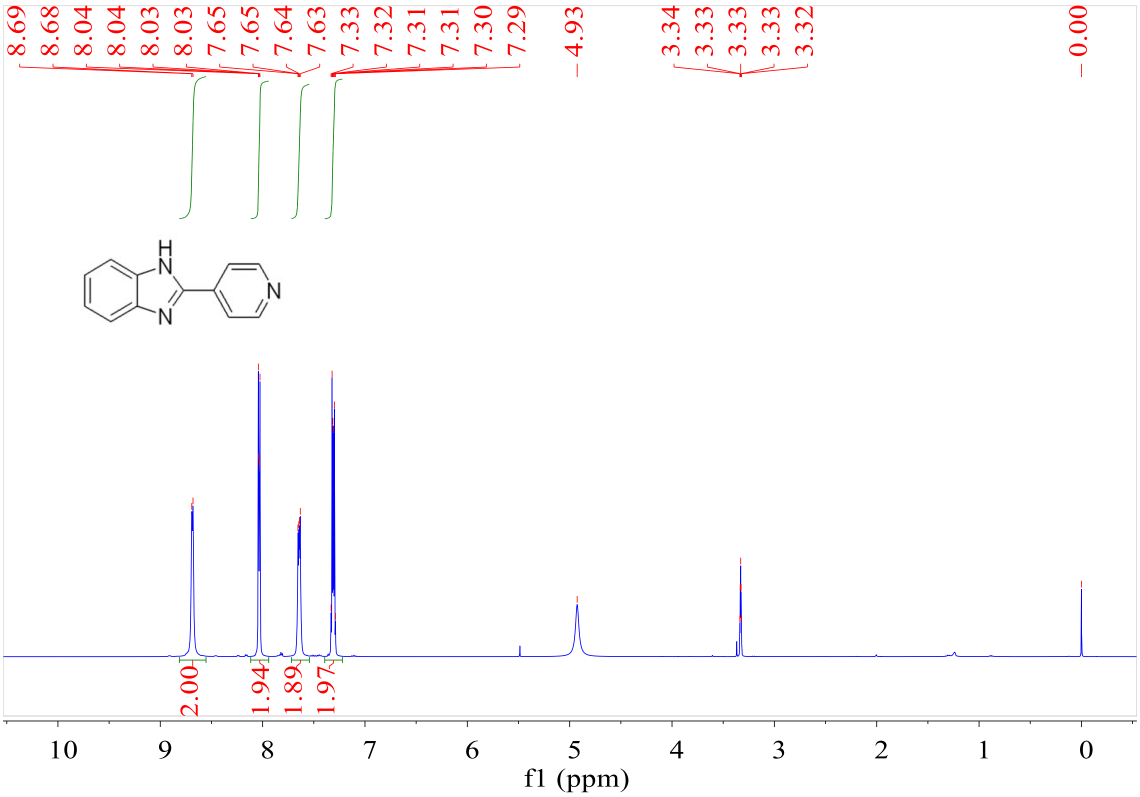

Figure S59 ^{1}HNMR of compound **2bb** in CD_3OD

^{13}C NMR (101 MHz, CD_3OD) δ 151.1, 150.0, 139.2, 124.8, 122.1, 116.6, 112.7, HRMS (ESI): m/z $[M + H]^+$ calcd for $C_{12}H_{10}N_3$ 196.0896

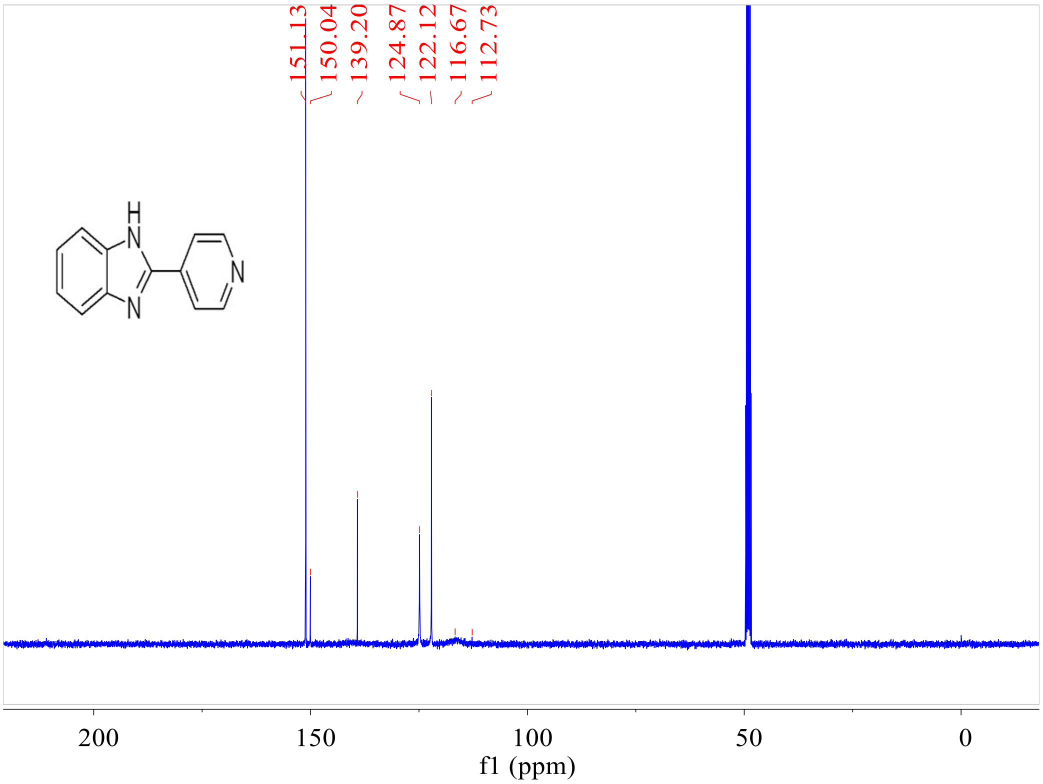

Figure S60 ^{13}CNMR of compound **2bb** in CD_3OD

Benzimidazoles 2cc

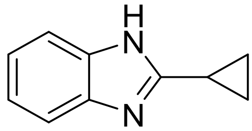

^{1}H NMR (400 MHz, $CDCl_3$) δ 7.51 (dd, J = 6.0, 3.2 Hz, 2H), 7.19 (dd, J = 6.0, 3.2 Hz, 2H), 2.16 – 1.99 (m, 1H), 1.35 – 1.19 (m, 2H), 1.18 – 0.97 (m, 2H).

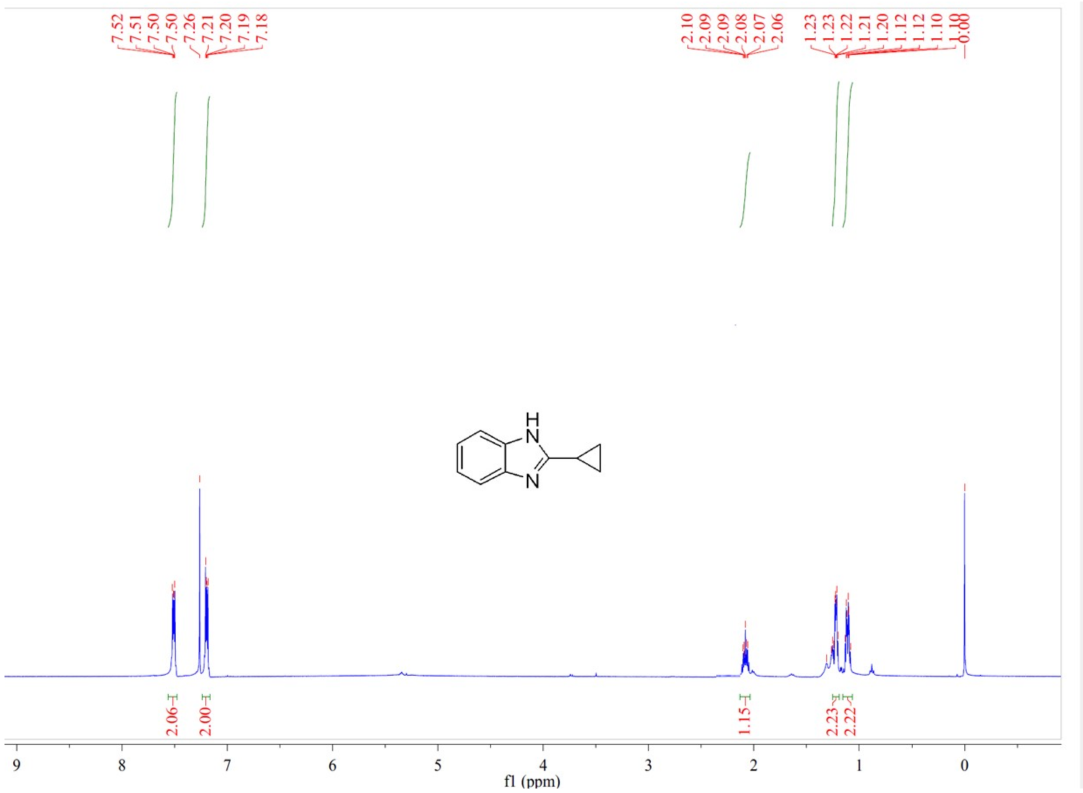

Figure S61 ^{1}HNMR of compound **2cc** in $CDCl_3$

^{13}C NMR (101 MHz, $CDCl_3$) δ 156.5, 135.5, 122.2, 114.3, 9.6, 8.8, -0.01. HRMS (ESI): m/z [M + H]$^+$ calcd for $C_{10}H_{11}N_2$ 159.0917.

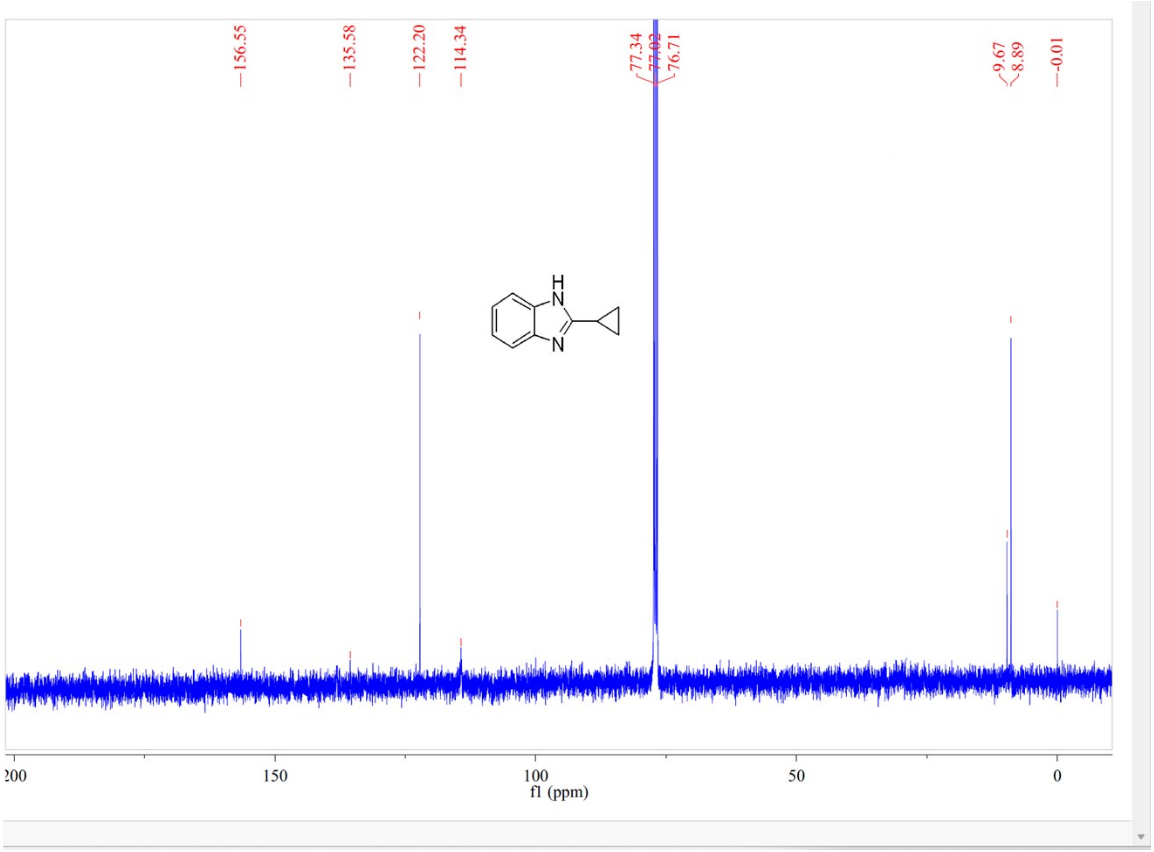

Figure S62 ^{13}CNMR of compound **2cc** in $CDCl_3$

Bendazol

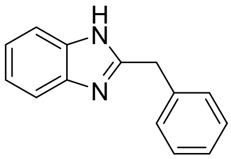

^{1}H NMR (400 MHz, DMSO-d_6) δ 15.53 (s, 1H), 7.77 (dt, J = 6.7, 3.4 Hz, 2H), 7.62 – 7.46 (m, 4H), 7.44– 6.86 (m, 3H), 4.57 (s, 2H).

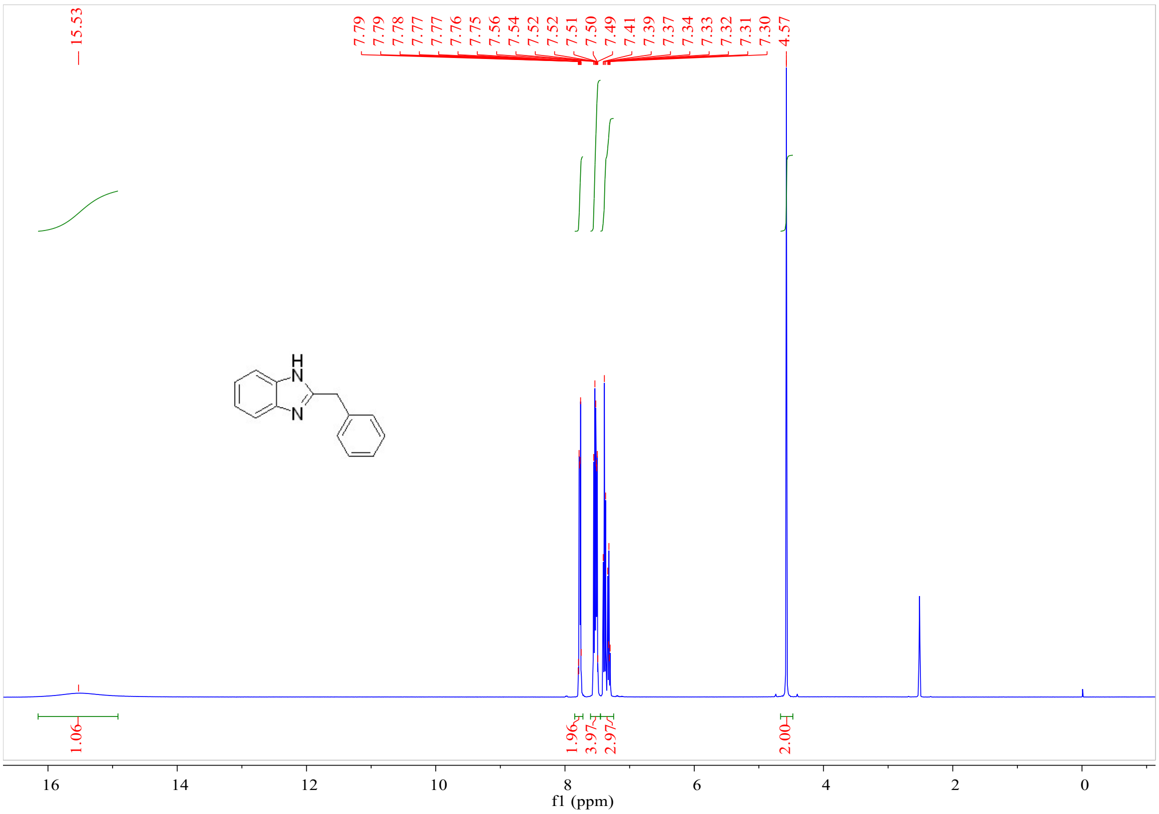

Figure S63 ^{1}HNMR of bendazol in DMSO-d_6

^{13}C NMR (101 MHz, DMSO) δ 152.5, 134.0, 130.7, 129.1, 128.9, 127.6, 125.5, 113.8, 31.8.

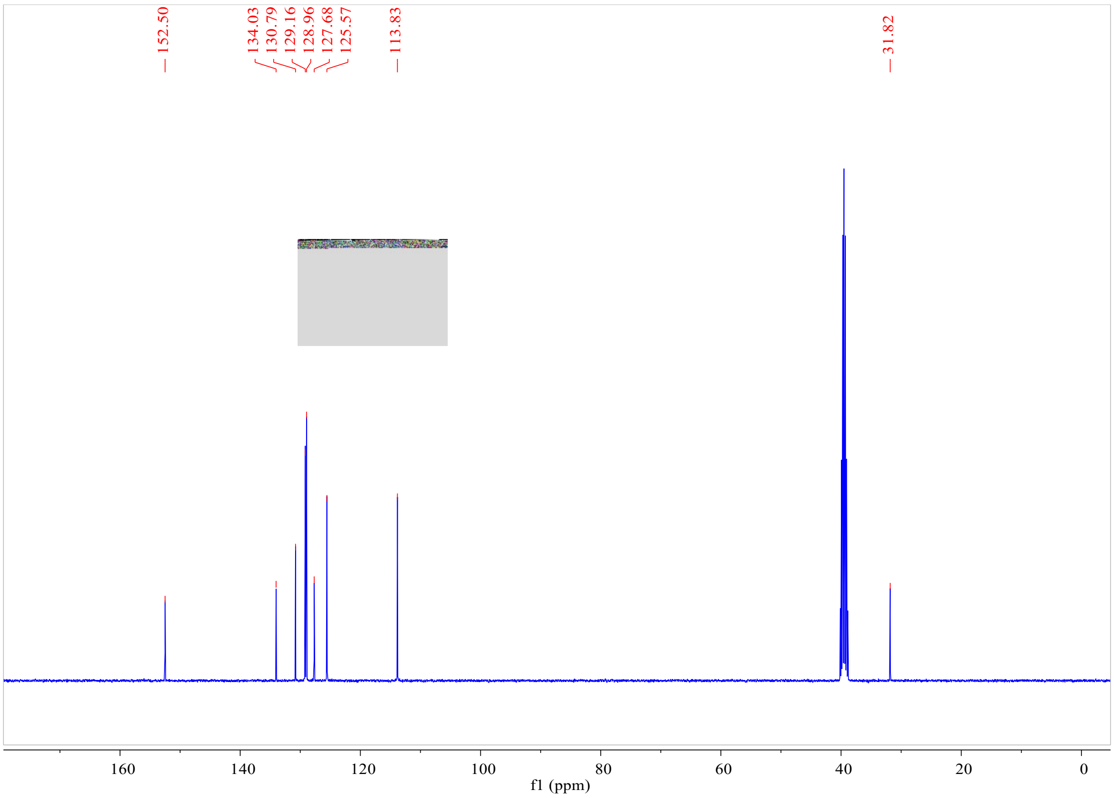

Figure S64 ^{13}CNMR of bendazol in DMSO-d_6

Thiabendazole

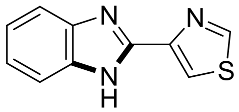

^{1}H NMR (400 MHz, DMSO-d_6) δ 13.00 (s, 1H), 9.34 (d, J = 2.0 Hz, 1H), 8.47 (d, J = 1.9 Hz, 1H), 7.61 (dd, J = 55.4, 7.1 Hz, 2H), 7.23 (dt, J = 8.6, 4.9 Hz, 2H).

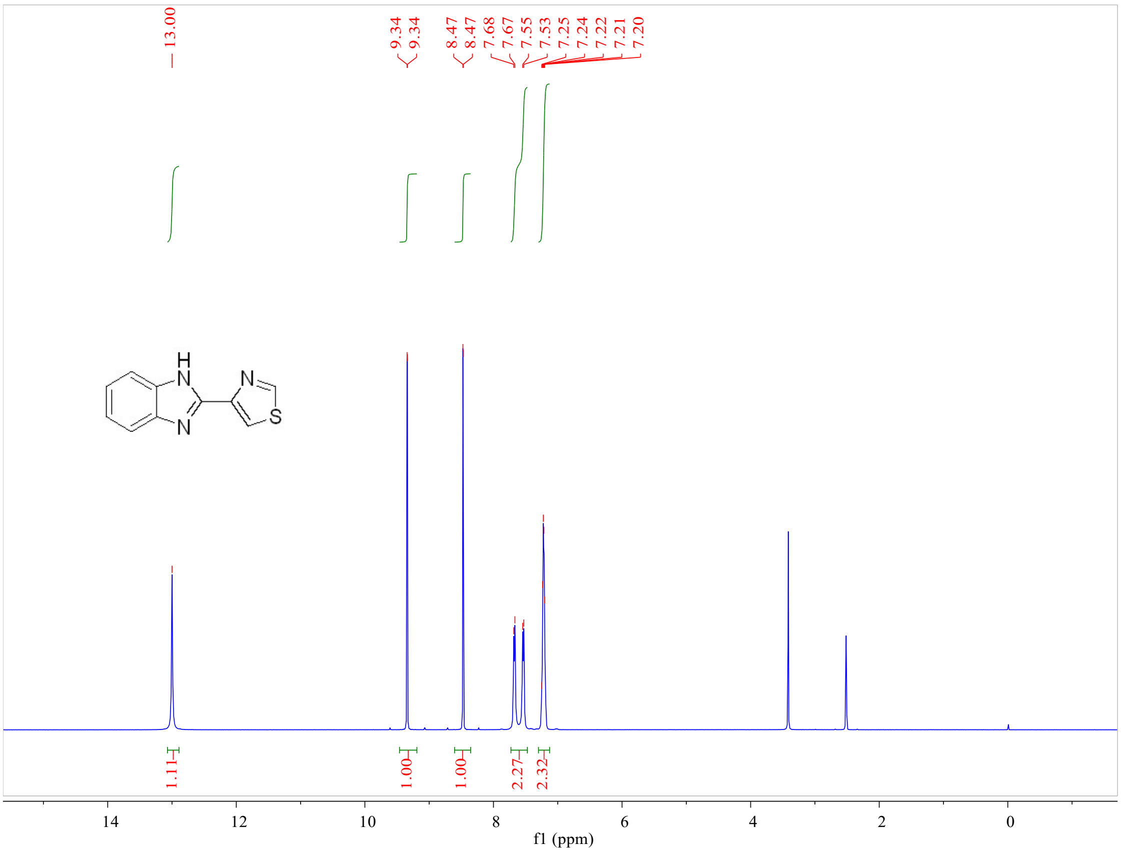

Figure S65 ^{1}HNMR of thiabendazole in DMSO-d_6

^{13}C NMR (101 MHz, DMSO) δ 155.5, 147.0, 146.9, 143.7, 134.3, 122.5, 121.7, 119.3, 118.7, 111.7.

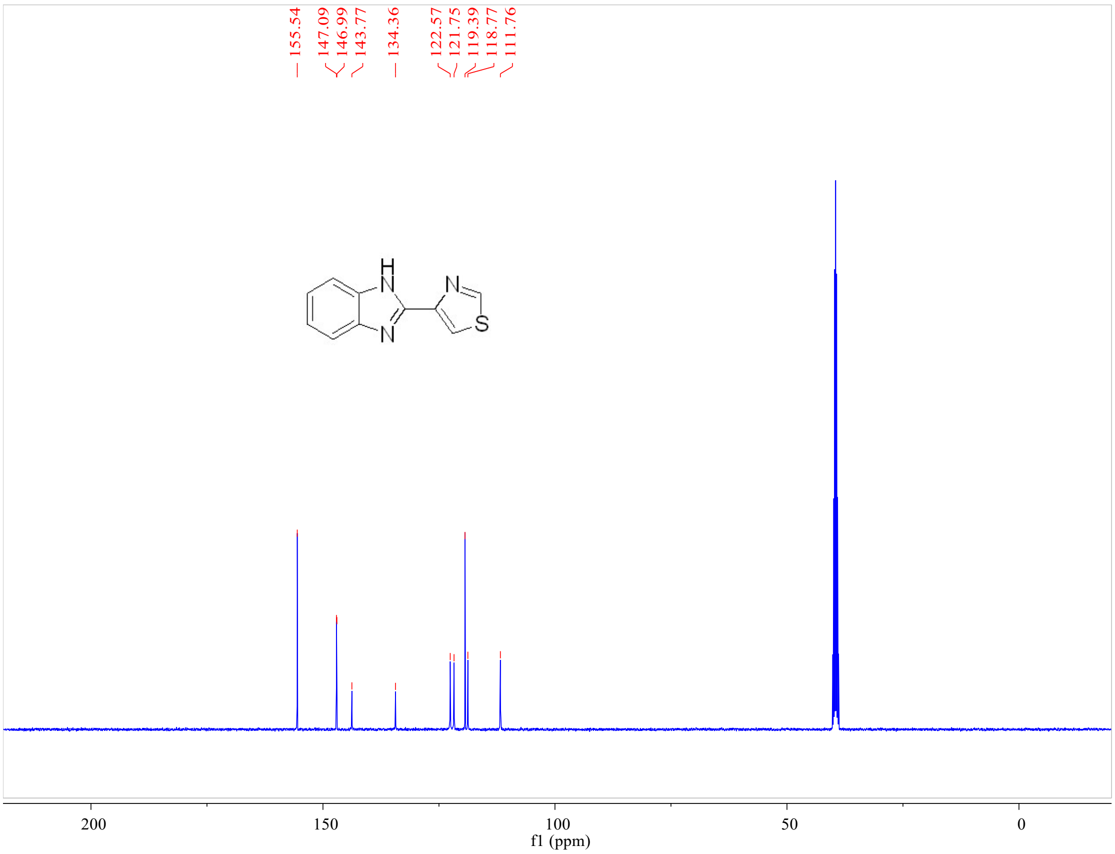

Figure S66 ^{13}CNMR of thiabendazole in DMSO-d_6
